# Just-in-time Database-Driven Web Applications

**DOI:** 10.2196/jmir.5.3.e18

**Published:** 2003-08-29

**Authors:** Kenneth R Ong

**Keywords:** Database applications

## Abstract

"Just-in-time" database-driven Web applications are inexpensive, quickly-developed software that can be put to many uses within a health care organization. Database-driven Web applications garnered 73873 hits on our system-wide intranet in 2002. They enabled collaboration and communication via user-friendly Web browser-based interfaces for both mission-critical and patient-care-critical functions. Nineteen database-driven Web applications were developed. The application categories that comprised 80% of the hits were results reporting (27%), graduate medical education (26%), research (20%), and bed availability (8%). The mean number of hits per application was 3888 (SD = 5598; range, 14-19879). A model is described for just-in-time database-driven Web application development and an example given with a popular HTML editor and database program.

## Introduction

Content management for intranet managers and developers can be challenging. Updating content on static HTML pages can be time consuming. Database-driven Web applications (DDWA) are one significant methodology that can be used to empower end users to change content dynamically without knowledge of HTML or an HTML editor. This tutorial poses a design-and-development model for "just-in-time" DDWA that can quickly develop applications. An example is given with a popular HTML editor and database program.

Intranets in and outside of health care organizations commonly provide access to policies, procedures, document archives, manuals, and other information [1,2]. In a first-generation intranet, such content is static and requires skill with HTML or an HTML editor. Any alterations of, additions to, or deletions from content require a Web developer. Maintaining such a site can be daunting when faced with ongoing requests to change content. Turn-around time for posting new content may be compromised. Information critical to the business or to patient care may be delayed with attendant affects.

Adding dynamic information that can be managed directly by delegated managers or superusers can enhance the value of intranets and eliminate the time and effort that would otherwise be needed to deliver the same content using the services of a Web developer. DDWA can enable managers to post information and knowledge directly to an intranet without having to know HTML or an HTML editor. DDWA are the means to the "content management" that characterizes the current generation of intranets. Content management is offered either as third-party software or as fee-for-service programming — the costs of these may be a barrier for many not-for-profit health care organizations. The decision to "buy or build" has several considerations (Table 1). Return-on-investment or payback period analyses often have difficulty demonstrating convincing hard returns on such investments regardless of the size of the organization. In-house-built DDWA are an alternative to third-party solutions. Vendor services are often expensive and subsequent modifications or new applications incur additional expense. Shrink-wrapped software for content management may not be flexible enough to adapt easily and the learning curve required to master the software can be even more demanding than learning the Active Server Page (ASP) script itself needed to build DDWA. (An Active Server Page is an HTML page that includes one or more small embedded programs that are processed on a Web server before the page is sent to the user.) Yet, the aphorism still applies, "buy when you can, build when you must" [3].

**Table 1:** Factors to consider when deciding to buy or build software

**Buy**	**Build**
Shorter time to implementation Reduced risk Greater resources and skills for development One-time costs for startup may be greater Product less flexible	Control over design, development, and implementation Full control over code Time and costs of developing or acquiring resources and skills in-house Upgrade at time of your choosing Not susceptible to the marketplace changes that a vendor is subject to

Database-driven Web applications have changed the face of software development. Prior to the Web and DDWA, rapid application development (RAD) methodology and software had reduced the software cycle from years to months. DDWA have reduced the development cycle even further, from months to hours. Database-driven Web applications are the latest evolution of rapid application development [4]. DDWA have been described in the medical literature for procedure logs [5]. This paper describes an aggressive version of DDWA development we have labeled "just-in-time DDWA."

## Methods

### Database-driven Web Applications on an Integrated Delivery System's Intranet

Saint Vincent Catholic Medical Centers (SVCMC) is one of the New York metropolitan-area's larger health care systems, serving over 500000 people annually. It was established in 2000 as a result of the merger of Catholic Medical Centers of Brooklyn and Queens, Saint Vincent's Hospital and Medical Center of New York, and Sisters of Charity Healthcare in Staten Island. Saint Vincent Catholic Medical Centers serves as the academic medical center of New York Medical College in New York City. The system includes 8 hospitals. Over 3000 physicians are affiliated with the system, which includes 4 skilled nursing facilities, 3 home care agencies, a hospice, and over 60 ambulatory care clinics. The trauma center in Manhattan was the major trauma center for the World Trade Center on September 11, 2001.

**Table 2:** List of just-in-time database-driven Web applications

**Category**	**Application**	**Description**
Bed availability	Nursing home	One staff person centrally administers the application for 4 facilities
Bed availability	Behavioral health	One staff person at each of 5 facilities administers application
Continuing medical education	Anthrax update	Pilot project for continuing medical education on the intranet
Finance	Expense code, expense list, and item master	Three applications for finance-related data (read-only access)
Graduate medical education	Sign-out roster and resident evaluation	The online intern patient sign-out roster, faculty evaluations, and a nondatabase-related ASP file upload application comprise a suite of applications for the internal-medicine residency training programs
Graduate medical education	Scholarly activity	Offering and tracking clinical research opportunities for residents
Human resources	Job description and performance appraisal	An application designed to facilitate standardization of job descriptions and performance-appraisal forms from 3 service divisions
Human resources	Online staff training: class scheduling and post-tests	Two applications for behavioral-health staff training, one to schedule instructor-led classes, the other to test online self-learning classes
Payer relations	Announcement regarding managed care plans	Updates from the Payer Relations Office regarding managed care. The 6 most-recent notices are posted on their intranet page via a server side include
Pharmacy	Formulary	As per Joint Commission on Accreditation of Healthcare Organizations (JCAHO) guidelines, an online formulary merges the separate formularies of 3 service divisions into 1 searchable database. Pharmacy can update, delete, and add medications to the database
Phone directory	System-wide, behavioral health, and success agenda contact list	Three applications: 3 separate databases for the system, the behavioral health product line, and a managerial work group, respectively
Physician credentialing	Delineation of privileges	A read-only view of one service division's physician credentialing
Research	Prostate cancer screening	A database for the summer prostate cancer screening campaigns started in 1999
Results reporting	HIV viral load	Intranet-based patient results reporting of HIV viral loads from the organization's centralized virology laboratory to the system

**Table 3:** Applications with Web-server security groups

**Web Server Security Groups**	**Members**
Behavioral health beds	5
Behavioral health phone	4
Board	1
Available nursing home beds	1
Faculty Manhattan	120
Faculty Staten Island	44
Faculty Brooklyn-Queens	27
Graduate Medical Education (GME) Director's Office	17
Homecare	5
Post-graduate Year (PGY) 2002	28
Post-graduate Year (PGY) 2003	45
Post-graduate Year (PGY) 2004	81
Post-graduate Year (PGY) 2005	118
Pharmacy Directors	5
Prostate cancer screening	6
Scholar	1
Behavioral health training & staff development	2
HIV laboratory	26
Total	536

The Saint Vincent Catholic Medical Centers intranet was developed in 2000 at the time of the organization's merger and is described in detail elsewhere [6]. Intranet development seeks to serve both the business and patient-care missions of the organization. The intranet can facilitate system-wide collaboration and integration. It serves as the conduit for more than 200 online medical-knowledge resources, dozens of manuals, patient education, forms, training, and patient-results reporting. Physicians, managers, nurses, and other Saint Vincent Catholic Medical Centers associates have access to the intranet. Its resources are particularized for the various niche markets and communities within this audience.

The intranet is carried over a wide-area network connecting more than 6000 workstations. The same software is installed on all workstations, eg, Microsoft Office 2000, Internet Explorer 5, and Adobe Reader.

In calendar year 2002, the intranet garnered 1505865 hits, of which 160014 were to Active Server Pages. Of the Active Server Page hits, nearly half (73873) were to database-driven Web applications. The remaining hits to active-server pages include pages dedicated to restricted, intranet-file uploads and directories [7], and pages with server-side includes [8] that generate database-results views, like the payer-relations managed-care announcements. (Server side includes are the facility provided by an HTTP server to replace certain HTML tags in one HTML file with the contents of another file at the time the file is sent out by the server.)

Nineteen DDWA were developed (Table 2). In 2002, of the 73873 hits to DDWA, the application categories that comprised 81% of the hits were results reporting (27%), graduate medical education (26%), research (20%), and bed availability (8%) (Figure 1, in Multimedia Appendix 1). The mean number of hits per application was 3888 (SD = 5598; range, 14-19879).

The number of records in each database ranged widely, from 2 in the anthrax-update continuing-medical-education application to 23137 in the prostate-cancer screening application (mean = 2586; SD, 2319).

There were 35 separate Web server security groups (Table 3). An application may have up to 3 levels of security. Some read-only pages may be open to all and other pages may be open to a restricted group of individuals. Pages that permit updating records in, adding records to, or deleting records from a database are open only to application administrators. The average number of individuals in each group was 29.8 (SD = 14.8; range, 1-120). Each group is assigned either read-only or read-write access to a restricted sub-Web on the intranet. When an end user selects a hyperlink to a page on a restricted sub-Web, Windows 2000 queries the end user for the end-user's logon — ie, user name, password, and, if relevant, domain. The 272 internal-medicine residents across the system are grouped by year of graduation, facilitating entry of new interns and the departure of graduating residents each year.

### Phase 1: Project Selection and Planning

Just-in-time (JIT) DDWA development is a short but iterative process. Like other software-development cycles, just-in-time DDWA development includes at least 4 phases: (1) planning and design, (2) development, (3) testing, and (4) implementation. The process is distinctive for its short turn-around time and commitment to iterative cycles.

Choosing the right projects will improve the likelihood of success. The scope of the project should be limited to the possible. DDWA cannot meet the demands of a complex clinical-information system, such as a system for electronic medical records, a laboratory, or a pharmacy. If the estimated time to develop a project exceeds 8 hours, proceed cautiously, if at all. A project proposal that requires an interface falls outside the scope of a DDWA. Although the resources may be cost neutral, DDWA should serve interdivisional, product-line, or system-wide initiatives. A database with but one or two users would not usually justify a DDWA.

DDWA development teams are leaner and require greater collaboration and communication than traditional application-development teams. Planning and design involves the developer and 1 to 5 end users. Each application has a designated project sponsor who serves as the liaison for the given department or office. The project sponsor and/or superuser is committed to assisting in design, planning, testing, implementing, and, if need be, iterative cycles of testing and debugging. Contrary to earlier models for rapid application development, neither formal committee nor work groups nor multiple milestones are needed.

Team communication should incorporate a combination of one or more conference calls, meetings, e-mails, and "hallway consultations." All stakeholders in the process are aware that multiple cycles may be needed to arrive at an application that will satisfy the requirements of the project sponsor or the project sponsor's designee. The developer should anticipate multiple cycles of iterative testing and debugging. Yet, the developer should hope that the resulting creation, when appropriate, may inevitably be replaced by more-robust, more-efficient, and more-effective shrink-wrapped software.

### Phase 2: Server Administration

The server administrator is usually responsible for managing the server folders, creating the data connections, and defining security.

The tasks required to build DDWA include:

- Create new folders on the Web server with FrontPage or Windows 2000. If the application is to offer read-write access to a database, create another folder restricted to those with administrative rights.
- Define interim (or final) security on the access-control list of the folder with Windows 2000 (NTFS — the native file system of Windows NT) or a new server security group with Windows 2000 Server (MMC — Microsoft Management Console).
- Configure FrontPage Server Extensions, with Internet Information Server (IIS), to the new folder to create a new sub-Web. Managing security and recalculating links from the Active Server Pages to the database is quicker and easier in the microenvironment of a sub-Web than in a folder within the larger context of a parent Web.
- Create a new application, with IIS/MMC (Internet Information Server/Microsoft Management Console), with "script & executables" enabled with "Level I" security.
- Utilize "DSN-less" ("Data Source Name-less") data connection, which can simplify development and eliminate the task of building data-source names for the server administrator. Alternatively, the server administrator can create a data connection (system data-source name) on the Web server with Windows 2000 Server's Open Database Connections (ODBC).

If resources permit, a staging server for development is desirable. If not, a separate Web page on the intranet with hyperlinks to applications under development should suffice.

Full backup should occur weekly and incremental backups daily.

### Phase 3: Web Development

Our software development environment includes at a minimum FrontPage 2000, Notepad, Access 2000, and Adobe Photoshop 7. Other database and development software that typically require advanced skills are used infrequently in our DDWA development. SQL Server 2000 use was limited to its Data Transformation Services function to convert tables to Access or ASCII-delimited files. Visual InterDev was not used in development. The Web developer need not make a full-time commitment. The Web developer in our organization is its director of medical informatics, a full-time position that includes other information-systems responsibilities like health-care-provider liaison, system-software selection and implementation, and strategic planning. Site development and maintenance of both the Web and intranet sites constitute no greater than half his time in any given week.

Design and development for each DDWA typically requires 4 to 12 hours. DDWA that offer read-write access to a database require more time than read-only views.

Before creating the Active Server Pages that will comprise the DDWA, create a database in Microsoft Access with the data fields specified during planning and design. In the "Design" view of Access, define a primary key; make the data type of all data fields text; and set "Required" to "No."

Except for database applications that only require read-only access, a database-driven Web application will require Active Server Pages (ASP) devoted to adding, updating, deleting, and querying the database. Web pages devoted to the first 3 functions should reside in an administrative folder with restricted access.

The absolutely-essential requirement of rapid application development is programming software that will expedite the build process. HTML editors like FrontPage, Dreamweaver UltraDev, and others can serve but do require some rudimentary knowledge of HTML and ASP.

FrontPage's tool for creating ASPs is its Database Results Wizard. Most development is done in the "Normal" or WYSIWYG ("What You See Is What You Get") view of FrontPage.

#### To Create a Page to Add a Record to the Database

- Insert a form (Figure 2, in Multimedia Appendix 1).
- Insert a table into the form (Figure 3, in Multimedia Appendix 1).
- Create a name for each form field, the data field, and, if a field is validated, an error-message name for the data field (Figure 4 and Figure 5, in Multimedia Appendix 1).
- Right-click in the form to access the form properties (Figure 6, in Multimedia Appendix 1)
- Assign a name for the form field (Figure 7, in Multimedia Appendix 1)
- Send the Web page data fields to a selected database and table (Figure 8 and Figure 9, in Multimedia Appendix 1)
- Enter the URL of a confirmation page (Figure 10, in Multimedia Appendix 1)
- Map the saved Web-page data fields to their corresponding database data fields (Figure 11, in Multimedia Appendix 1).

#### Two Active Server Pages are Needed to Update a Record

**First Page**

In the first page, insert a database, which will evoke the Database Results Wizard (DRW) (Figure 12, in Multimedia Appendix 1).

The Database Results Wizard has 5 steps:

- Step 1 defines the database connection (Figure 13, in Multimedia Appendix 1).
- Step 2 selects the table or predefined query in the database (Figure 14, in Multimedia Appendix 1).
- Step 3 defines the data fields to be displayed, the selection criteria, the order in which the results are to be displayed, default values, the limit of records to be displayed, and an error message if no records that meet the selection criteria are found (Figure 15-Figure 17, in Multimedia Appendix 1).
- In step 4, select "List - one field per item" and "Table" as the list option (Figure 18, in Multimedia Appendix 1).
- In step 5, select "display all records together" (Figure 19, in Multimedia Appendix 1).

After finishing the Database Results Wizard:

- Select and cut the table, insert a form, and paste the table within the form (Figure 20 and Figure 21, in Multimedia Appendix 1).
- Add another column to provide input to modify the existing content (Figure 22, in Multimedia Appendix 1).
- The fields to be modified should have as their initial values "<%=FP_FieldVal(fp_rs,"Data field name")%>" (without the leading and trailing quotation marks) (Figure 23, in Multimedia Appendix 1).
- Remove the "reset" button and do not make the primary key data field updateable (Figure 24, in Multimedia Appendix 1).

**Second Page**

- In form properties post the form data to the second update page, ie, update2.asp, along with a primary key as the hidden value ("<%=Request("ID")%>") (without the leading and trailing quotation marks) (Figure 25-Figure 26, in Multimedia Appendix 1).
- For the second update page, invoke the Database Results Wizard (Figure 27, in Multimedia Appendix 1). Select "Custom Query" (Figure 28, in Multimedia Appendix 1) and "Edit" to enter the SQL (Structured Query Language) expression (Figure 29, in Multimedia Appendix 1): 
                            
UPDATE Database.Table 
SET Databasefield1='::URLformfield1::', 
Databasefield2='::URLformfield2::' 
. . . Databasefieldn='::URLformfieldn::', 
WHERE Primary key in database=::Primary key in database in URL form field::

- In step 3, select "More options" and enter a confirmation message (Figure 30, in Multimedia Appendix 1)
- Select "Next" in step 4 and "Finish" in step 5. Save the page as "update2.asp" (Figure 31, in Multimedia Appendix 1)
- If desired, a customized confirmation page can be created (Figure 32-Figure 37, in Multimedia Appendix 1). The page can be saved as "delete1.asp"
- The second delete page is created by the Database Results Wizard. In Step 2 (Figure 38-Figure 40, in Multimedia Appendix 1), select "Custom Query" and "Edit" to enter: 
                            
DELETE * FROM Results 
WHERE (Primary key in database = ::Primary key in database in URL form field::)

- In Step 3, "More Options," add a message confirming record deletion, eg, "Record deleted" (Figure 41, in Multimedia Appendix 1). Select "Next" for Step 4. In Step 5, select "Display all records together" and ensure that the "Add search form" is not selected (Figure 41, in Multimedia Appendix 1). To send the end user back to the application's home page (or elsewhere), add the following line to the <HEAD> and </HEAD> tags of the delete page in the HTML view: <meta http-equiv="refresh" content="1;URL=Application home page">.

#### Advice

Use either Notepad or Access Query Builder to compose SQL statements. SQL copied from other word processing programs, like Microsoft Word, or copied directly from the Web can contain characters that are incompatible with SQL. For example, a perpendicular single quote (') is a reserved character in SQL that delimits a literal in Microsoft Access' version of SQL. In contrast, a slanted single quote or apostrophe (`) is not SQL compliant and will generate an error message. Pasting either into Notepad will prevent the error.

Filtered results can be generated with SQL in step 2 of the Database Results Wizard. The remaining steps in the Database Results Wizard define selection criteria, result order, and result format. A results page can generate reports in read-only views for the general end-user or serve as the portal for interactive, read-write administrative access. Hyperlinks with the appropriate parameters, like the primary-key database field, can direct the user to the update or delete pages, or a details view for individual records within a recordset.

As with any application, working code means little without sufficient attention to usability. To minimize needless duplicate-data entry, confirmation pages should be built to verify successful record entry and intentional deletion of a selected record. Drop-down menus for database queries and pages that add a new record to the database speed data entry.

Navigation and font types and sizes should adhere to recognized usability guidelines. The National Institute on Aging and the National Library of Medicine provide a concise set of guidelines in pamphlet format [9]. Jakob Nielsen is a leading authority on Web usability and has authored 2 volumes on this topic [10,11]. Font size should be no smaller than 12 point. Navigation should always provide an escape to the end user's page of entry. Buttons for frequently-hit pages improve hyperlink visibility. Customized confirmation pages for adding a record, and confirm messages or pages prior to deleting a record are built as needed.

If new Windows NT logon accounts are created or a preexisting end-user logon has never been used before, provide training via phone or e-mail about logging on to restricted sub-Webs, ie, user-name conventions, case-sensitive passwords, and specific domains (if there is more than one domain). Forewarn your information-systems help desk of the new application and possible calls about logon issues.

Since we use Microsoft products enterprise wide, Microsoft Access was chosen as our database for DDWA. Any other database program should serve in other environments. Though blessed with a shorter learning curve and greater accessibility than its big-database siblings, Access does have acknowledged limitations. Though the documentation states that the size of the Access *.mdb file (where * represents file name and *mdb* indicates an Access file) can reach 2 gigabytes, anecdotal reports posted on the Internet suggest a working file size of 50000 records. The documentation suggests that the maximum number of users is 250. Practical experience suggests that no more than 15 to 20 concurrent users can use the application at any given time. However, a session via HTTP may only last seconds. Even if a given Web page is on a user's Web browser an entire day, the time actually spent calling that database is only seconds.

That said, if an Access database becomes corrupted or Internet Information Server (IIS) becomes unstable, moving to a more robust database, like SQL Server or Oracle, should be considered [12,13].

## Discussion

Just-in-time database-driven Web applications are inexpensive, quickly-developed software that can be put to many uses within a health care organization regardless of its size.

### Saint Vincent Catholic Medical Centers' DDWA

Although DDWA only constituted 5 percent (73873/1505865) of all intranet hits in 2002, they enabled collaboration and communication via user-friendly Web browser-based interfaces for both mission-critical and patient care-functions (Figure 42-Figure 73, in Multimedia Appendix 2). Screenshots of Saint Vincent Catholic Medical Centers-intranet home pages for the major sub-Webs are shown in Figure 74 to Figure 86, in Multimedia Appendix 2.

For the 19 DDWA in production mode in 2002, the benefits accrued included:

- improved continuity of care within the system from other settings of care to behavioral-health care and long-term care (reducing "leakage" from the integrated delivery system)
- transition of multiple financial systems to one system
- sign-out patient rosters, resident evaluations, and clinical-research opportunities in graduate medical education
- online scheduling for instructor-led classes and online testing for self-learning programs
- managed care-related announcements system wide without the time and expense associated with paper-based memoranda
- a system-wide formulary, a composite of different formularies from 3 service divisions
- updateable phone directories
- online physician-credentials checking
- online reporting to expedite access to patient results.

New just-in-time DDWA in 2003 include new applications that permit the pharmacy to post formulary changes and drug advisories; information systems to announce virus warnings and downtime notices; and an application to facilitate compliance with the new Joint Commission on Accreditation of Healthcare Organizations (JCAHO) Staffing Effectiveness standards.

### Benefits of DDWA

Just-in-time DDWA can be developed for little-to-no expense. The development cycle can be measured in hours to days. DDWA can distribute information and knowledge across an enterprise intranet and eliminate the need to map shared directories on multiple workstations. Read and read-write interaction with a database does not require facility with database software. The rapid turn-around time from conception to implementation generates high end-user satisfaction, as suggested by what some in our organization call DDWA — "low tech gimmes." Customized DDWA often fulfill niche needs that are too small and/or too temporary to warrant the cost of purchasing a shrink-wrapped application.

DDWA are disposable software, built for a targeted purpose for a prescribed time. For example, the suite of finance-related DDWA was built to be a temporary "crosswalk" from legacy applications in 3 regions to 1 new, system-wide finance system (Lawson). The HIV-viral-load application provides intranet-based patient-results reporting while implementing the new system-wide laboratory software (Softlab). The physician-credentialing application to a static database anticipates a live data connection to the new system-wide physician credentialing software (Morrissey). The online formulary precedes the implementation of a system-wide pharmacy system (Cerner). The job-description performance-appraisal application ceased once the standardization process neared completion.

However, the life span of a "temporary" DDWA may be months or may even span years. In contrast to many shrink-wrapped software products, unscheduled extensions of a DDWA do not generate additional license and maintenance fees.

DDWA can serve as a quick-and-dirty means to test a proof of concept. Whereas online testing for self-assessment programs proved popular and successful for staff training in Saint Vincent Catholic Medical Centers' behavioral-health product line, online continuing medical education for physicians about anthrax was less than successful. This trial continuing medical education (CME) project was conducted without incurring the cost associated with purchasing an online continuing medical education service or CD-based product.

DDWA can be endlessly iterative. Look-and-feel can easily be manipulated with static HTML. Code can be repositioned or labeled to promote more-commonly-used or general-access functions and demote administrative or restricted functions. Most requests for reports can be met with the proper SQL queries.

### Development of DDWA

Design should incorporate reusable components as much as possible. Directory structure should include, if needed, a separate folder with restricted access for administration. The essential database functions (add, delete, update, and query) apply to each application. The SQL syntax for delete and update, and queries (SQL select statements with where clauses) are the same for each application.

Testing is essential prior to general release of the application. The application should not be released until all discovered bugs have been rectified. The final version of the application should generate no error messages. Therefore, the more-complicated DDWA require prolonged testing. The bigger the project, the longer the development cycle. The resident-evaluation application remained untested and dormant until a medical student was assigned the task of testing the application. A project sponsor may be an early adopter but have no time to devote to the sometimes seemingly-perpetual cycle of testing and debugging. For example, it took multiple experiments with code over 2 weeks to finally determine that that the "NULL" or empty string values in SQL statements did not work predictably in creating a routing process for the resident evaluations.

As with larger software projects, if a DDWA loses its project sponsor the application may never finish the development cycle and may never be released. Since DDWA are only built upon request, the loss of the individual who drives a project may signal the demise of the application's further development. Project sponsors are often early adopters who, compared to their peers, better understand how information technology can promote more-efficient workflows or improve patient care. Unless a like-minded individual steps to the fore, the project will most likely cease prematurely.

As with any information technology project, scope creep can be a concern. Nevertheless, the very-transient nature of the just-in-time DDWA limits their scope. Applications that serve as temporary solutions to full-scale clinical or financial systems have circumscribed life spans. Purchasing a shrink-wrapped product should be reconsidered if an in-house application no longer meets growing requirements. The buy-versus-build decision can always be revisited.

Rapid application development presupposes the use of software to expedite coding. Although Web-development software like FrontPage and Dreamweaver Ultradev can facilitate DDWA, they do require some knowledge of HTML and ASP. The needed VBscript and Active Server Page resources, the requisite workarounds, and SQL can be found on the Web [14- 16]. Online forums and tutorials are also available, but assume both interest and time on the part of the novice developer [16- 20]. Given the resources, out-sourcing DDWA development to a vendor or purchasing specialized, shrink-wrapped DDWA may be easier albeit more expensive alternatives to in-house-developed DDWA. The decision is yet another shade of the classic dilemma of buy or build. If the wherewithal is generously provided, the former option usually prevails. However, given the current financial challenges faced by many not-for-profit health care organizations, buying may not always be an option.

### Limitations of DDWA

Just-in-time database-driven Web applications do have limitations. Just-in-time, on-the-fly DDWA cannot supplant complex applications like the electronic medical record. Although the sign-out roster had 455 patients in its database at the end of 2002, no more than 12 residents out of more than 270 internal medicine residents throughout the system utilized the application. The absence of interfaces to pharmacy, laboratory, and the admissions-discharge-transfer systems (ADT) make manual key entry of patient data prohibitive. Though useful, templates and data-field validation alone cannot replace full-featured clinical decision support. Nevertheless, intranet-based, secure DDWA with personal health information do protect patient privacy and confidentiality in a HIPAA (Health Insurance Portability and Accountability Act)-compliant manner and offer a waypoint on the path to the computer-based patient record.

Development on-the-fly (ie, with a degree of haste and improvisation) can easily generate typographic errors and a host of bugs. Internet Explorer will identify the Web page address (URL) of a page whose code is too faulty to process. Error messages from Internet Explorer rarely identify a misspelled data-field name or even the correct line of code where the error resides. Debugging an Active Server Page with hundreds of lines can be daunting for a professional but even more so for the novice. Wizards like FrontPage's Database Results Wizard generate bloated code, with significant volumes of unnecessary lines that may prolong Web-page download times.

### Conclusions

With all its limitations, just-in-time database-driven Web applications can provide remarkable value for selected projects for little-to-no cost.

## Abbreviations

ASP: Active Server Page
DDWA: Database-Driven Web Application(s)
SQL: Structured Query Language

## Multimedia Appendix 1

Figure 1 - 41

## Multimedia Appendix 2

Figure 42 - 86
