# Supplementary material for: Just-in-time Database-Driven Web Applications
Source: J Med Internet Res. 2003 Aug 29;5(3):e18. doi: 10.2196/jmir.5.3.e18 (PMC1550565; doi:10.2196/jmir.5.3.e18)
Supplement: Supplementary file 1 [file jmir_v5i3e18_app1.ppt]

## Slide 1
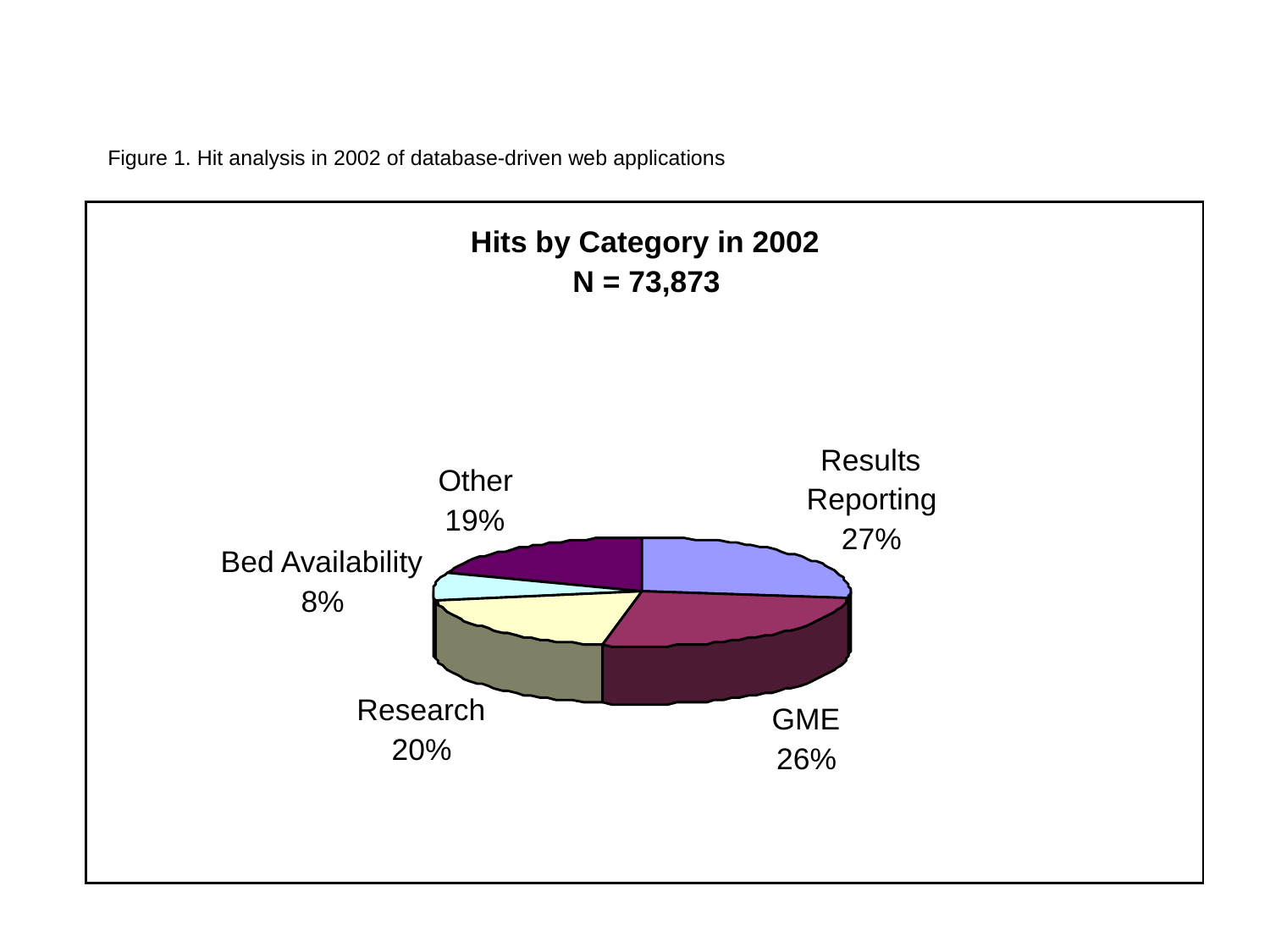

# Figure 1. Hit analysis in 2002 of database-driven web applications
Hits by Category in 2002
N = 73,873
Results
Other
Reporting
19%
27%
Bed Availability
8%
Research
GME
20%
26%

## Slide 2
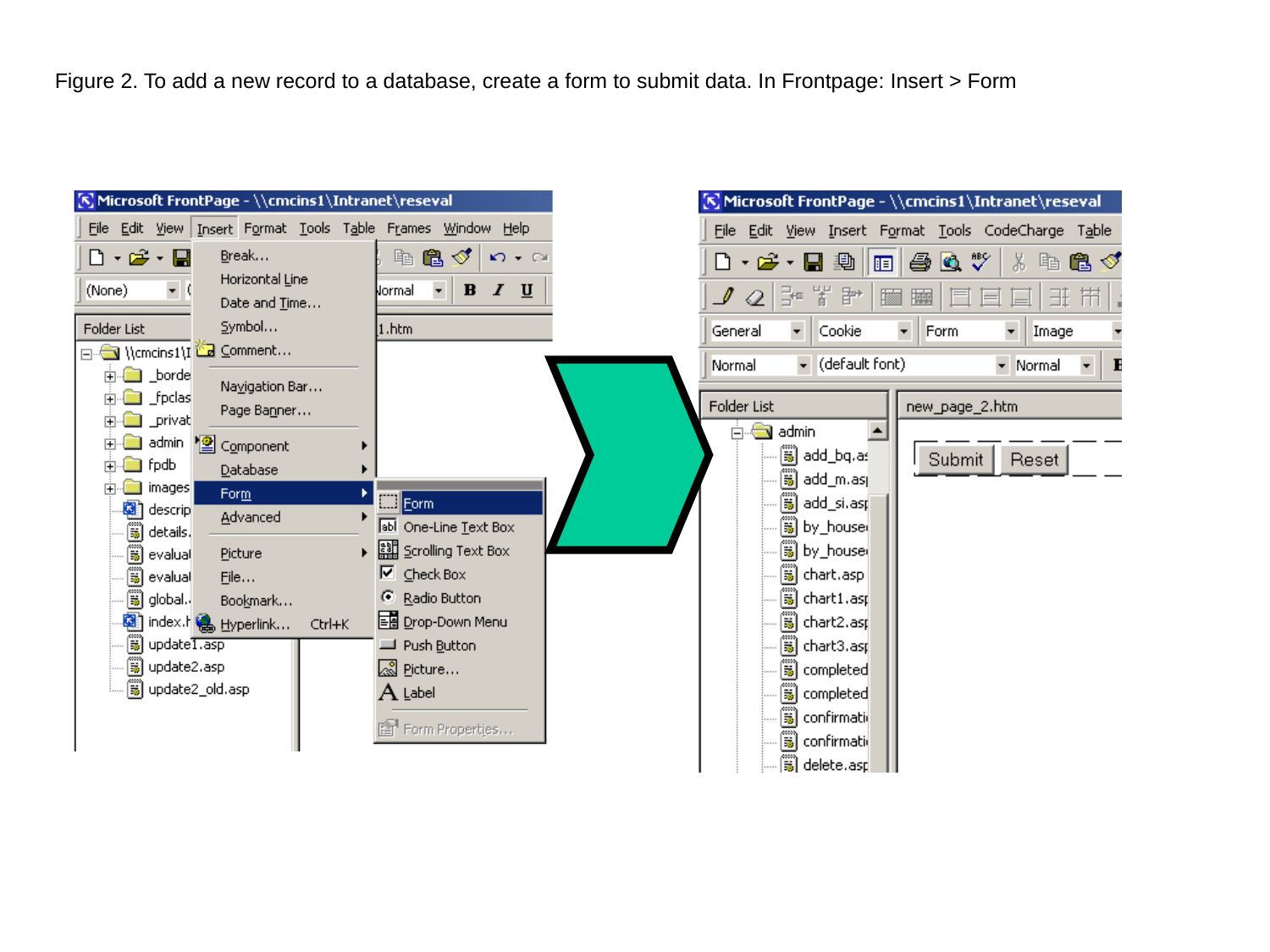

# Figure 2. To add a new record to a database, create a form to submit data. In Frontpage: Insert > Form

## Slide 3
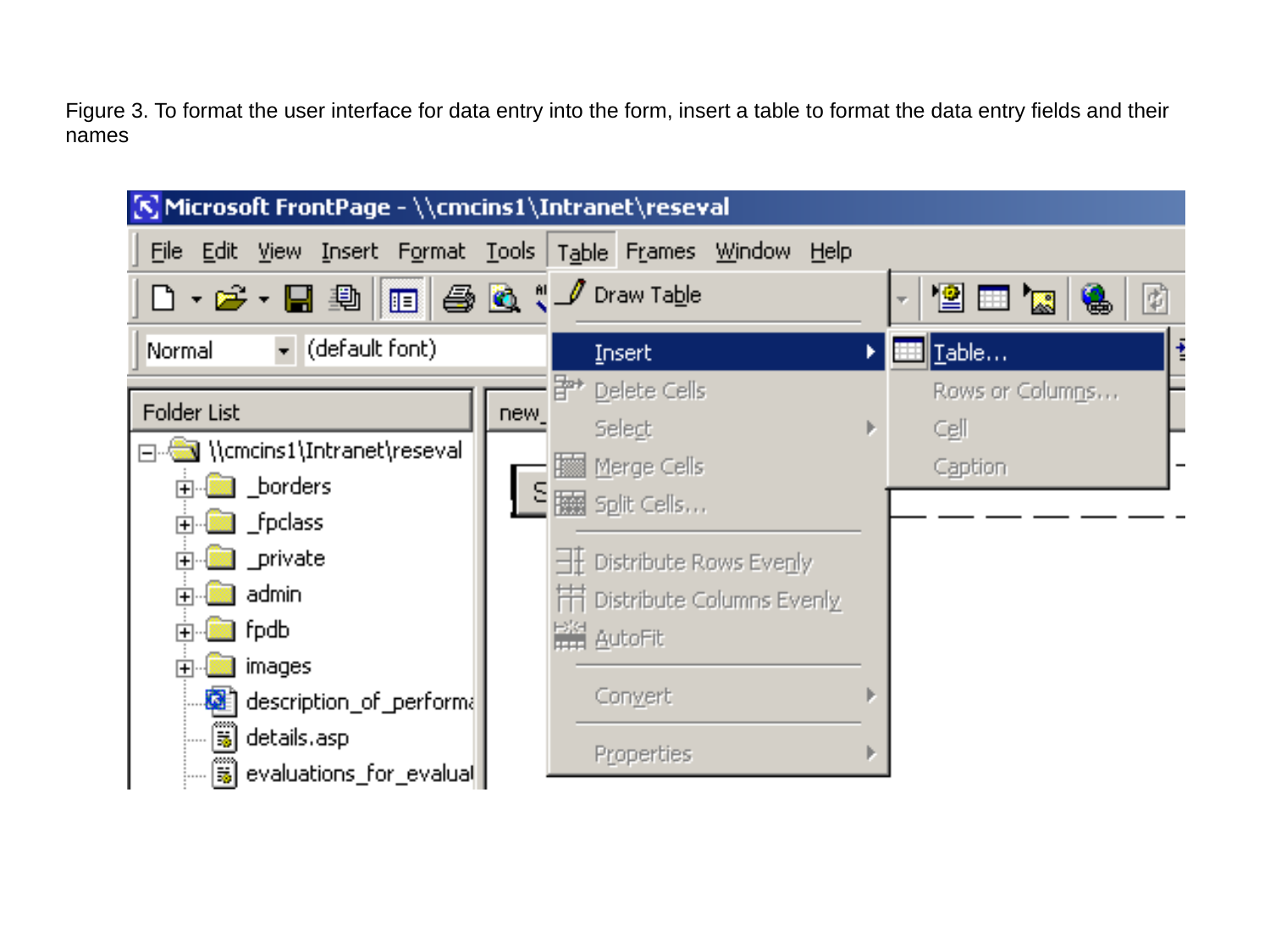

# Figure 3. To format the user interface for data entry into the form, insert a table to format the data entry fields and their names

## Slide 4
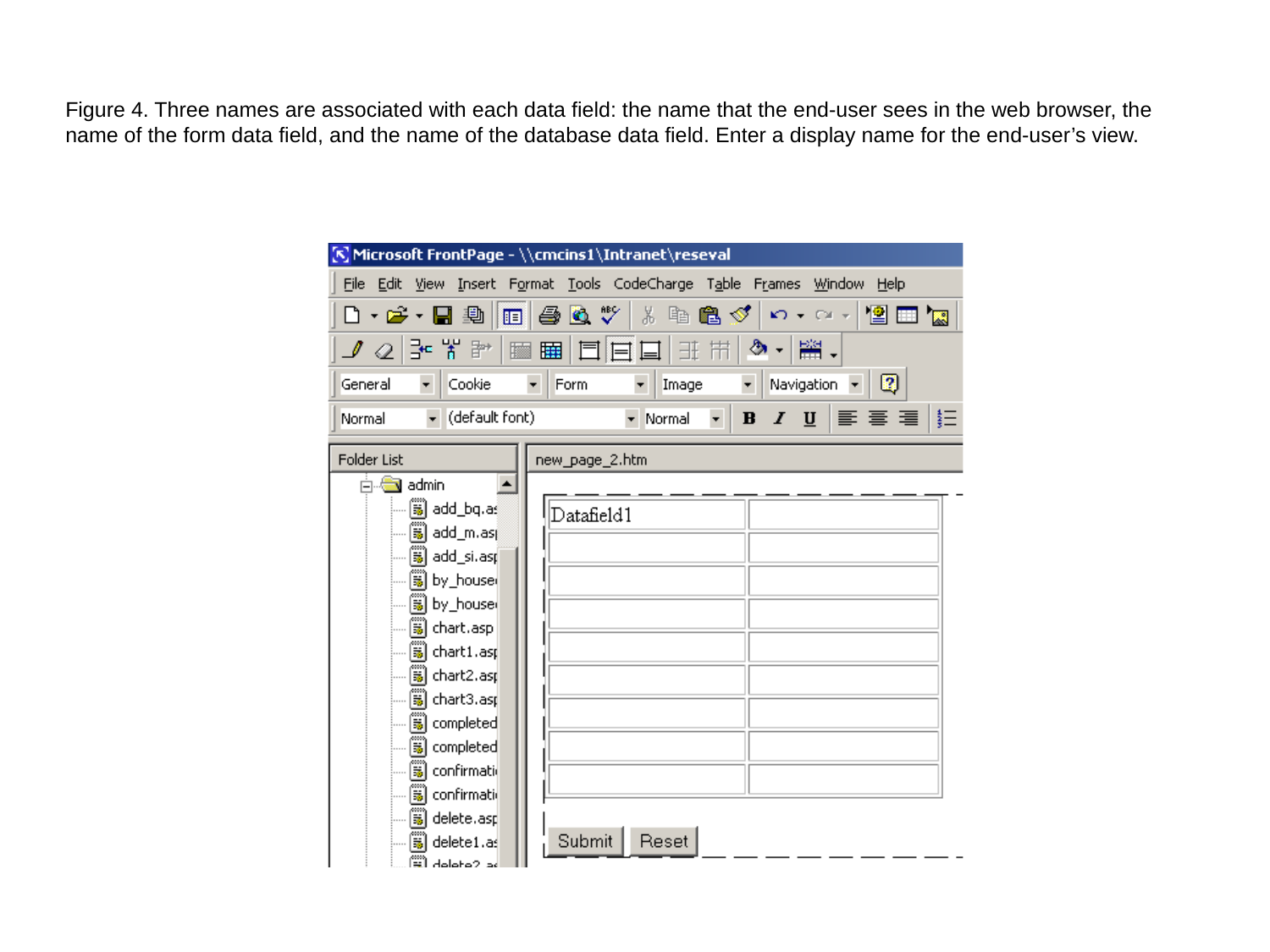

# Figure 4. Three names are associated with each data field: the name that the end-user sees in the web browser, the name of the form data field, and the name of the database data field. Enter a display name for the end-user’s view.

## Slide 5
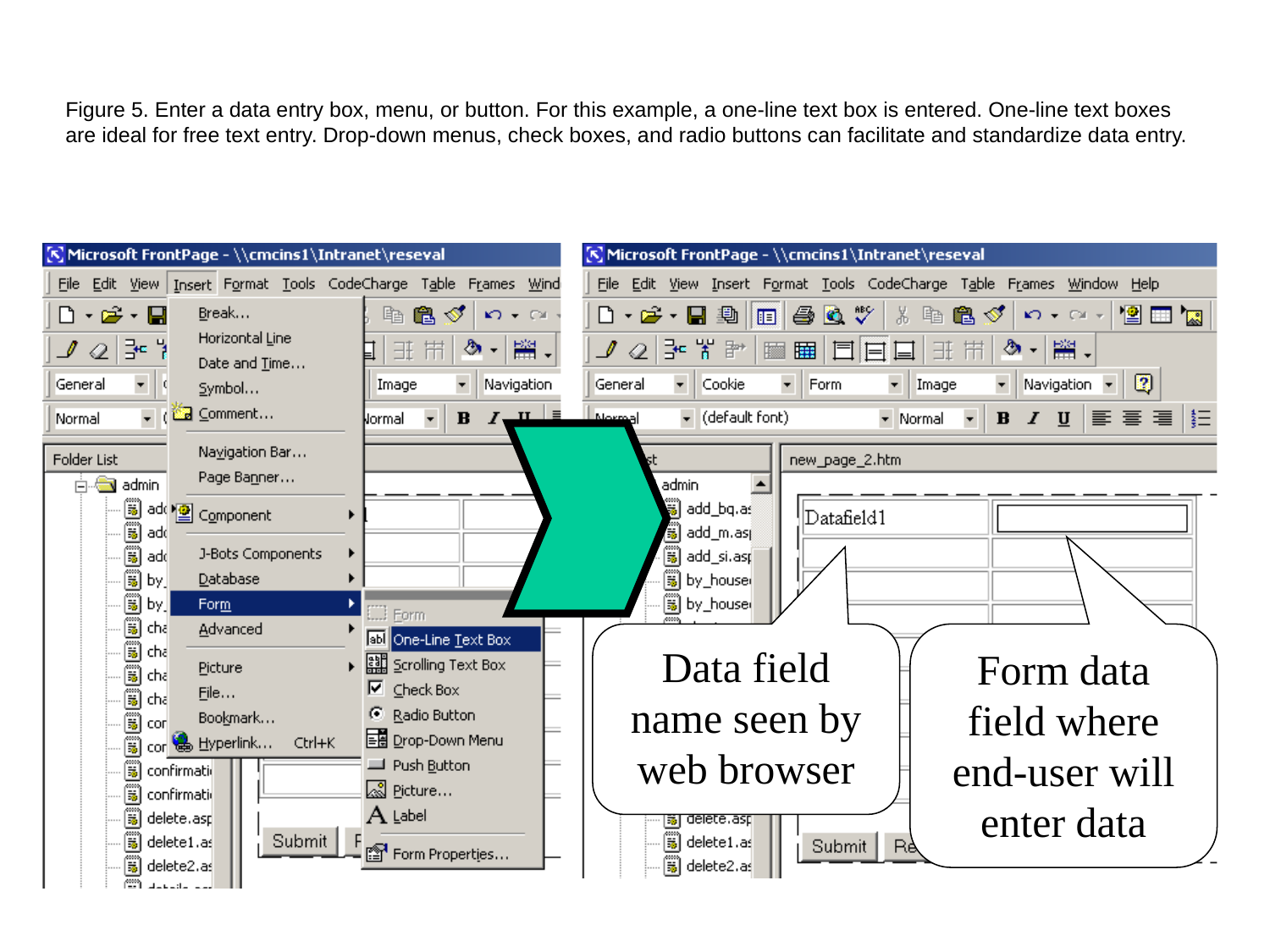

# Figure 5. Enter a data entry box, menu, or button. For this example, a one-line text box is entered. One-line text boxes are ideal for free text entry. Drop-down menus, check boxes, and radio buttons can facilitate and standardize data entry.
Data field name seen by web browser
Form data field where end-user will enter data

## Slide 6
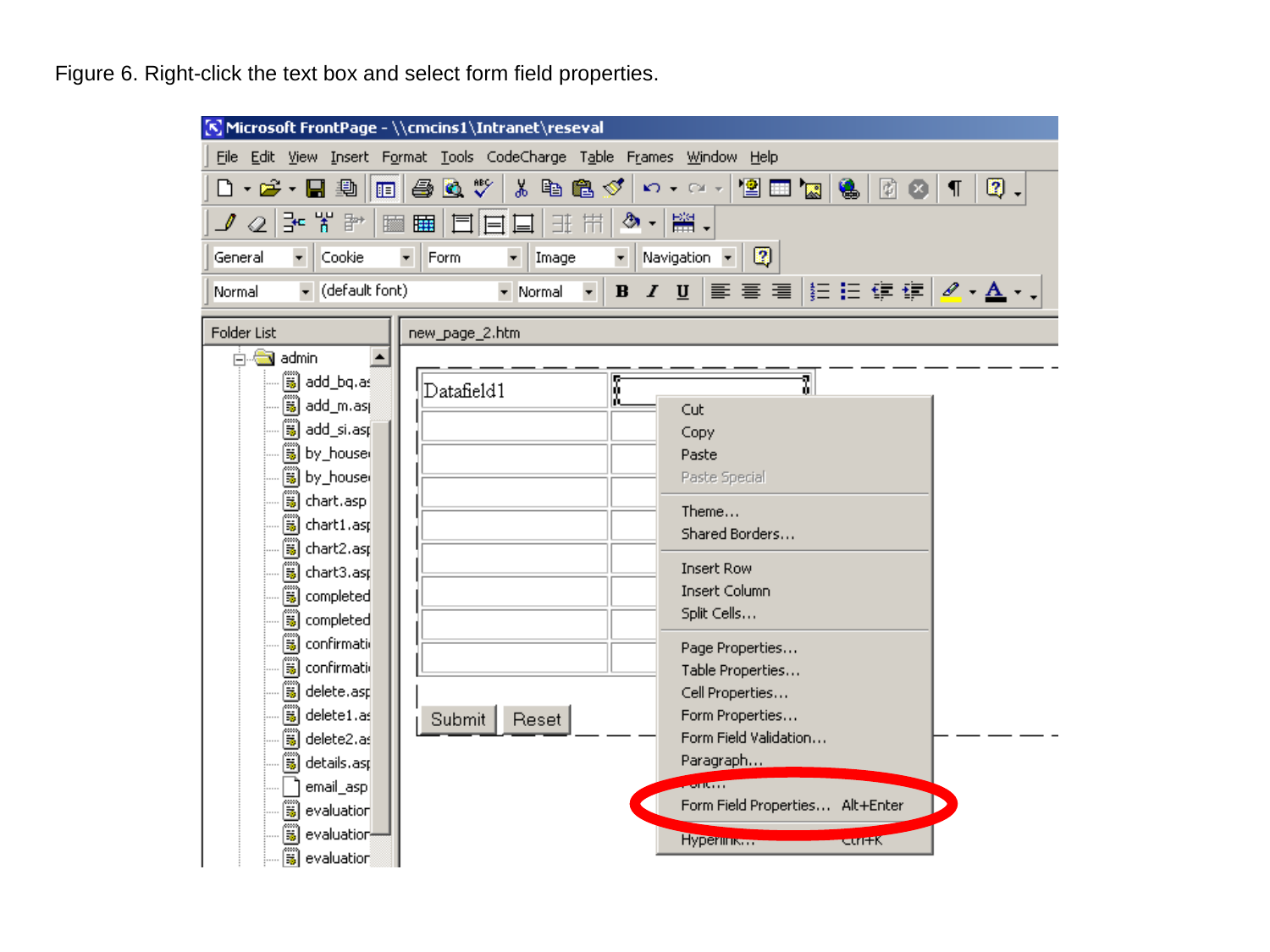

# Figure 6. Right-click the text box and select form field properties.

## Slide 7
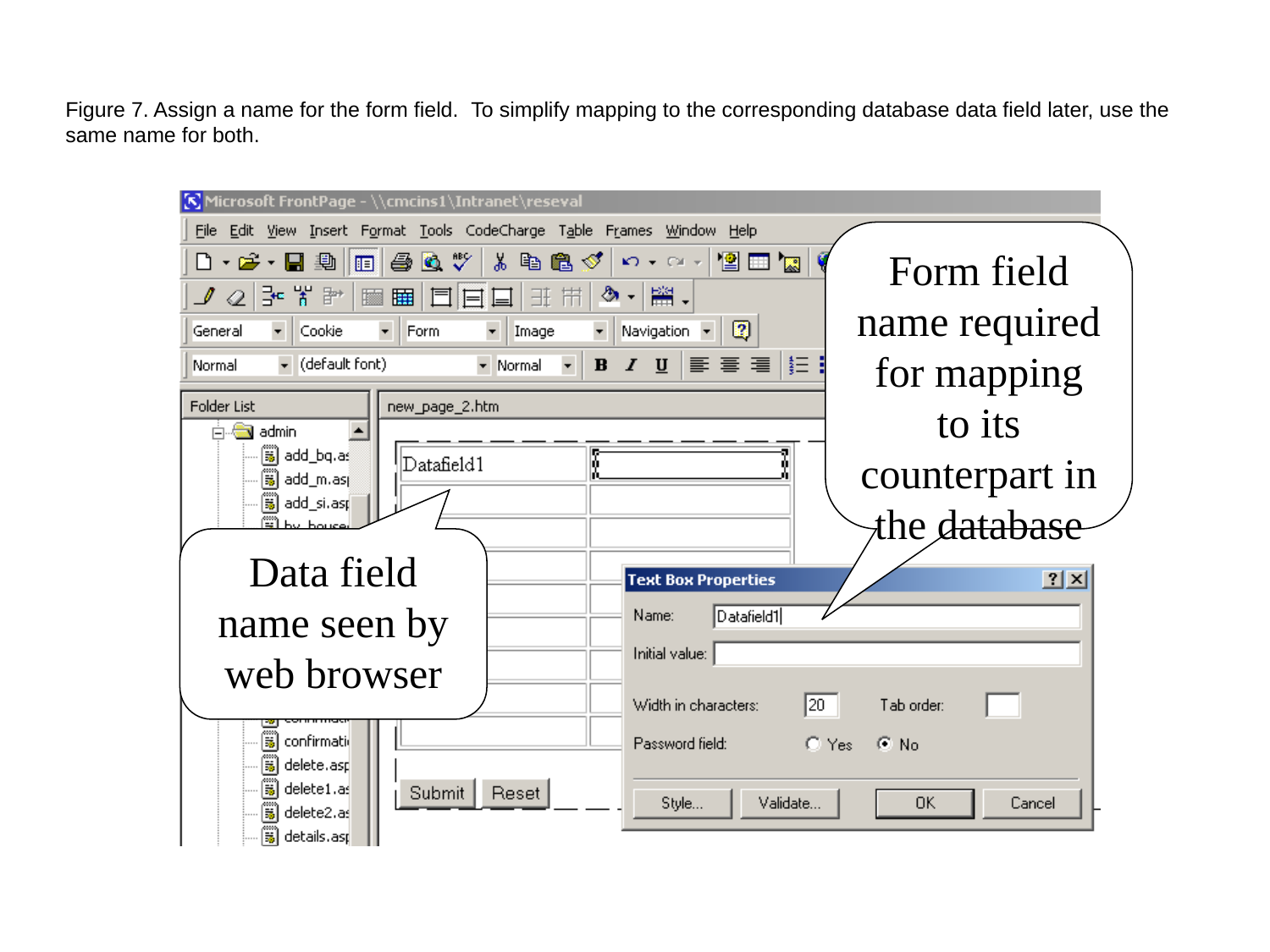

# Figure 7. Assign a name for the form field. To simplify mapping to the corresponding database data field later, use the same name for both.
Form field name required for mapping to its counterpart in the database
Data field name seen by web browser

## Slide 8
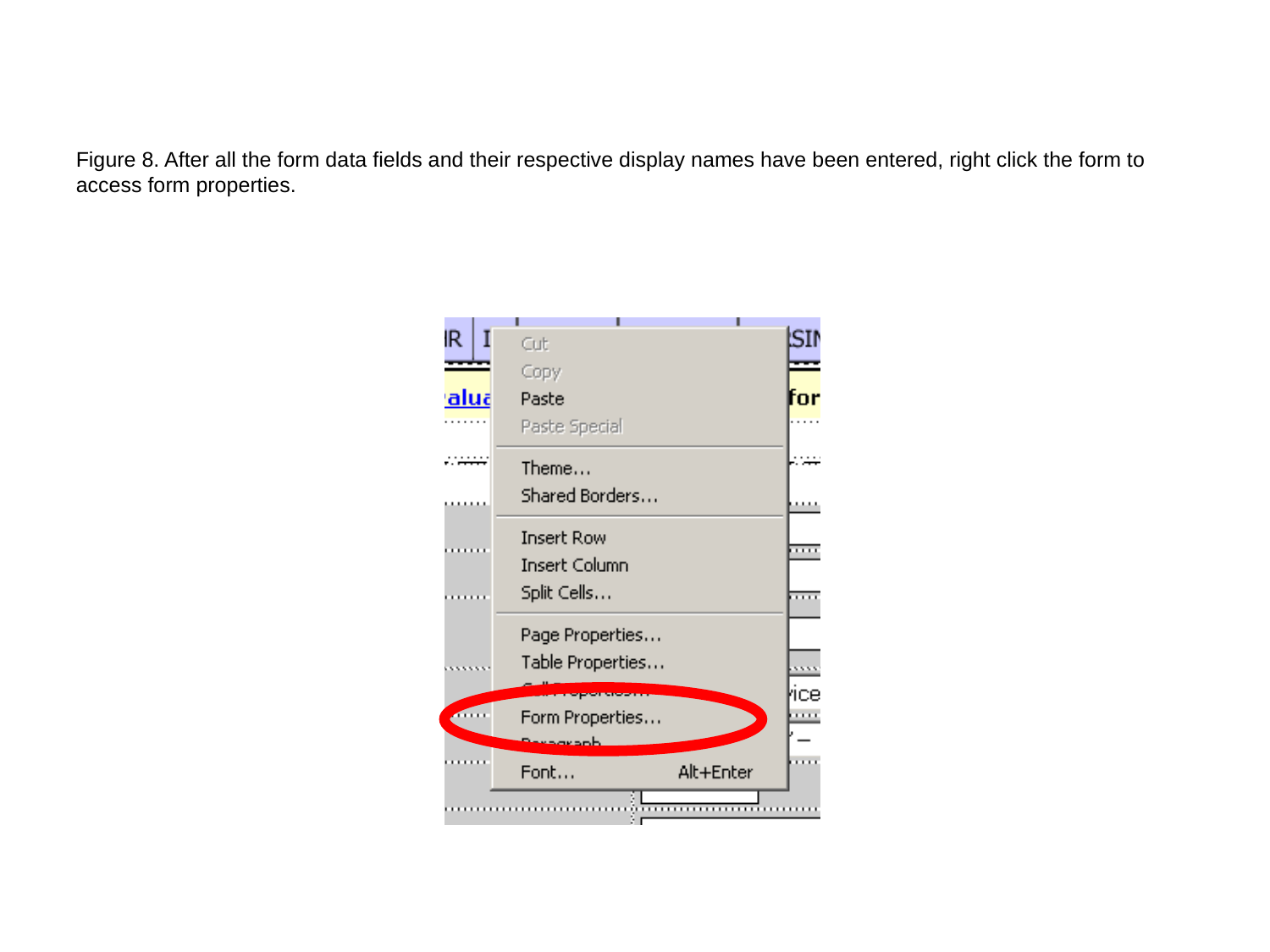

# Figure 8. After all the form data fields and their respective display names have been entered, right click the form to access form properties.

## Slide 9
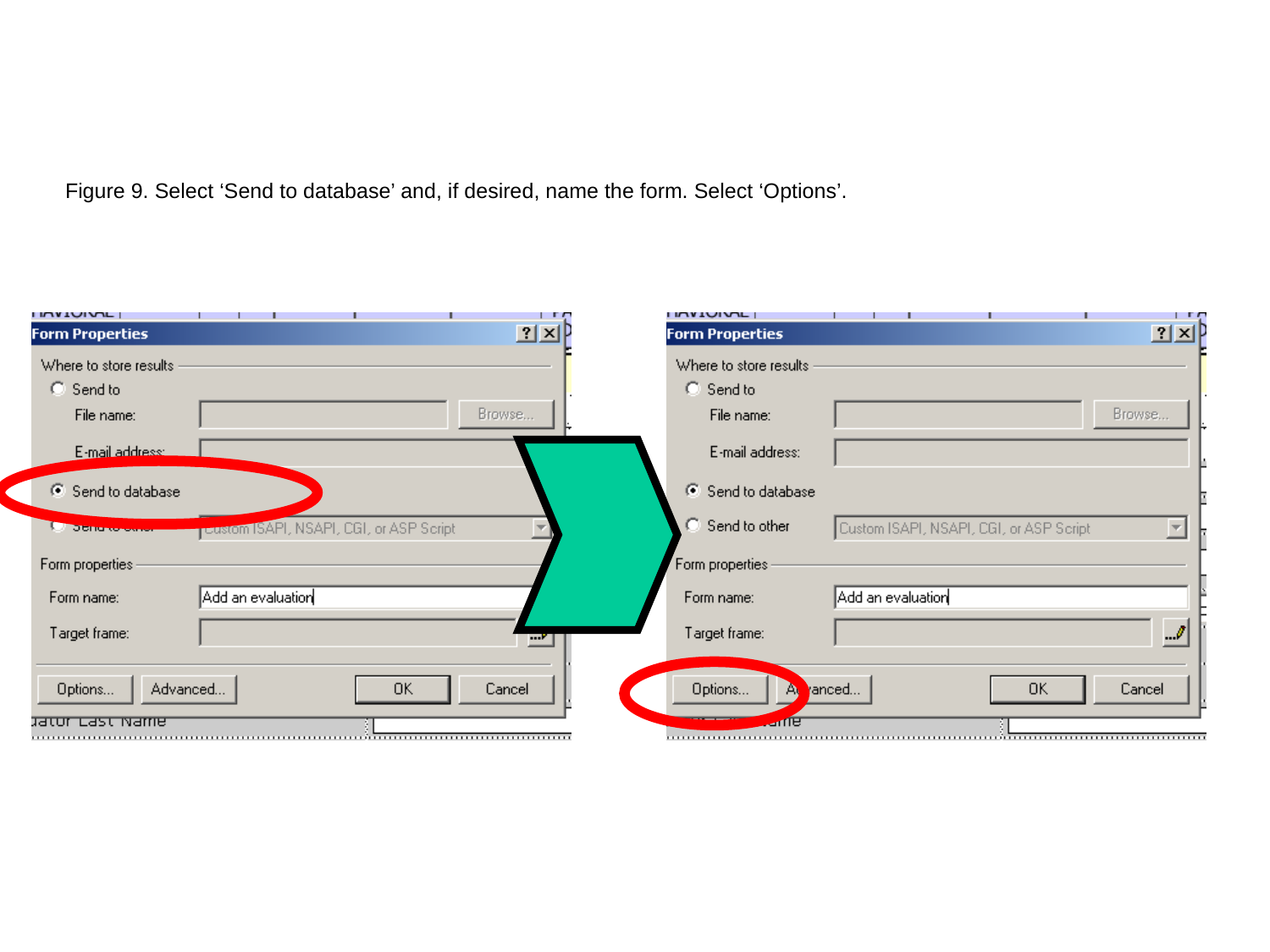

# Figure 9. Select ‘Send to database’ and, if desired, name the form. Select ‘Options’.

## Slide 10
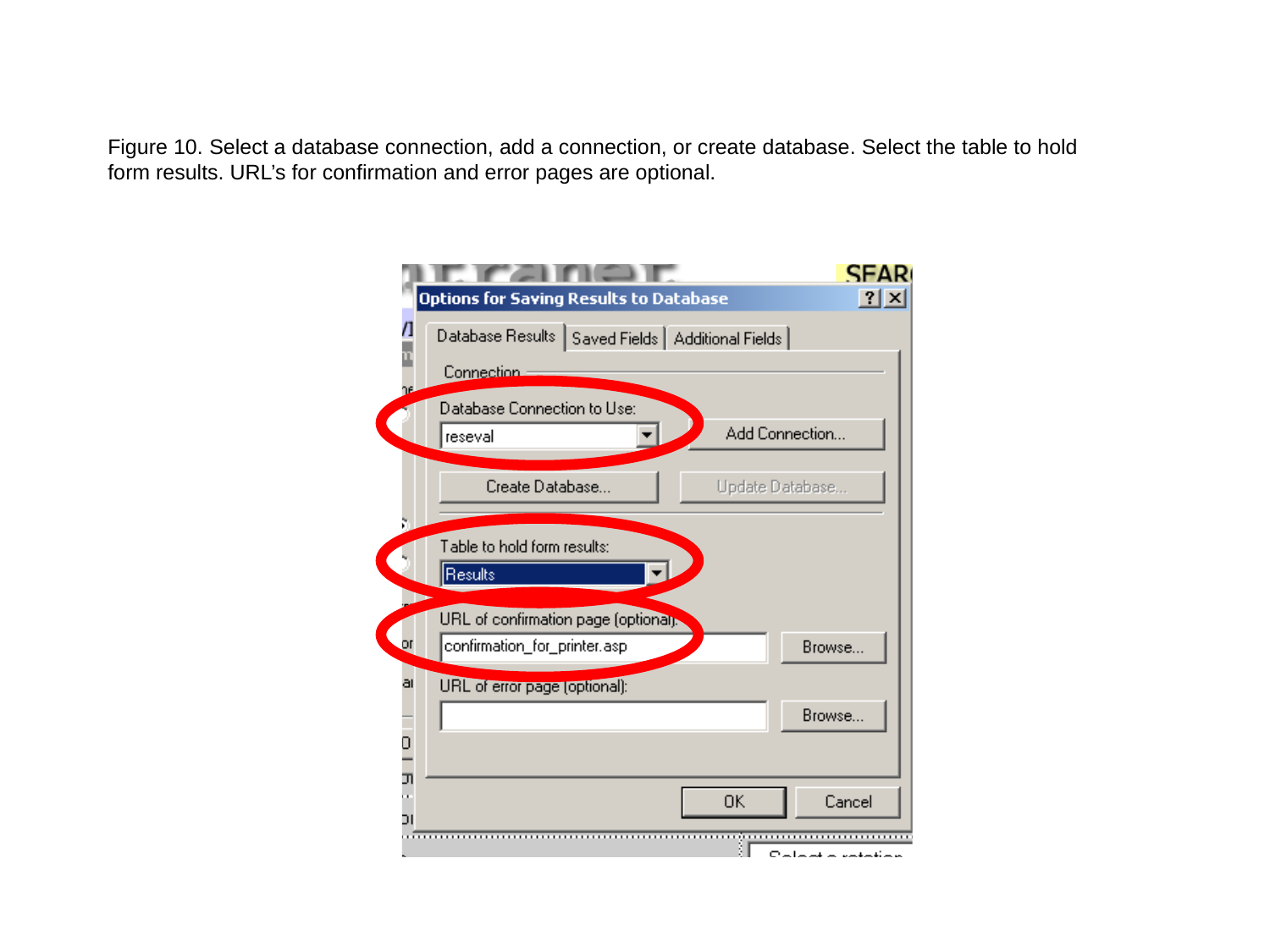

# Figure 10. Select a database connection, add a connection, or create database. Select the table to hold form results. URL’s for confirmation and error pages are optional.

## Slide 11
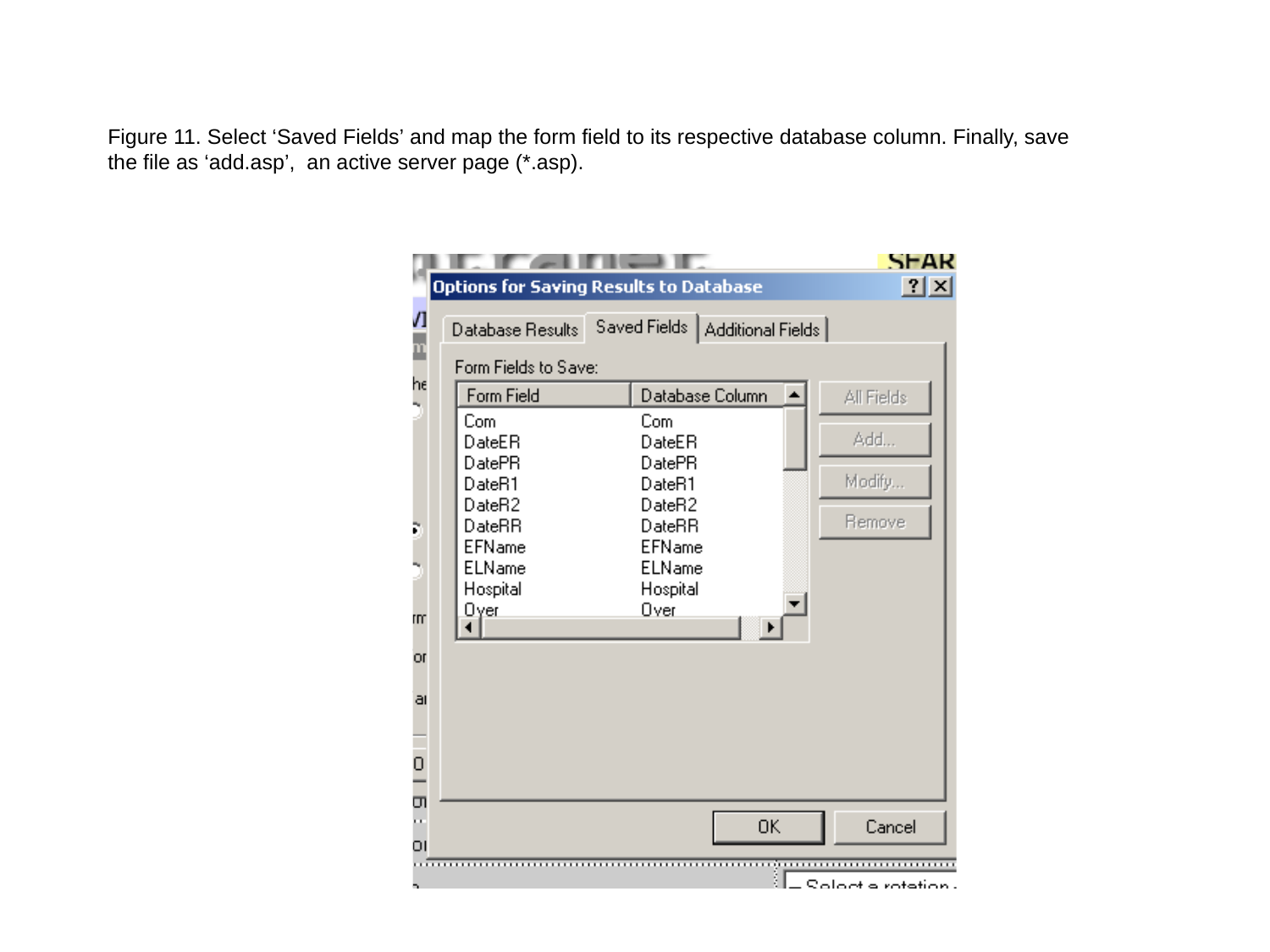

# Figure 11. Select ‘Saved Fields’ and map the form field to its respective database column. Finally, save the file as ‘add.asp’, an active server page (*.asp).

## Slide 12
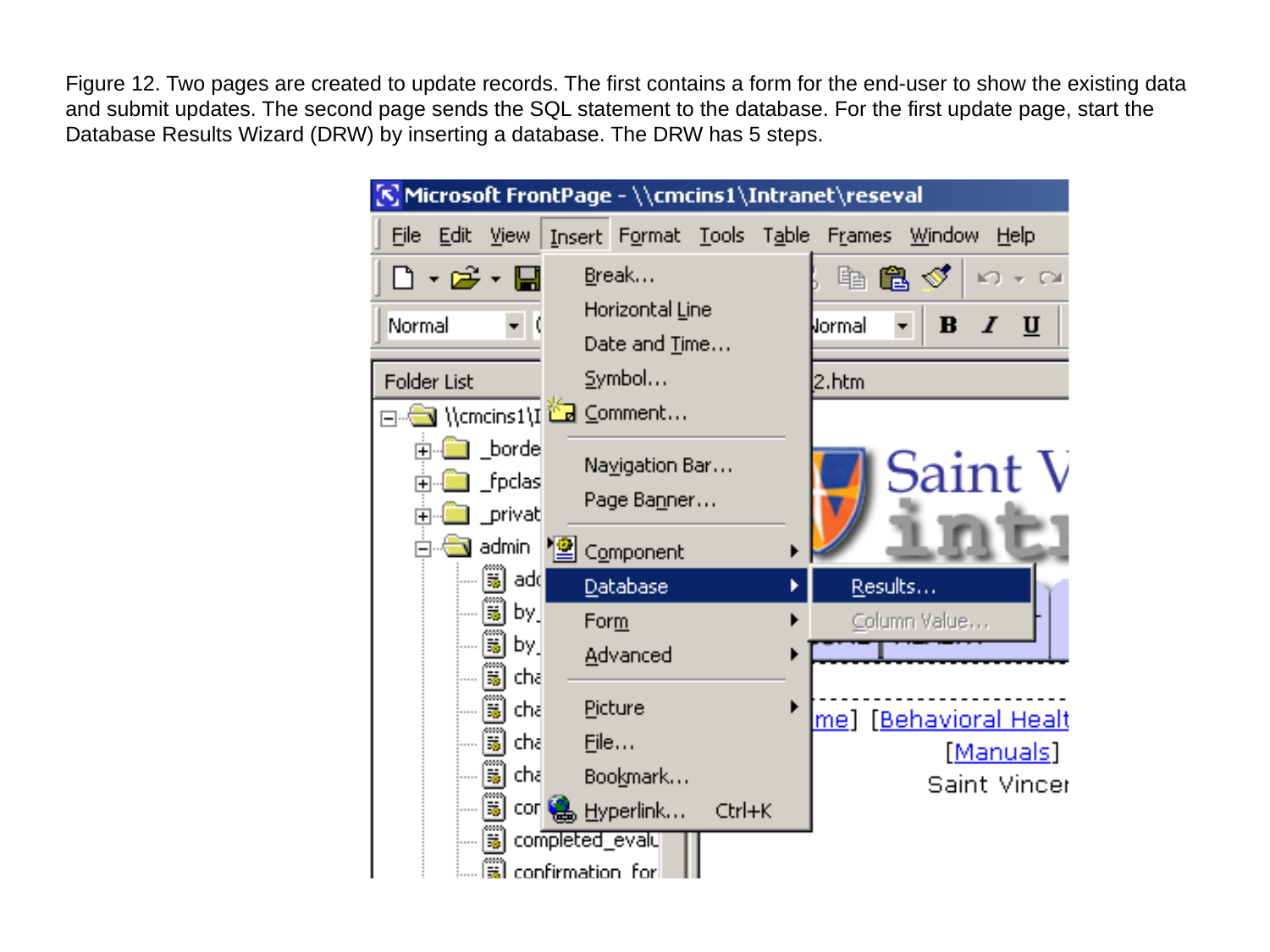

# Figure 12. Two pages are created to update records. The first contains a form for the end-user to show the existing data and submit updates. The second page sends the SQL statement to the database. For the first update page, start the Database Results Wizard (DRW) by inserting a database. The DRW has 5 steps.

## Slide 13
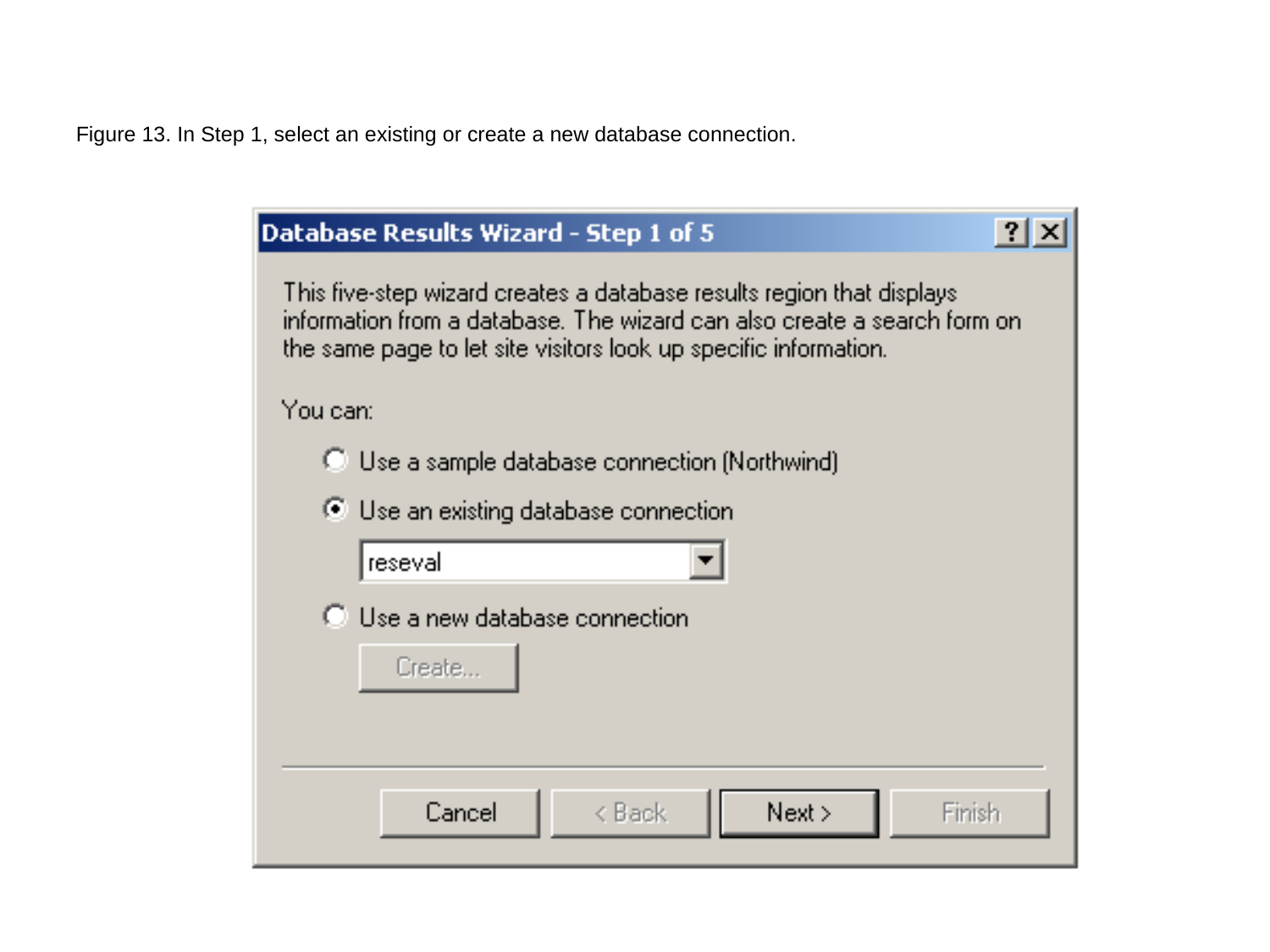

# Figure 13. In Step 1, select an existing or create a new database connection.

## Slide 14
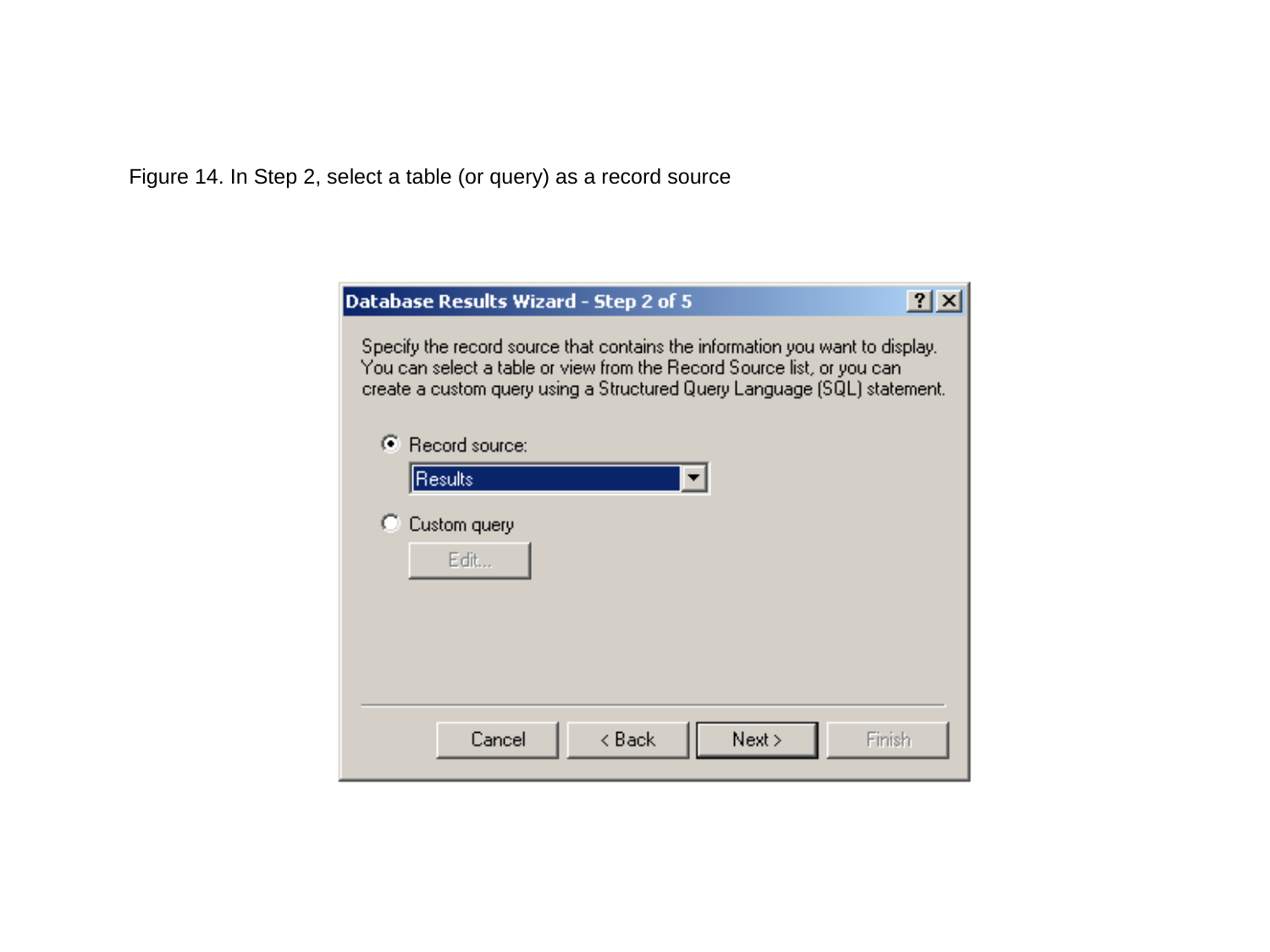

# Figure 14. In Step 2, select a table (or query) as a record source

## Slide 15
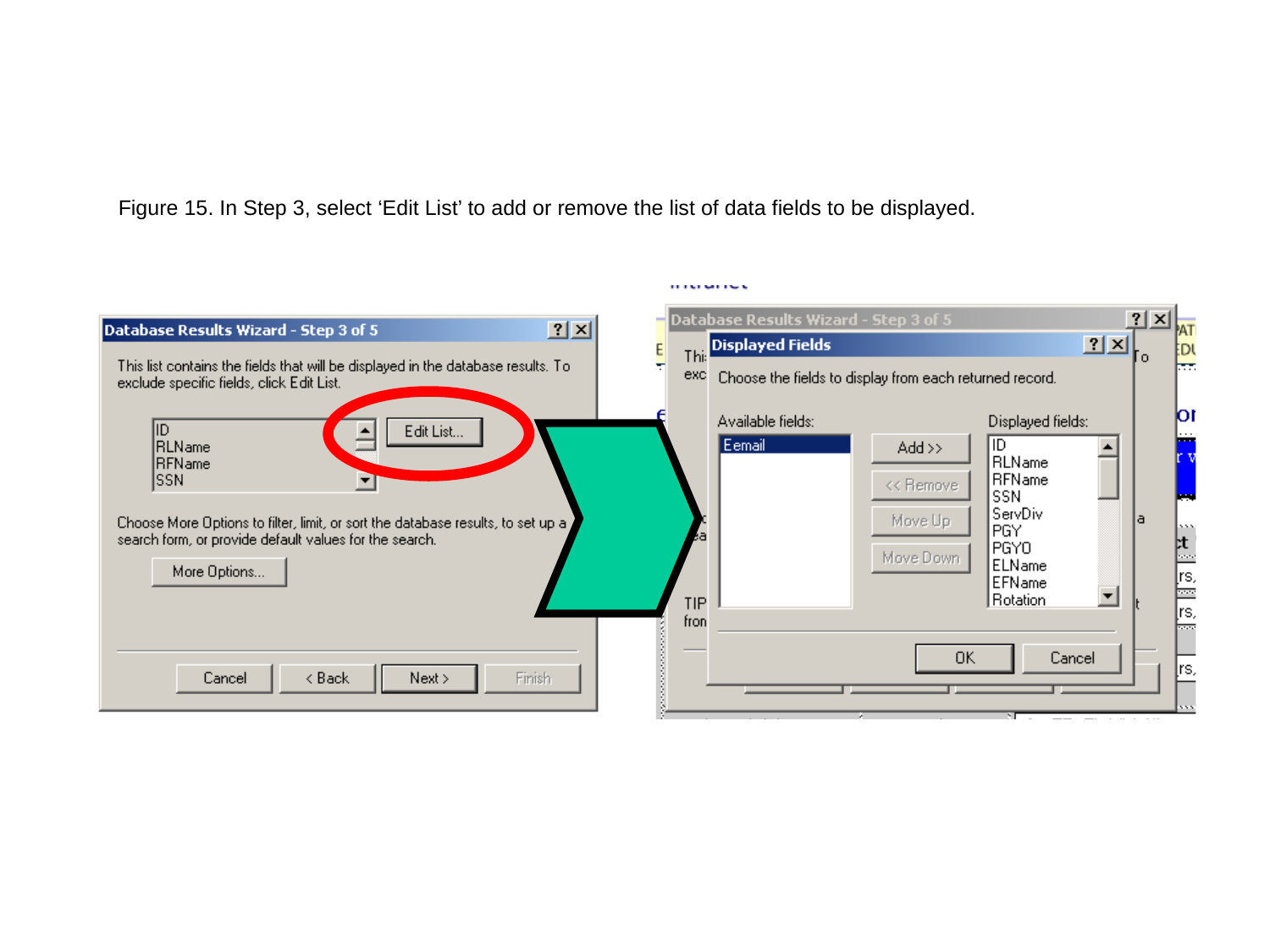

# Figure 15. In Step 3, select ‘Edit List’ to add or remove the list of data fields to be displayed.

## Slide 16
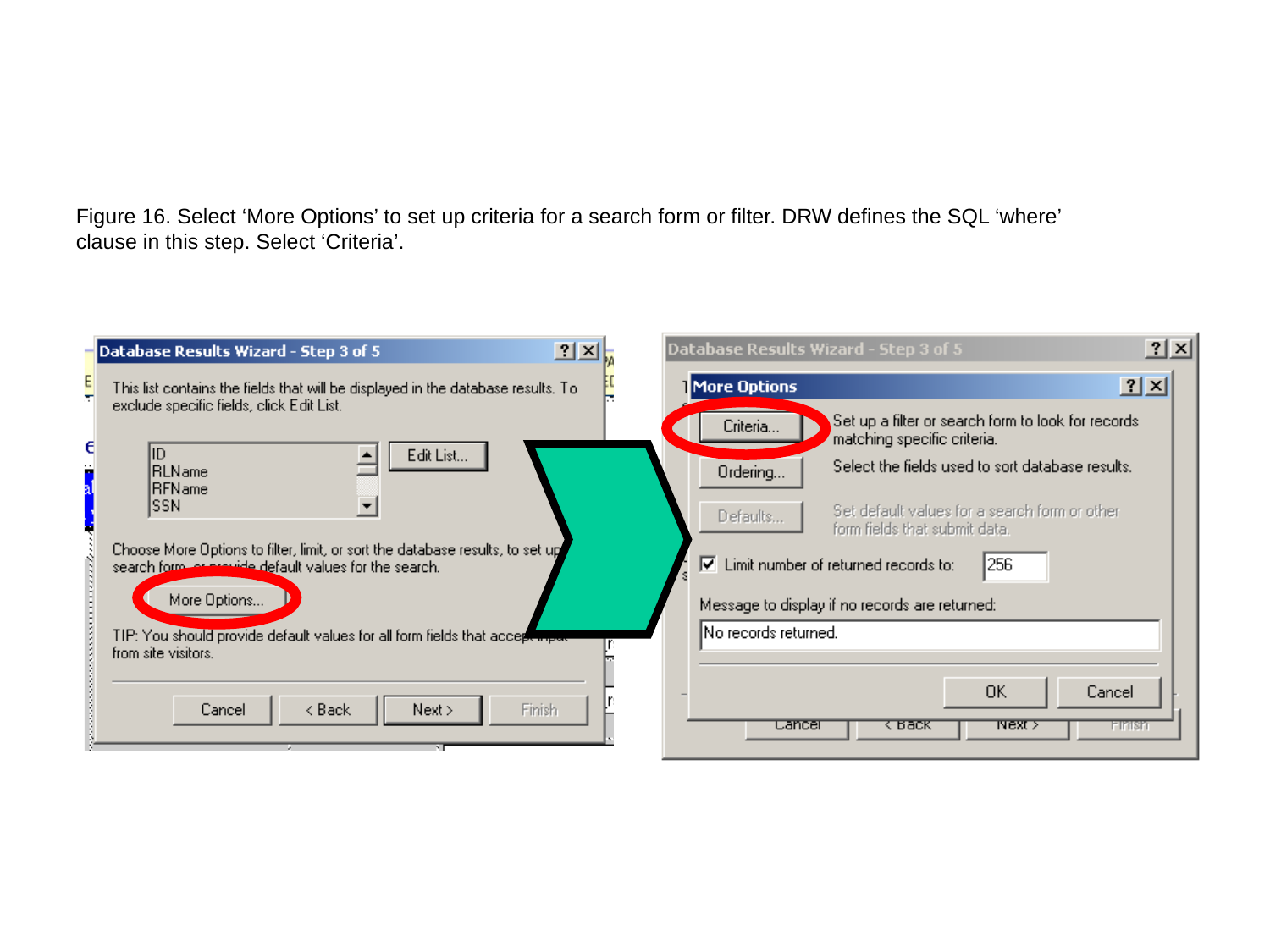

# Figure 16. Select ‘More Options’ to set up criteria for a search form or filter. DRW defines the SQL ‘where’ clause in this step. Select ‘Criteria’.

## Slide 17
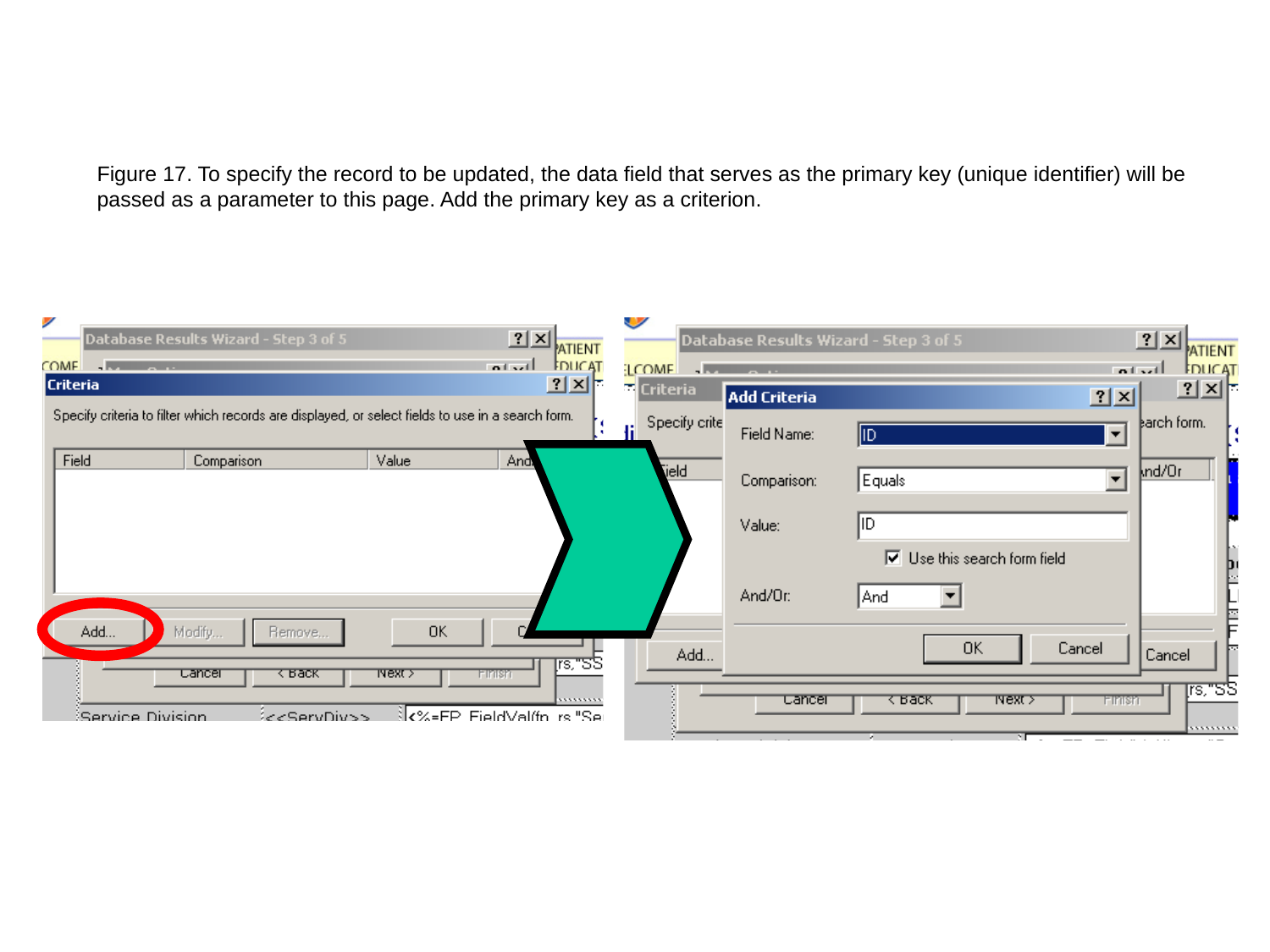

# Figure 17. To specify the record to be updated, the data field that serves as the primary key (unique identifier) will be passed as a parameter to this page. Add the primary key as a criterion.

## Slide 18
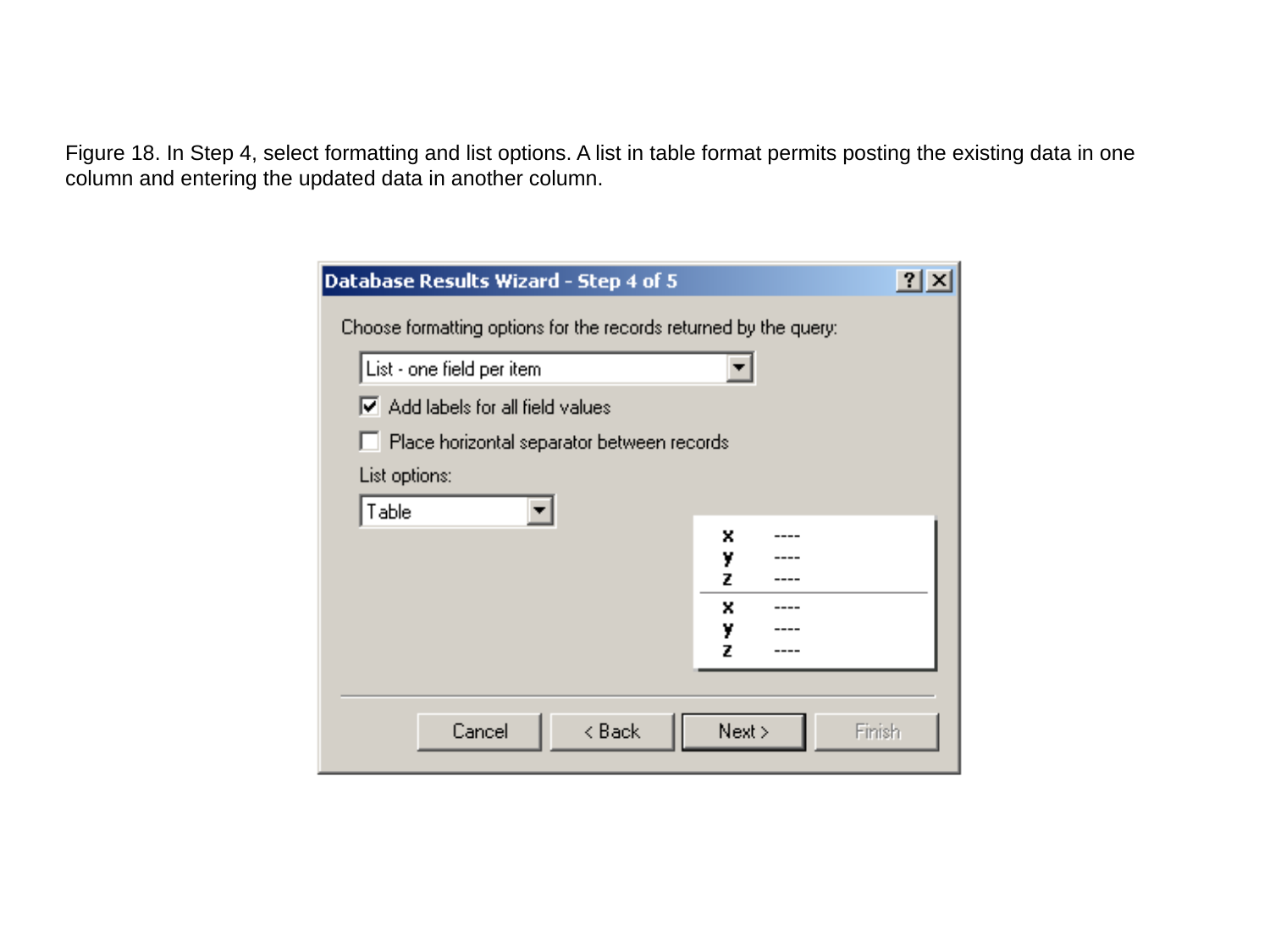

# Figure 18. In Step 4, select formatting and list options. A list in table format permits posting the existing data in one column and entering the updated data in another column.

## Slide 19
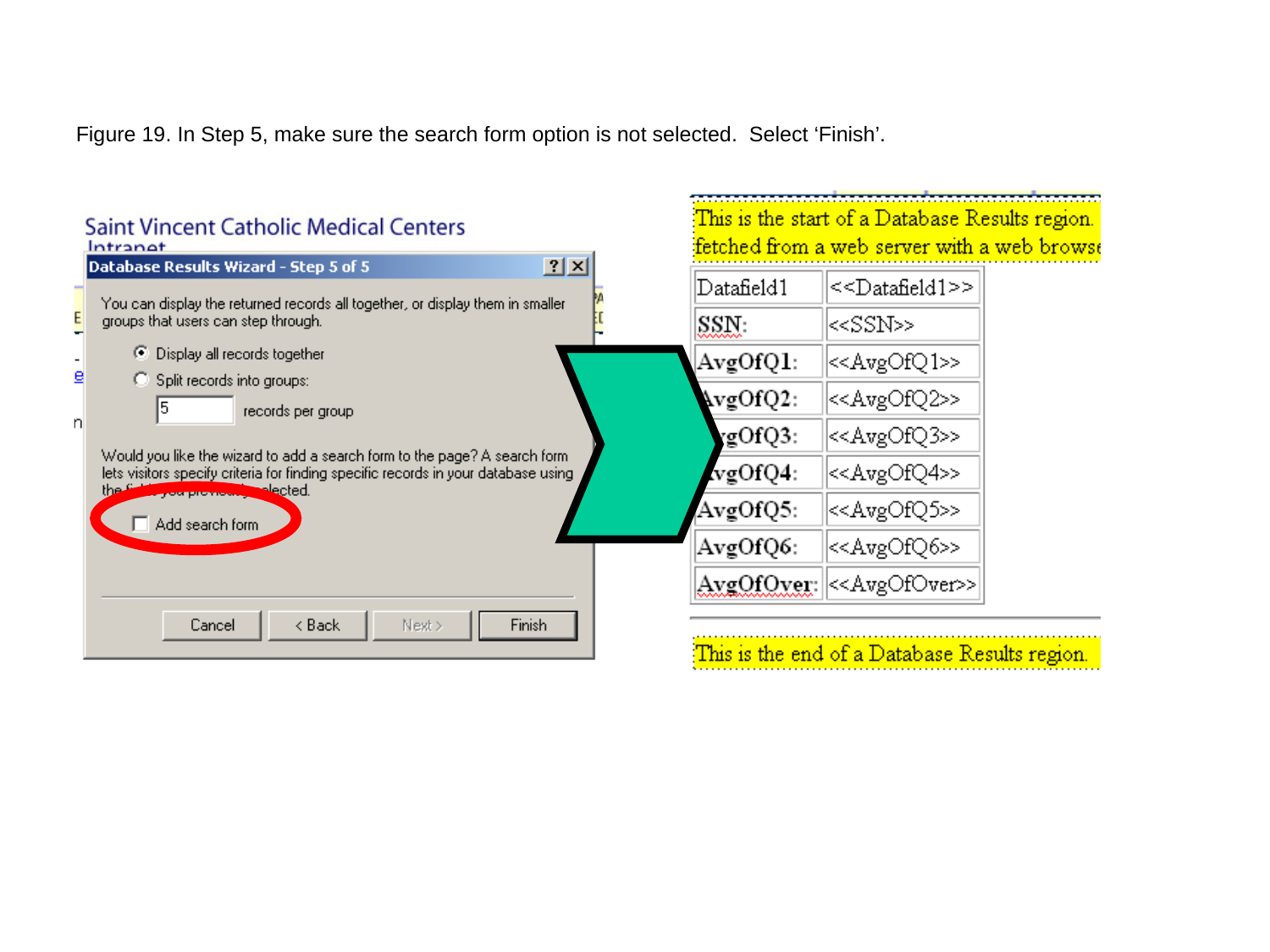

# Figure 19. In Step 5, make sure the search form option is not selected. Select ‘Finish’.

## Slide 20
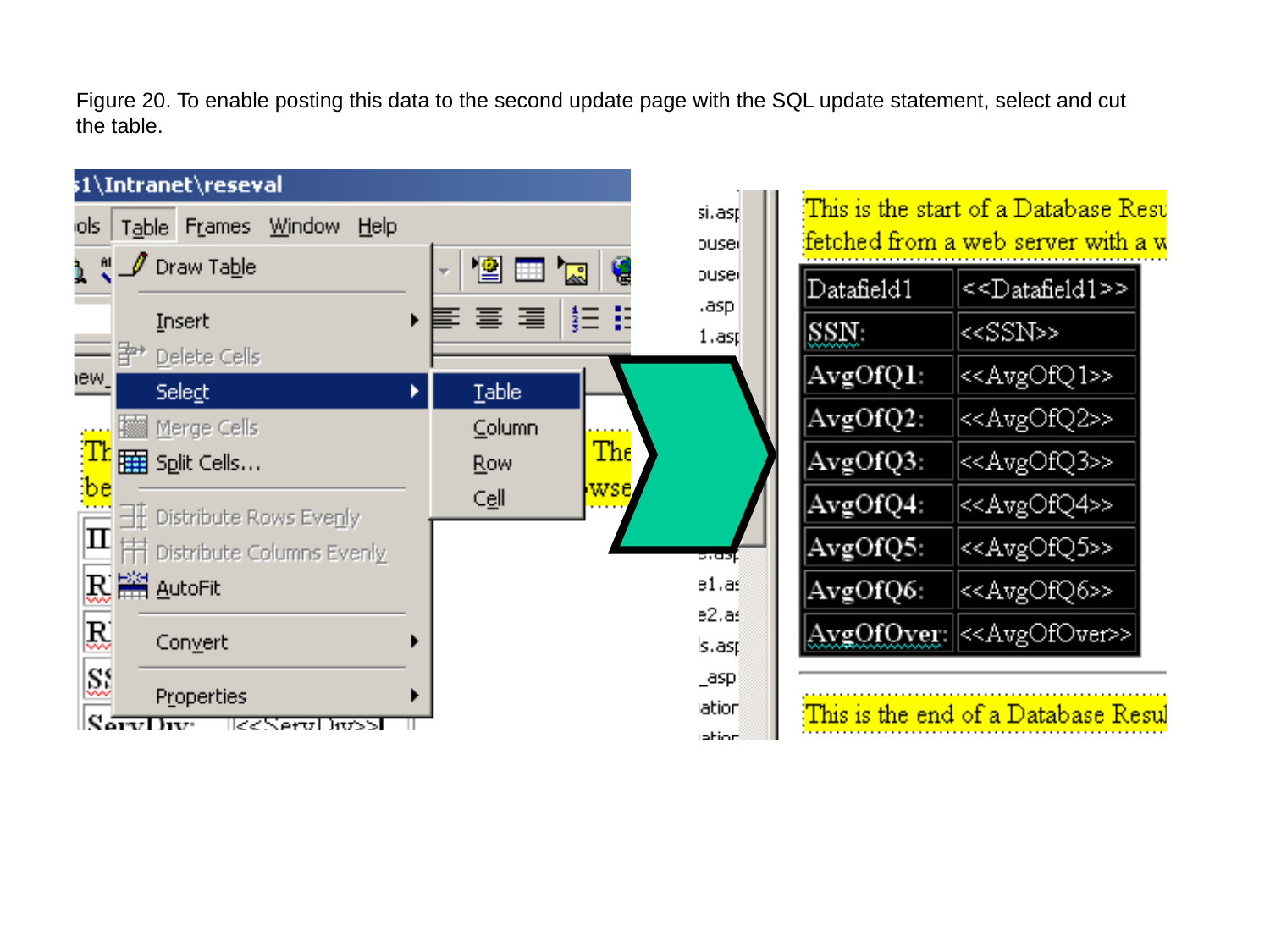

# Figure 20. To enable posting this data to the second update page with the SQL update statement, select and cut the table.

## Slide 21
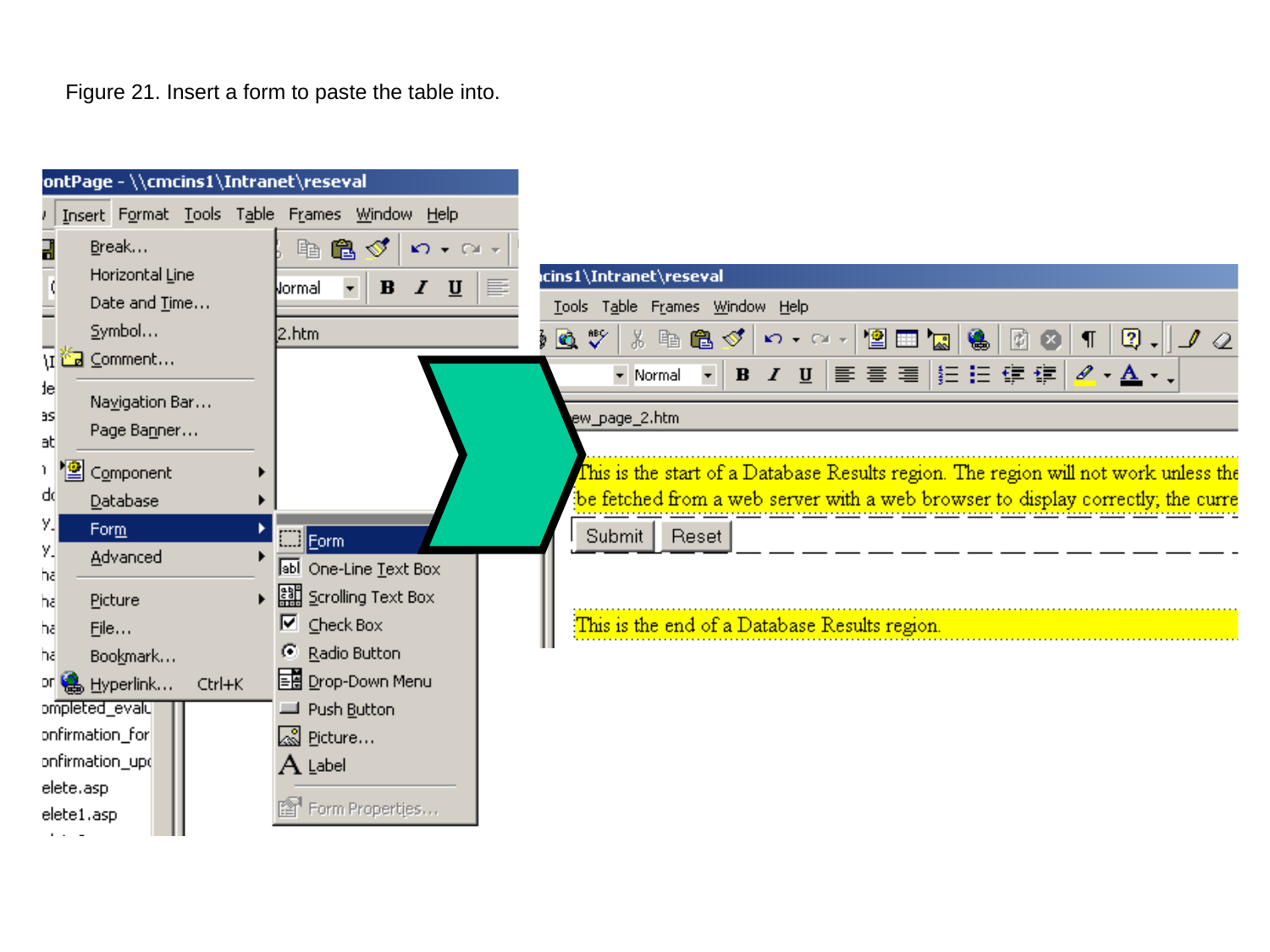

# Figure 21. Insert a form to paste the table into.

## Slide 22
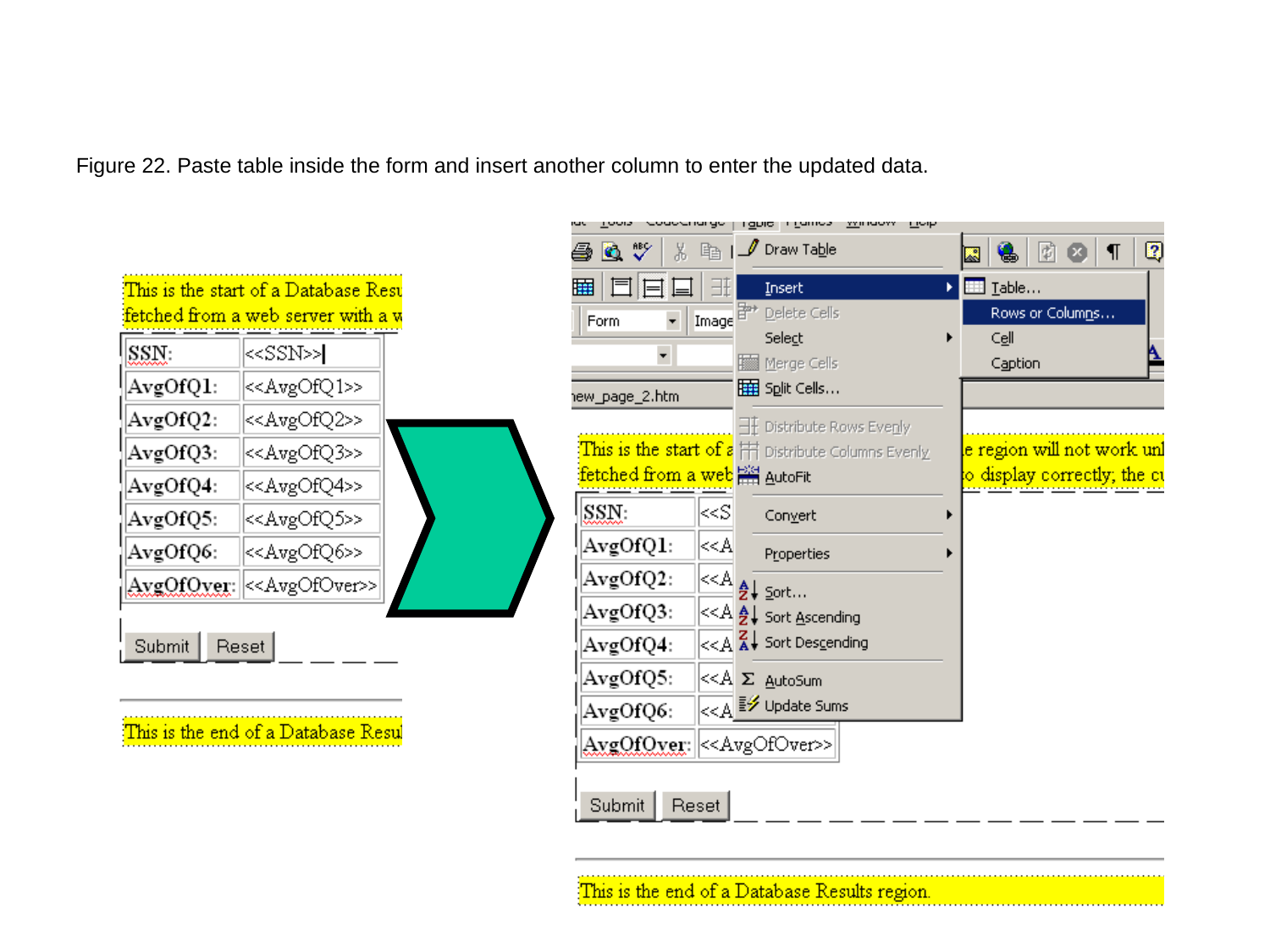

# Figure 22. Paste table inside the form and insert another column to enter the updated data.

## Slide 23
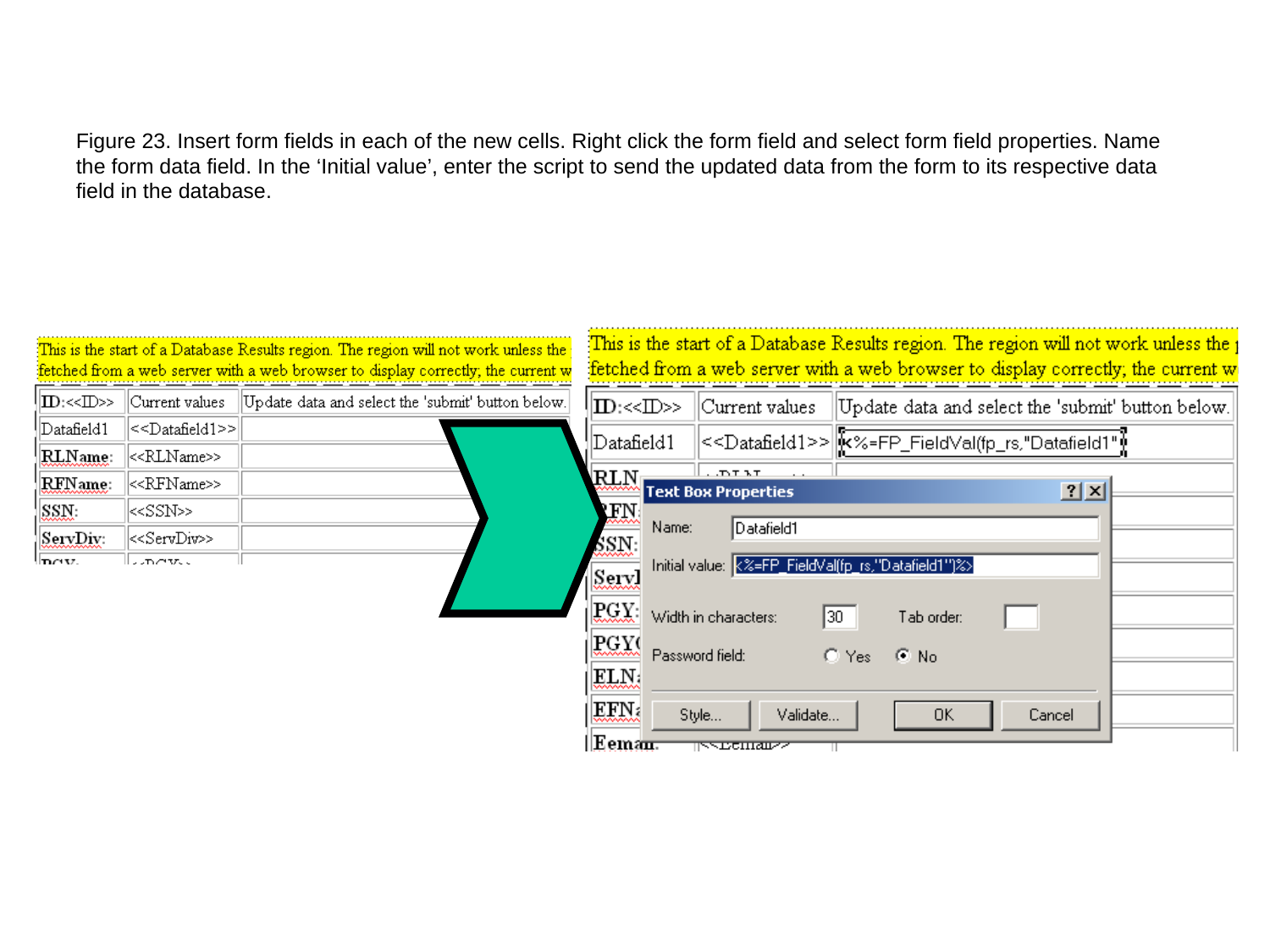

# Figure 23. Insert form fields in each of the new cells. Right click the form field and select form field properties. Name the form data field. In the ‘Initial value’, enter the script to send the updated data from the form to its respective data field in the database.

## Slide 24
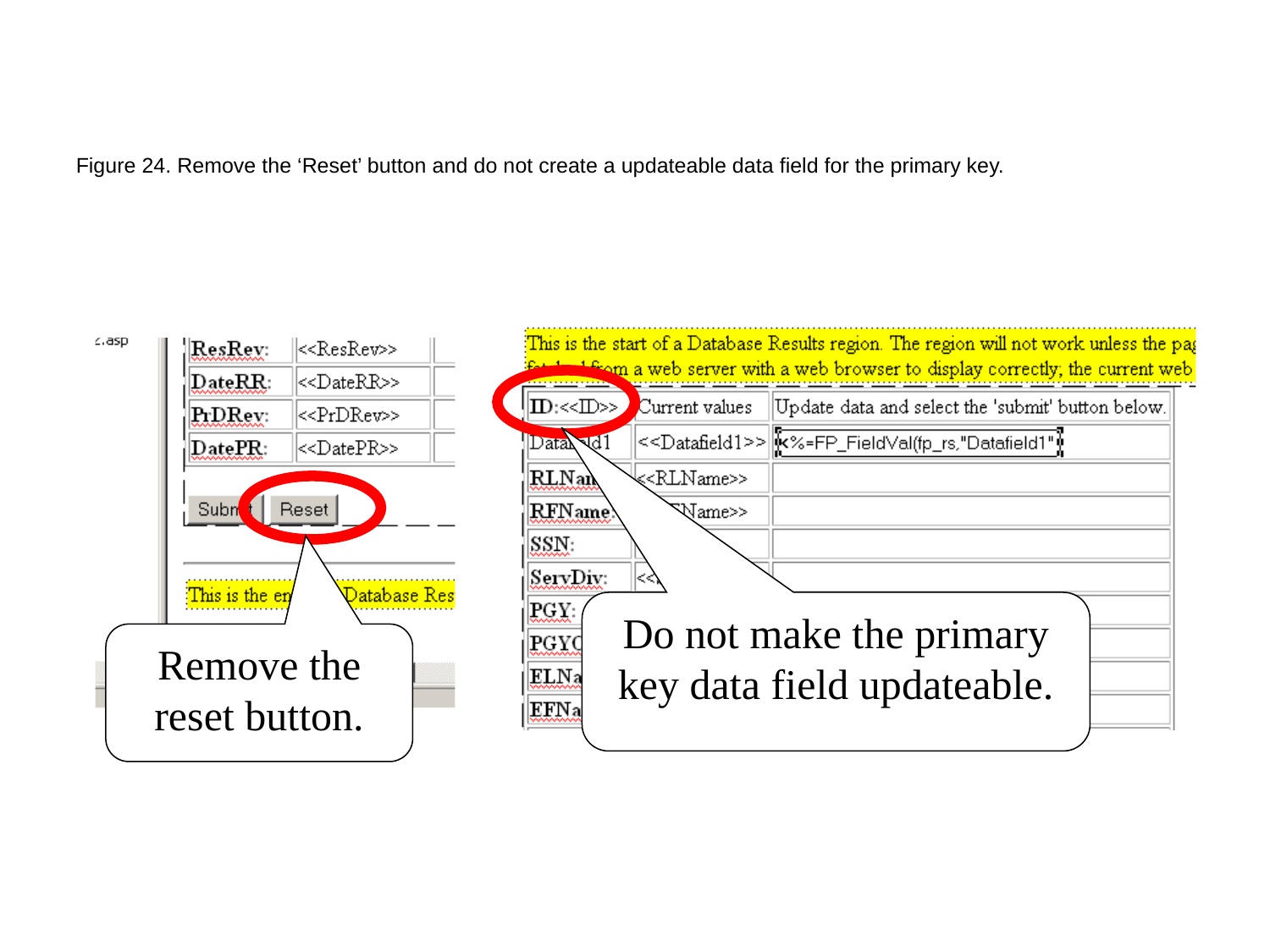

# Figure 24. Remove the ‘Reset’ button and do not create a updateable data field for the primary key.
Do not make the primary key data field updateable.
Remove the reset button.

## Slide 25
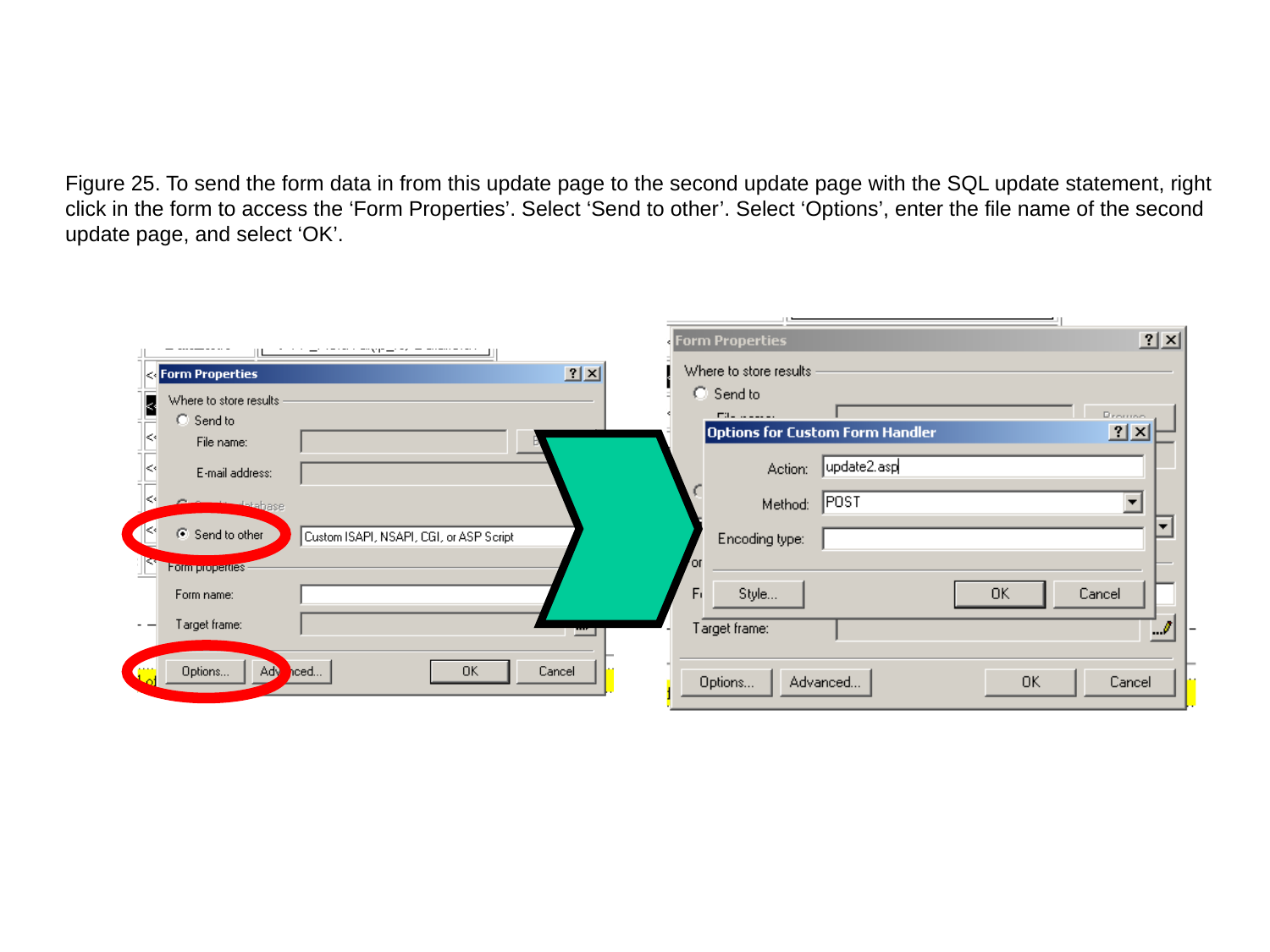

# Figure 25. To send the form data in from this update page to the second update page with the SQL update statement, right click in the form to access the ‘Form Properties’. Select ‘Send to other’. Select ‘Options’, enter the file name of the second update page, and select ‘OK’.

## Slide 26
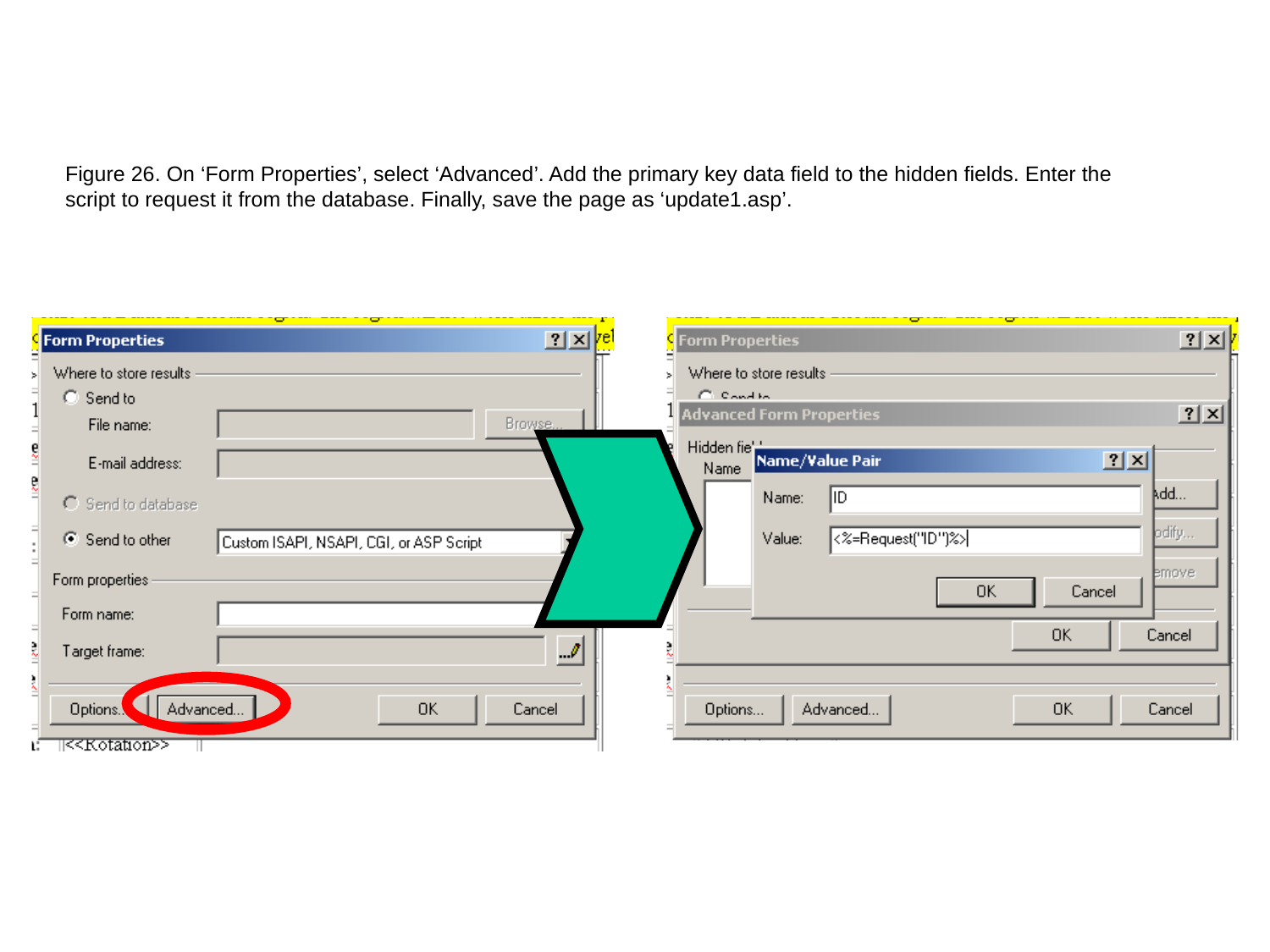

# Figure 26. On ‘Form Properties’, select ‘Advanced’. Add the primary key data field to the hidden fields. Enter the script to request it from the database. Finally, save the page as ‘update1.asp’.

## Slide 27
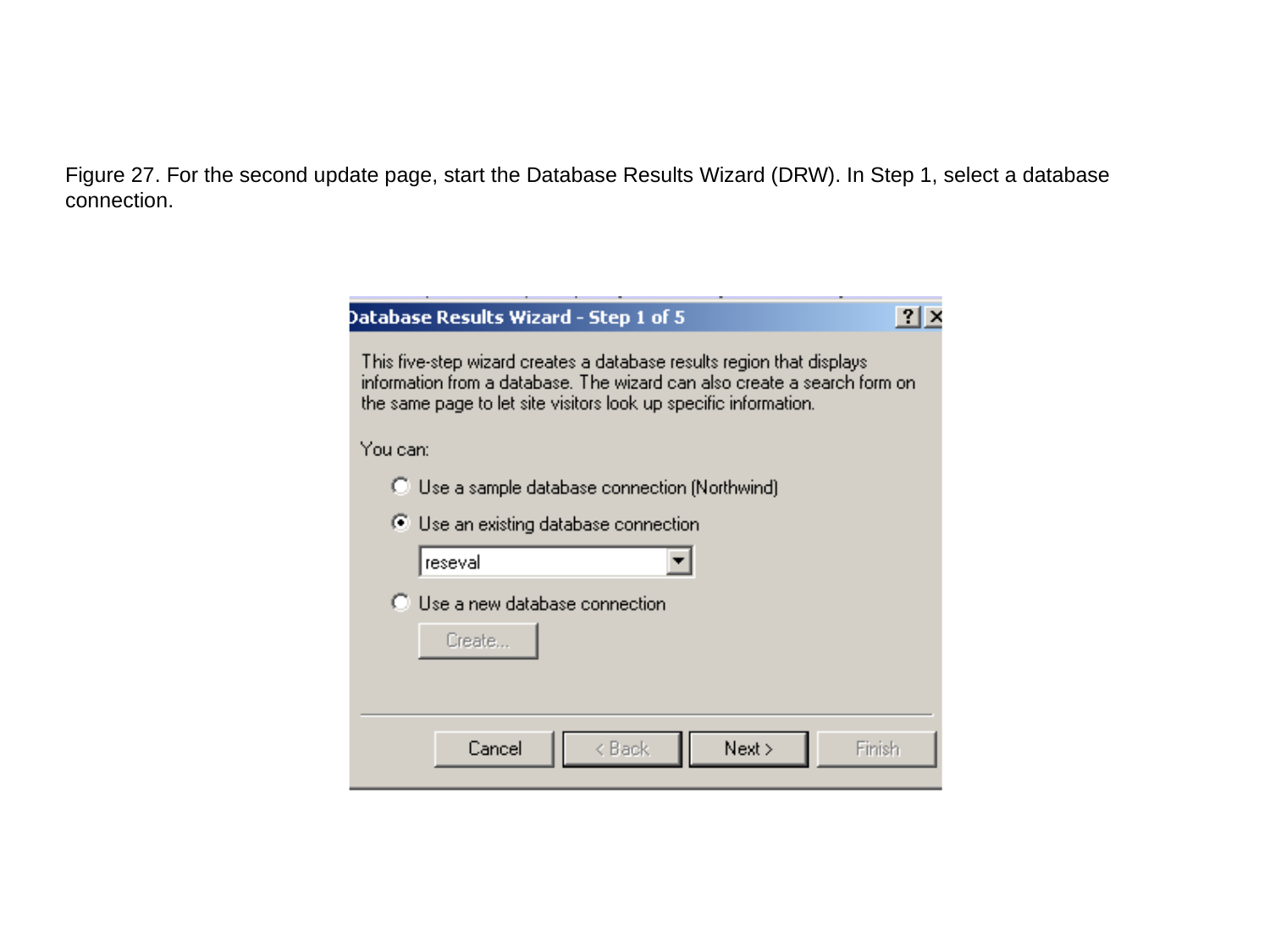

# Figure 27. For the second update page, start the Database Results Wizard (DRW). In Step 1, select a database connection.

## Slide 28
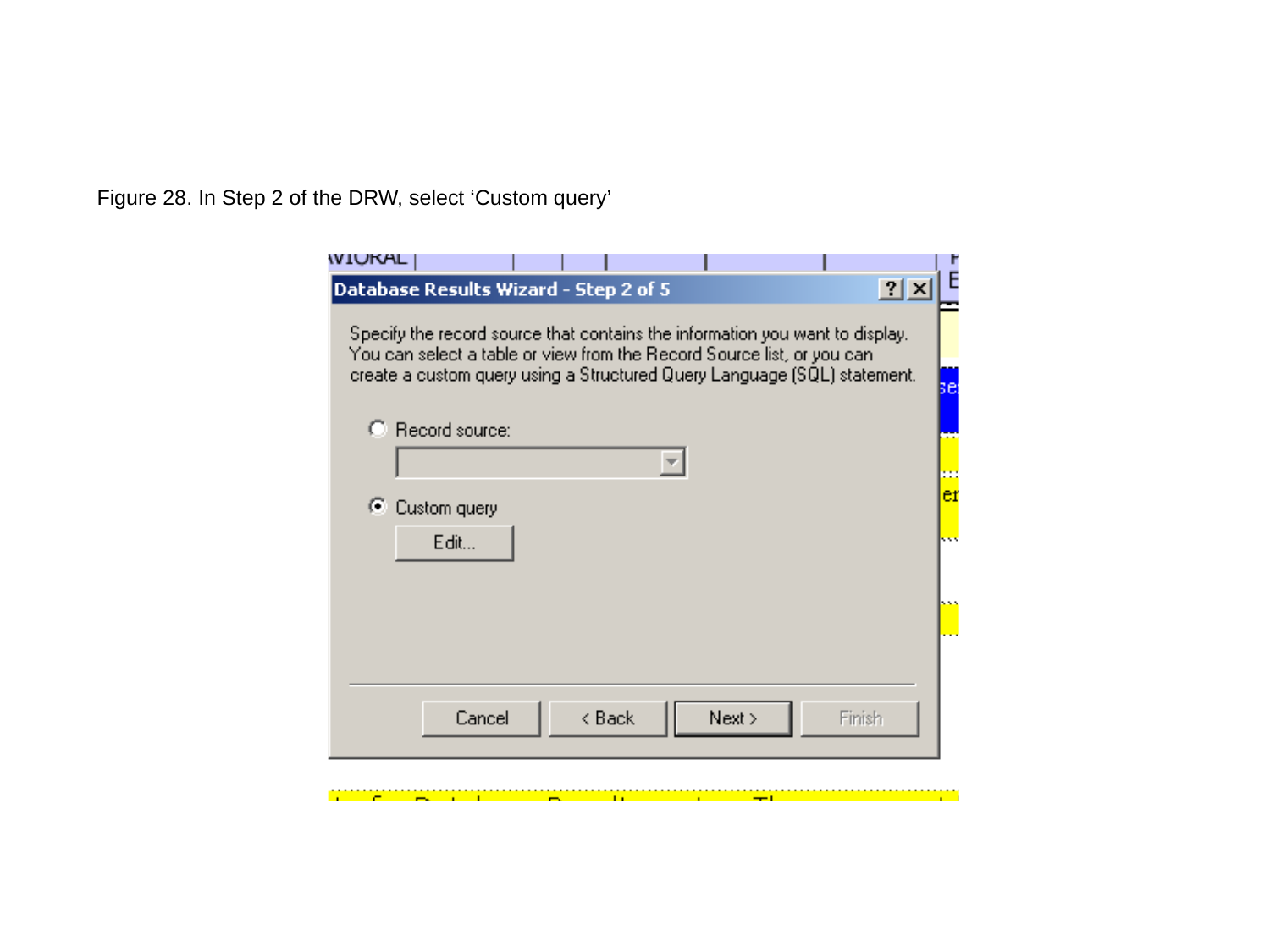

# Figure 28. In Step 2 of the DRW, select ‘Custom query’

## Slide 29
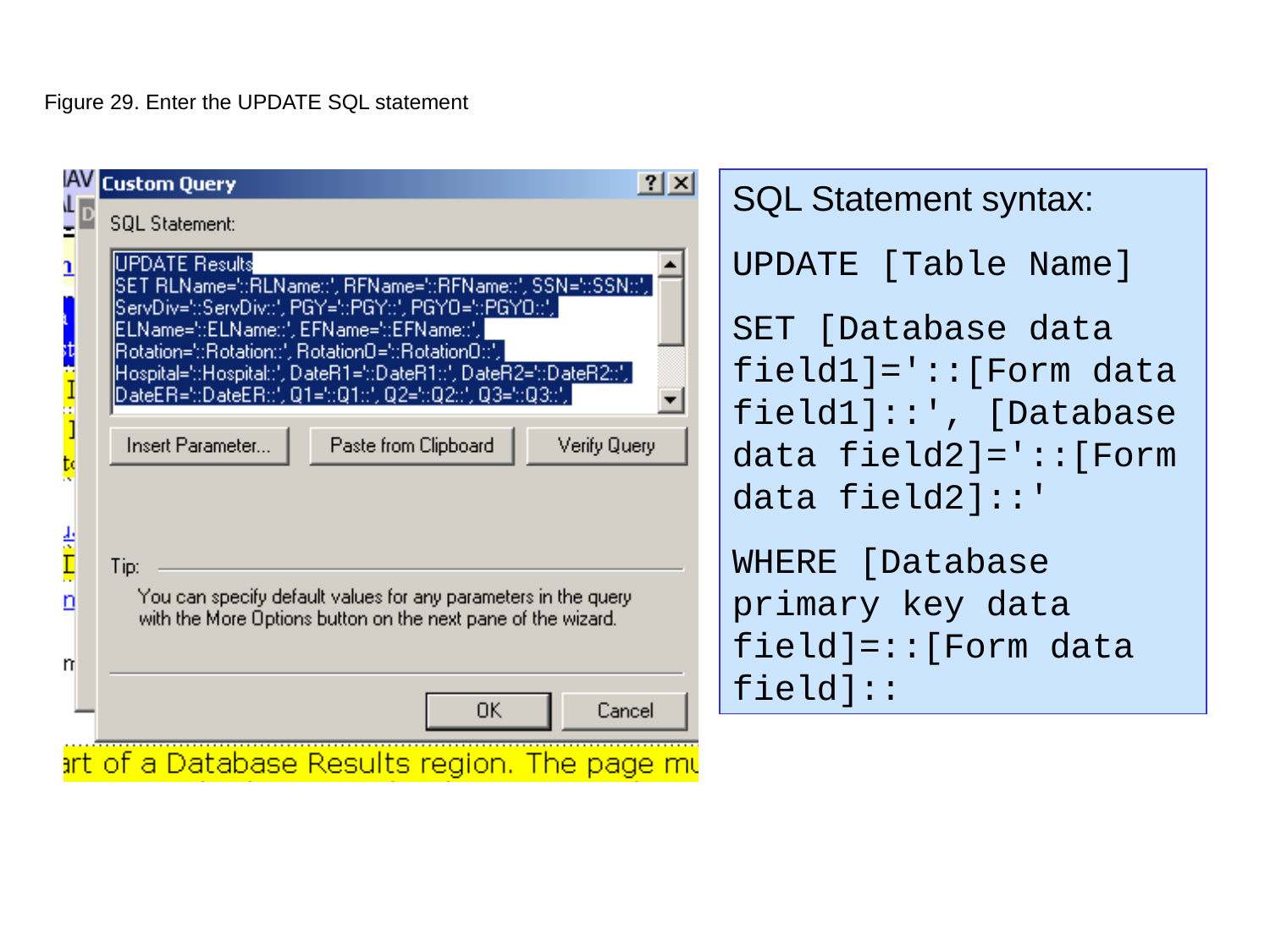

# Figure 29. Enter the UPDATE SQL statement
SQL Statement syntax:
UPDATE [Table Name]
SET [Database data field1]='::[Form data field1]::', [Database data field2]='::[Form data field2]::'
WHERE [Database primary key data field]=::[Form data field]::

## Slide 30
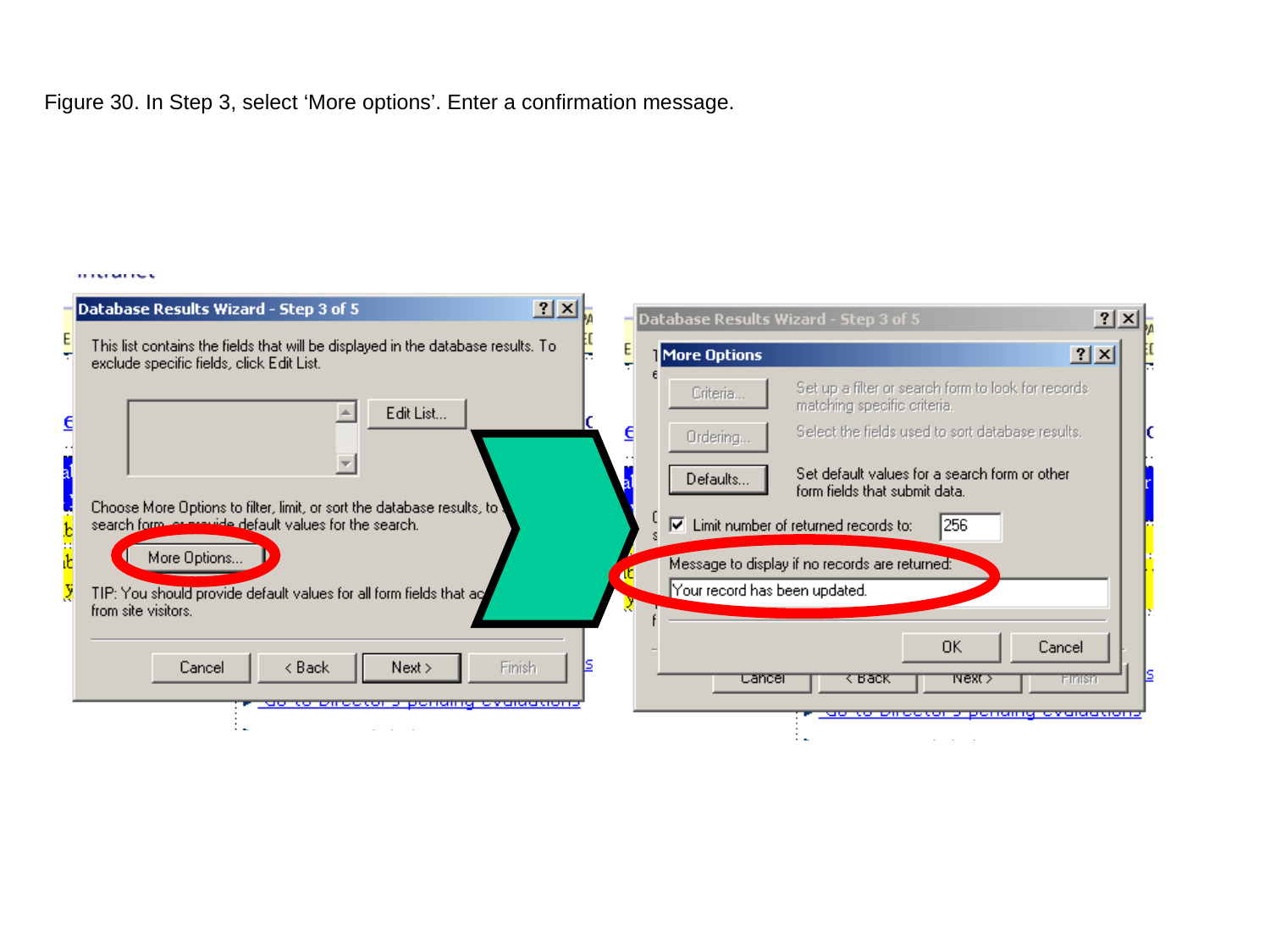

# Figure 30. In Step 3, select ‘More options’. Enter a confirmation message.

## Slide 31
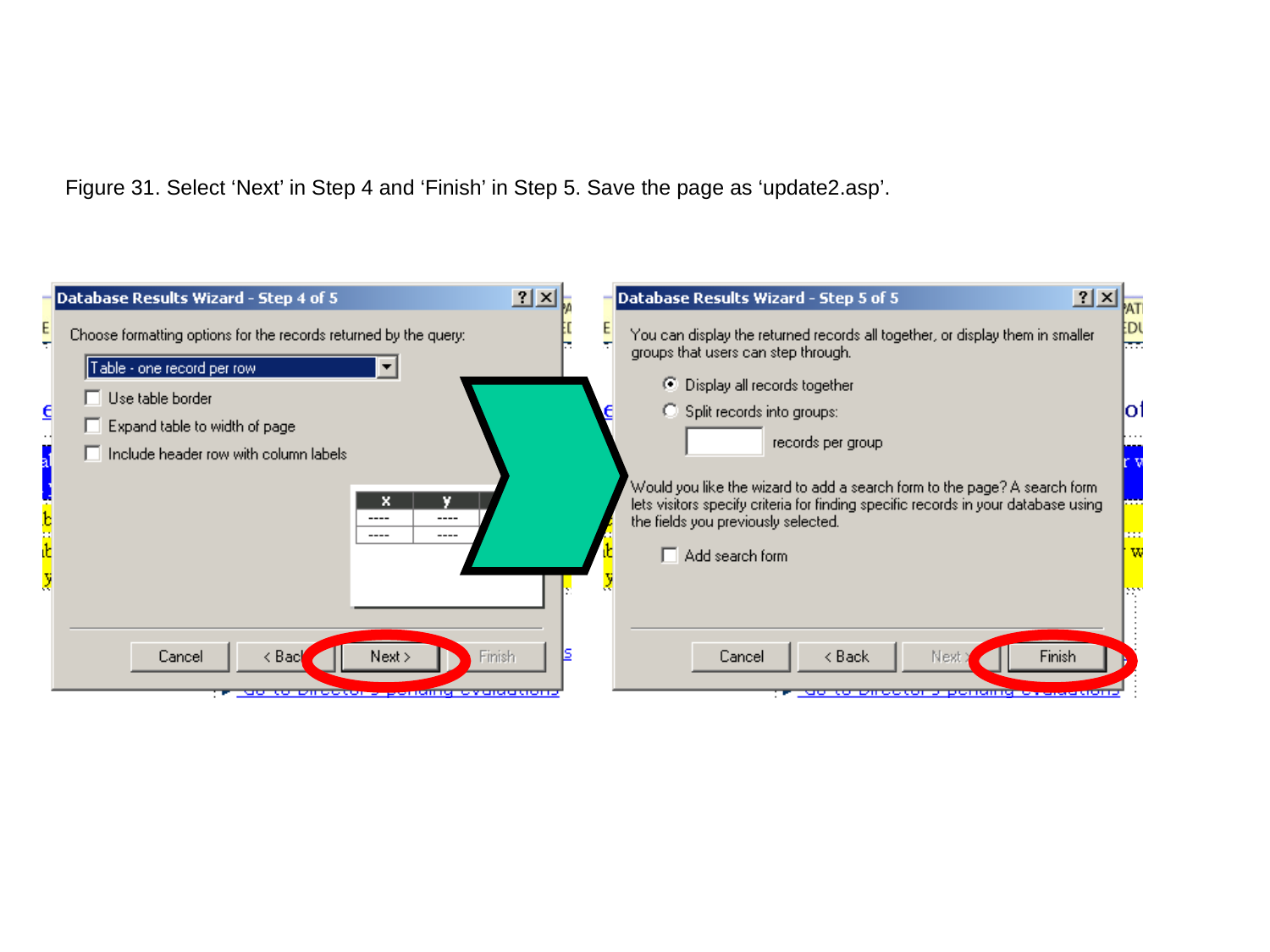

# Figure 31. Select ‘Next’ in Step 4 and ‘Finish’ in Step 5. Save the page as ‘update2.asp’.

## Slide 32
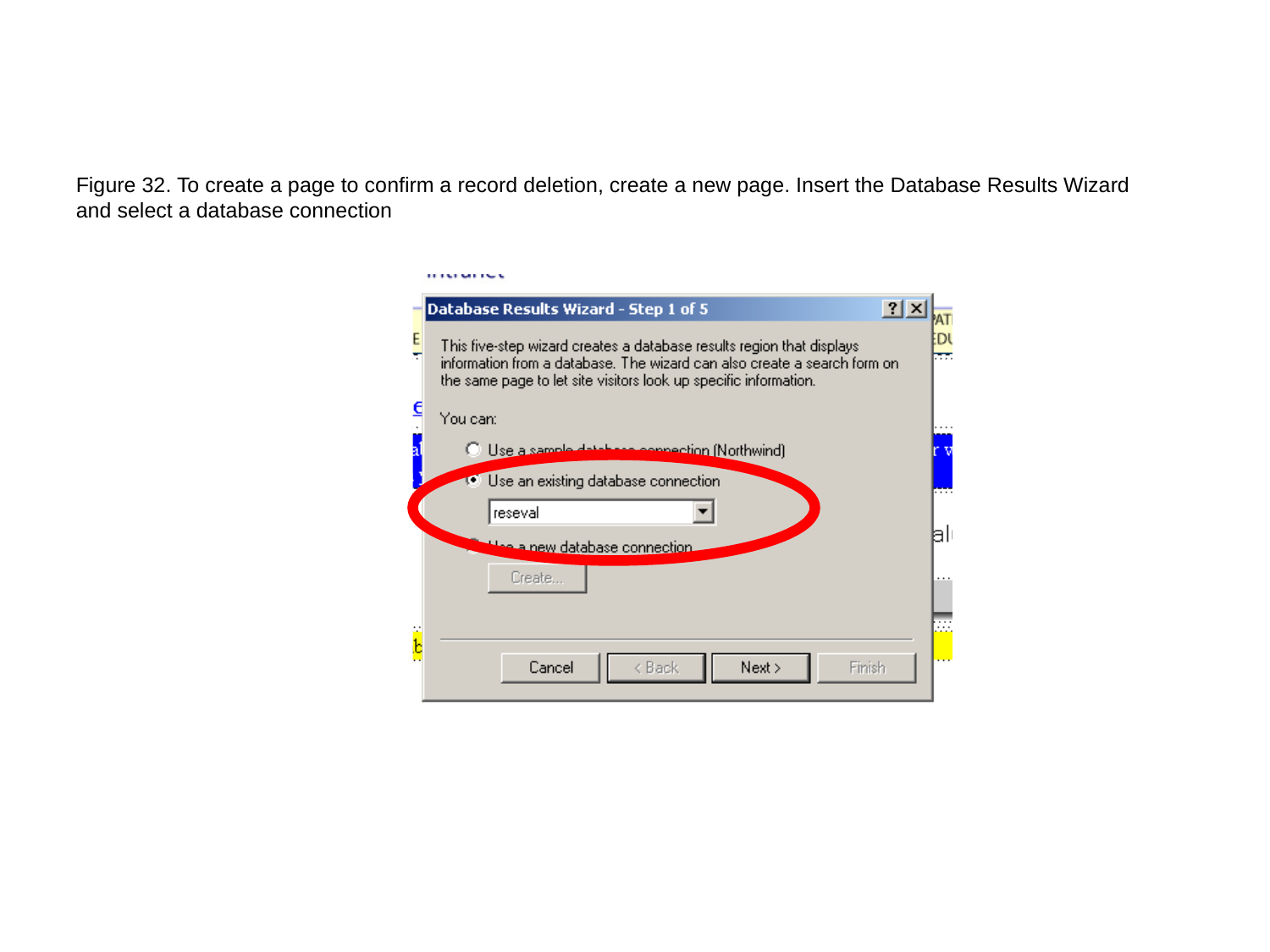

# Figure 32. To create a page to confirm a record deletion, create a new page. Insert the Database Results Wizard and select a database connection

## Slide 33
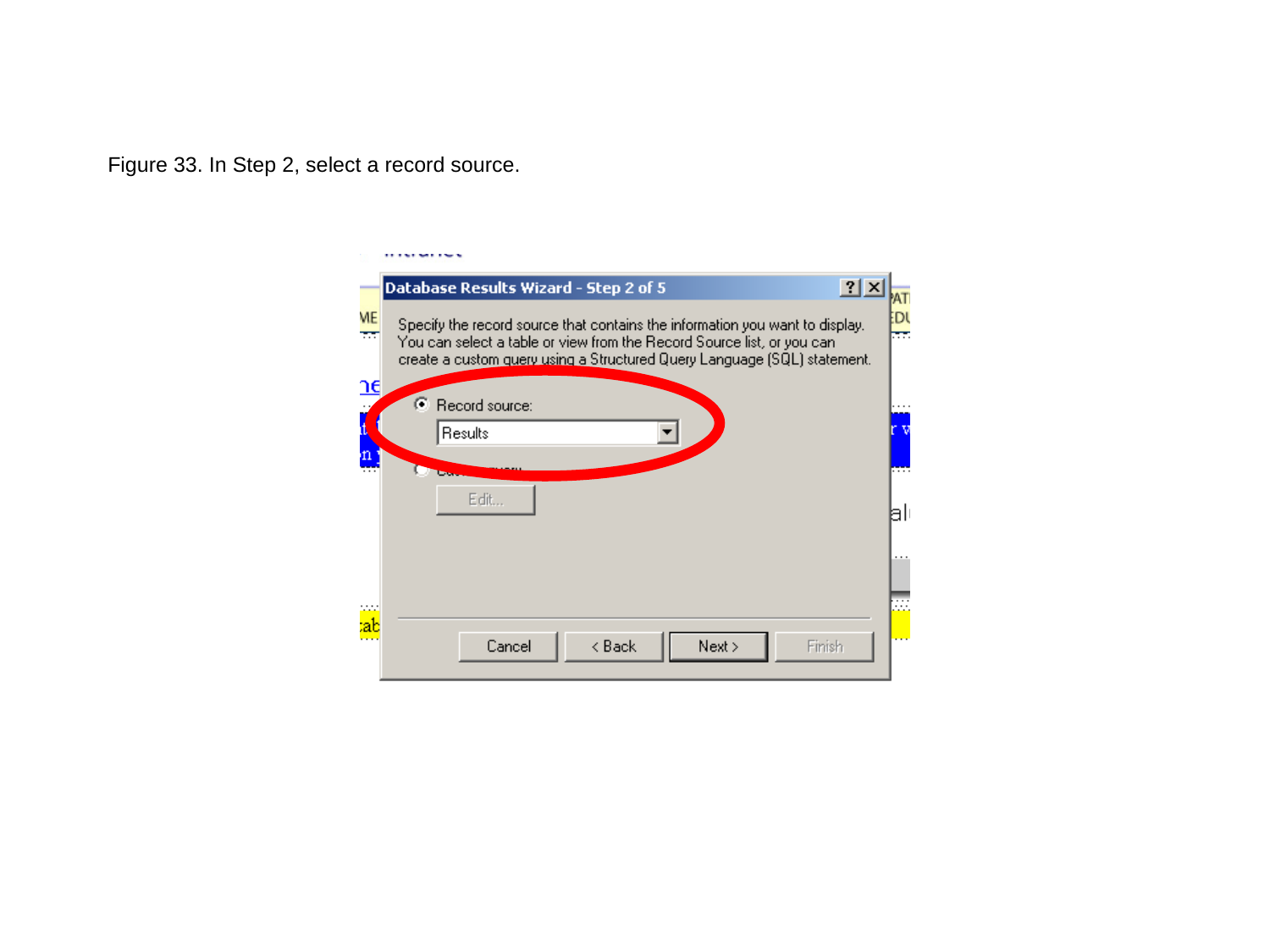

# Figure 33. In Step 2, select a record source.

## Slide 34
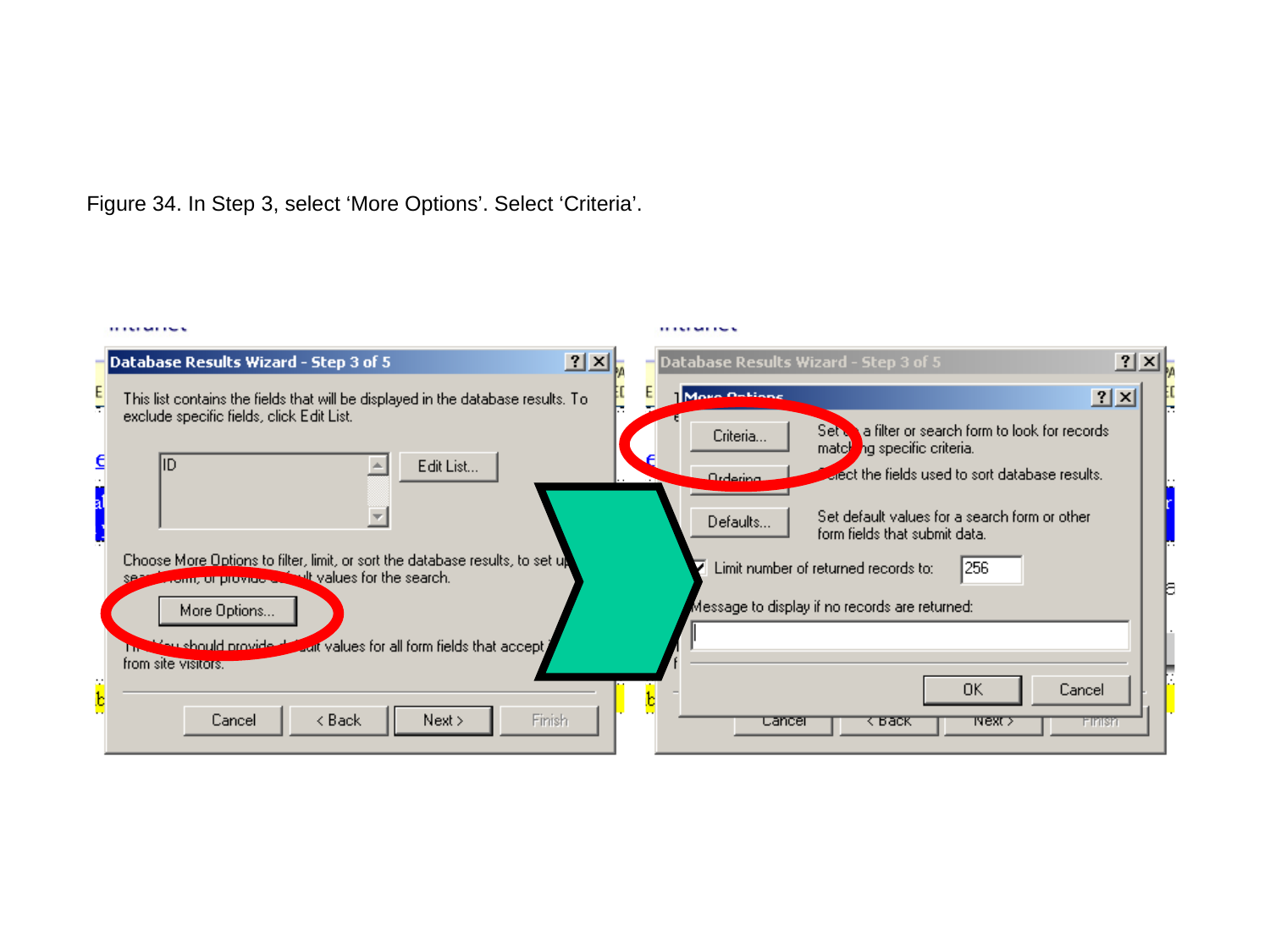

Figure 34. In Step 3, select ‘More Options’. Select ‘Criteria’.

## Slide 35
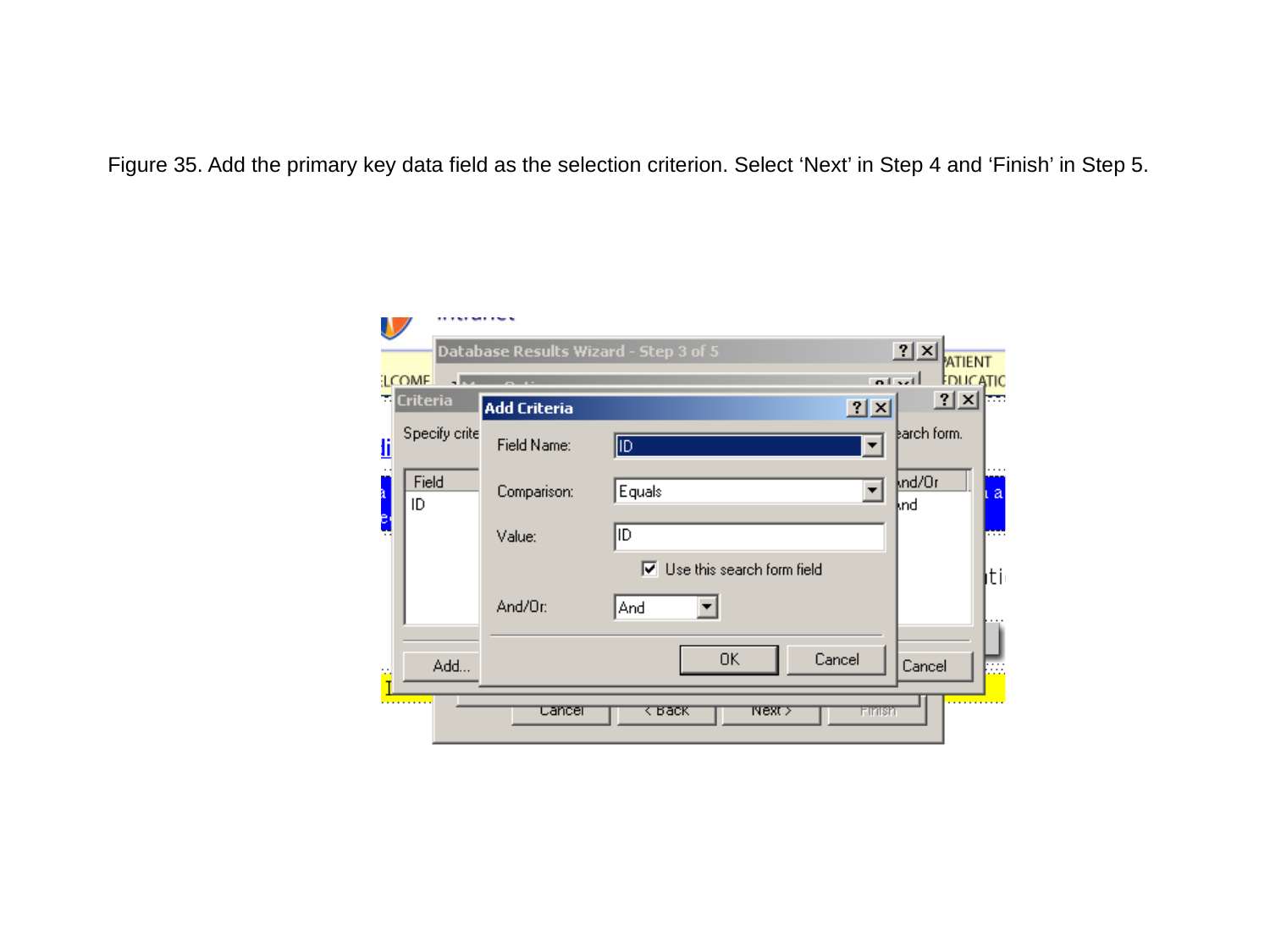

# Figure 35. Add the primary key data field as the selection criterion. Select ‘Next’ in Step 4 and ‘Finish’ in Step 5.

## Slide 36
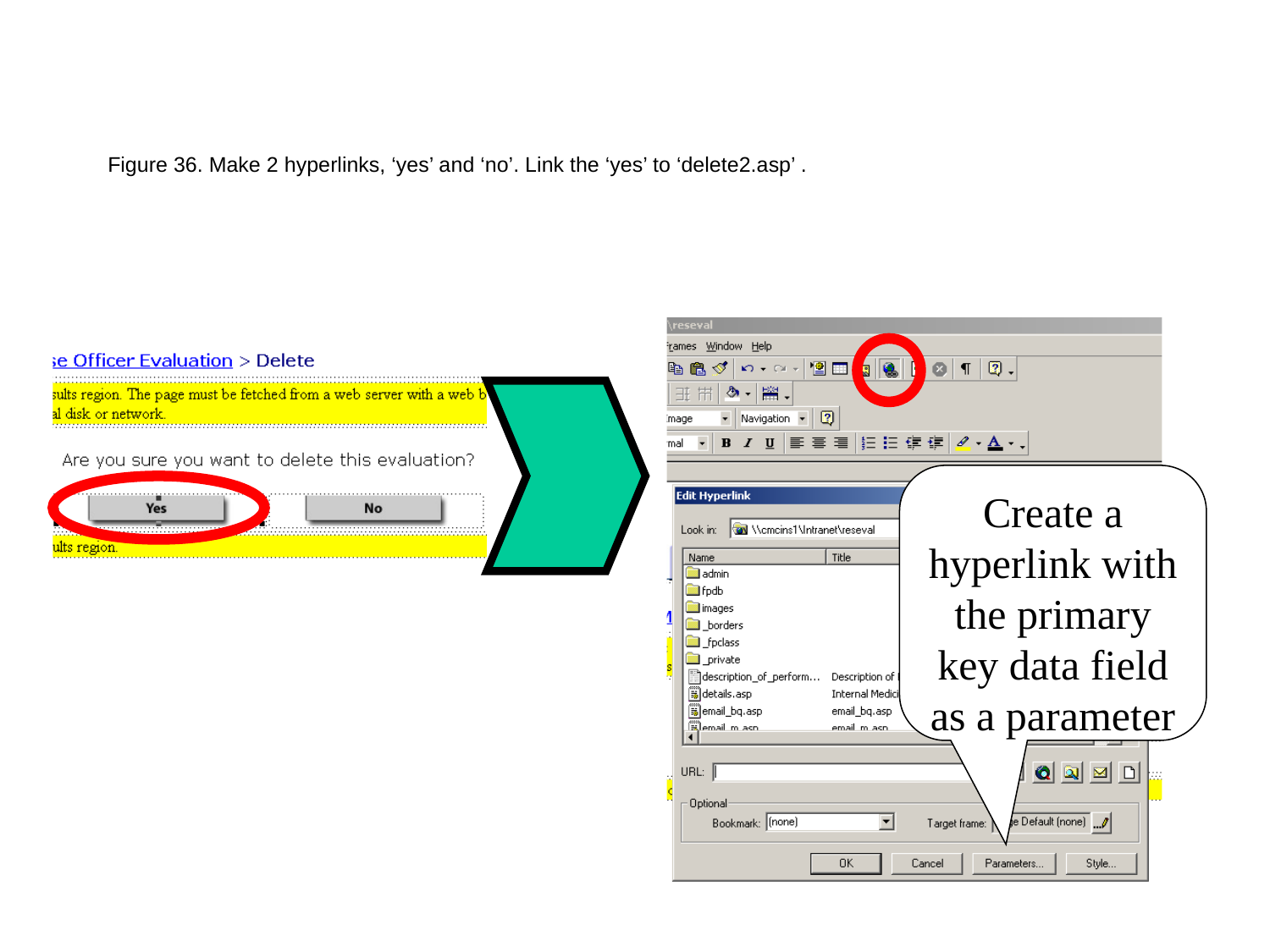

# Figure 36. Make 2 hyperlinks, ‘yes’ and ‘no’. Link the ‘yes’ to ‘delete2.asp’ .
Create a hyperlink with the primary key data field as a parameter

## Slide 37
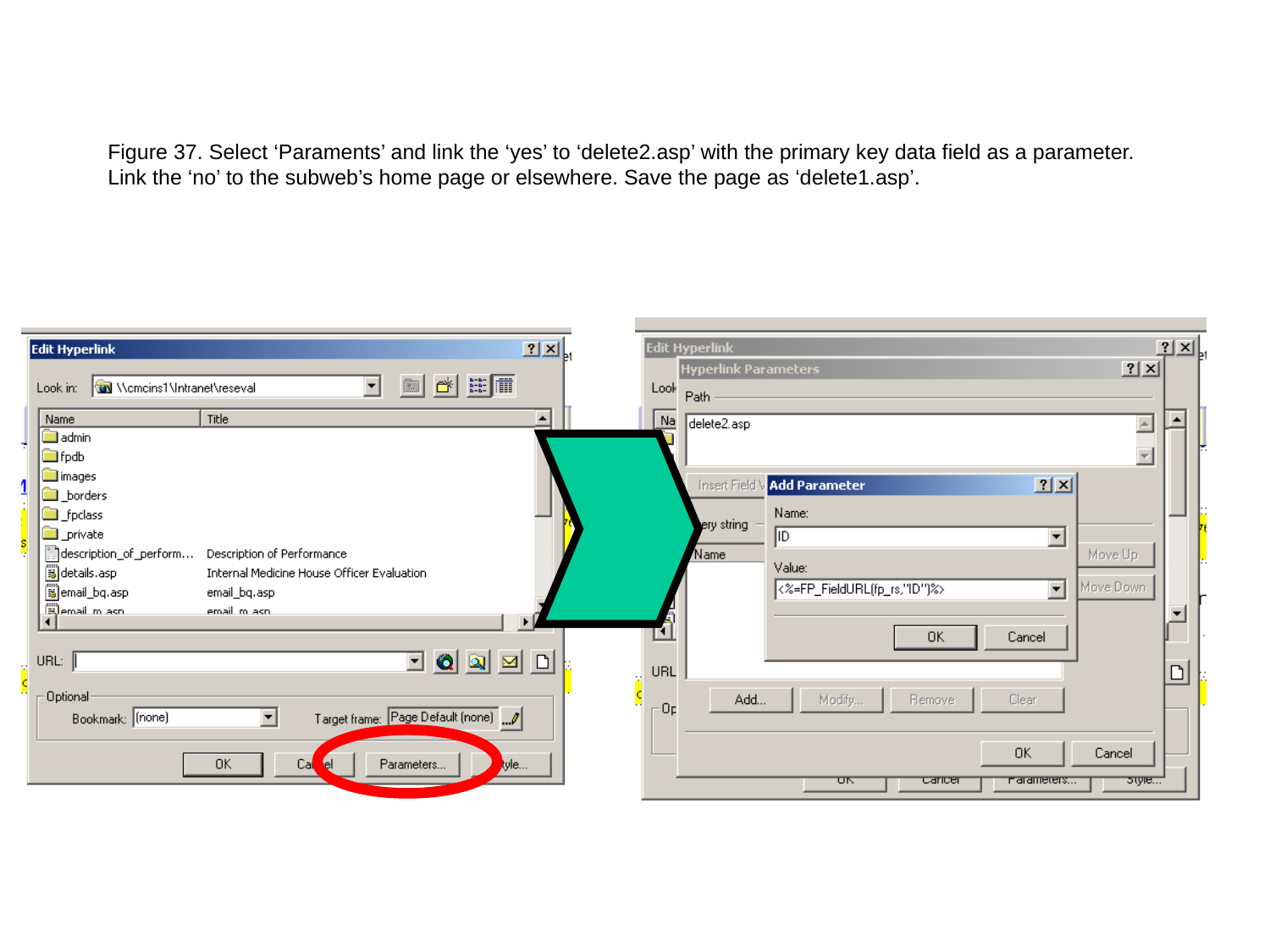

# Figure 37. Select ‘Paraments’ and link the ‘yes’ to ‘delete2.asp’ with the primary key data field as a parameter. Link the ‘no’ to the subweb’s home page or elsewhere. Save the page as ‘delete1.asp’.

## Slide 38
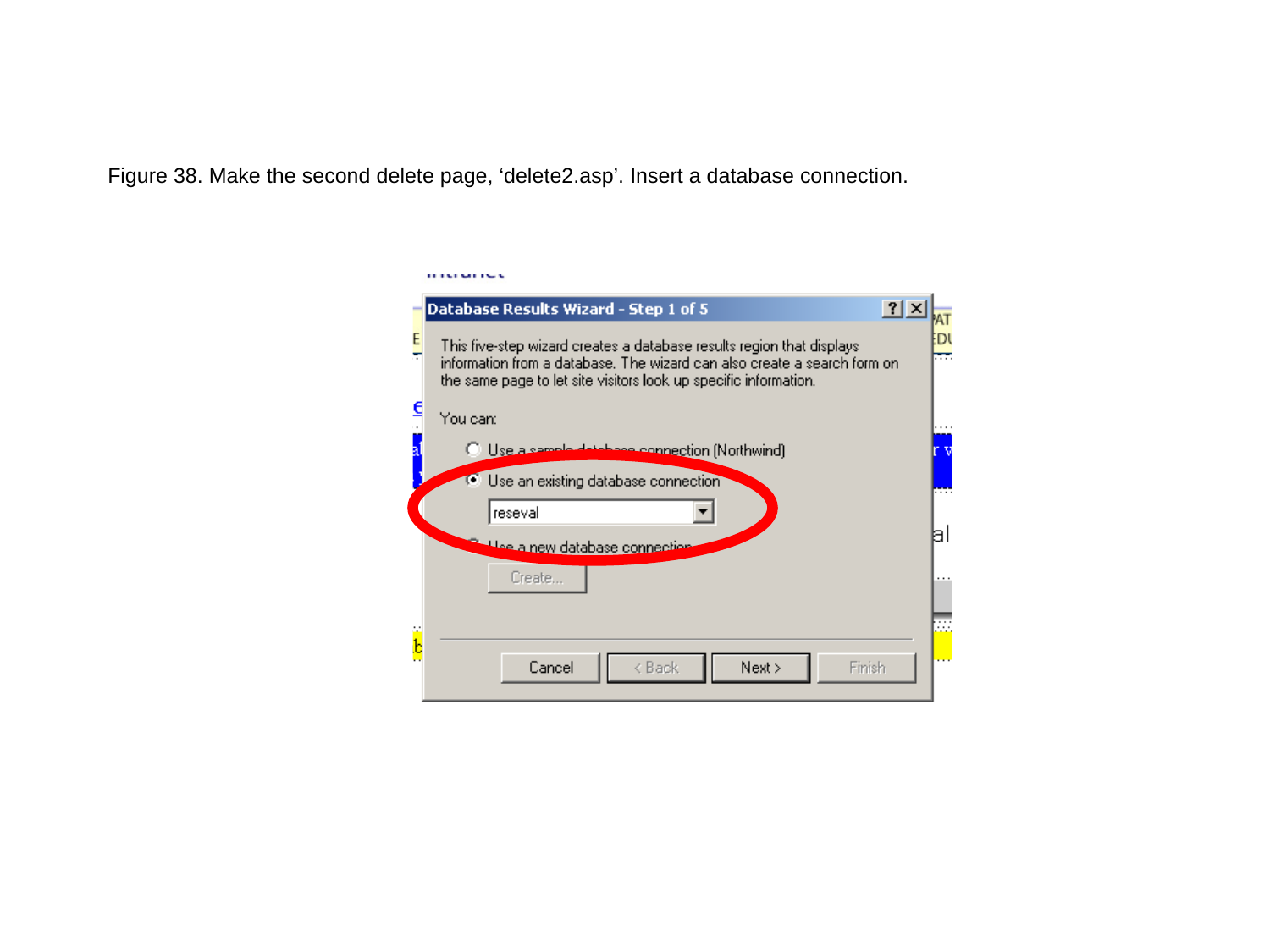

# Figure 38. Make the second delete page, ‘delete2.asp’. Insert a database connection.

## Slide 39
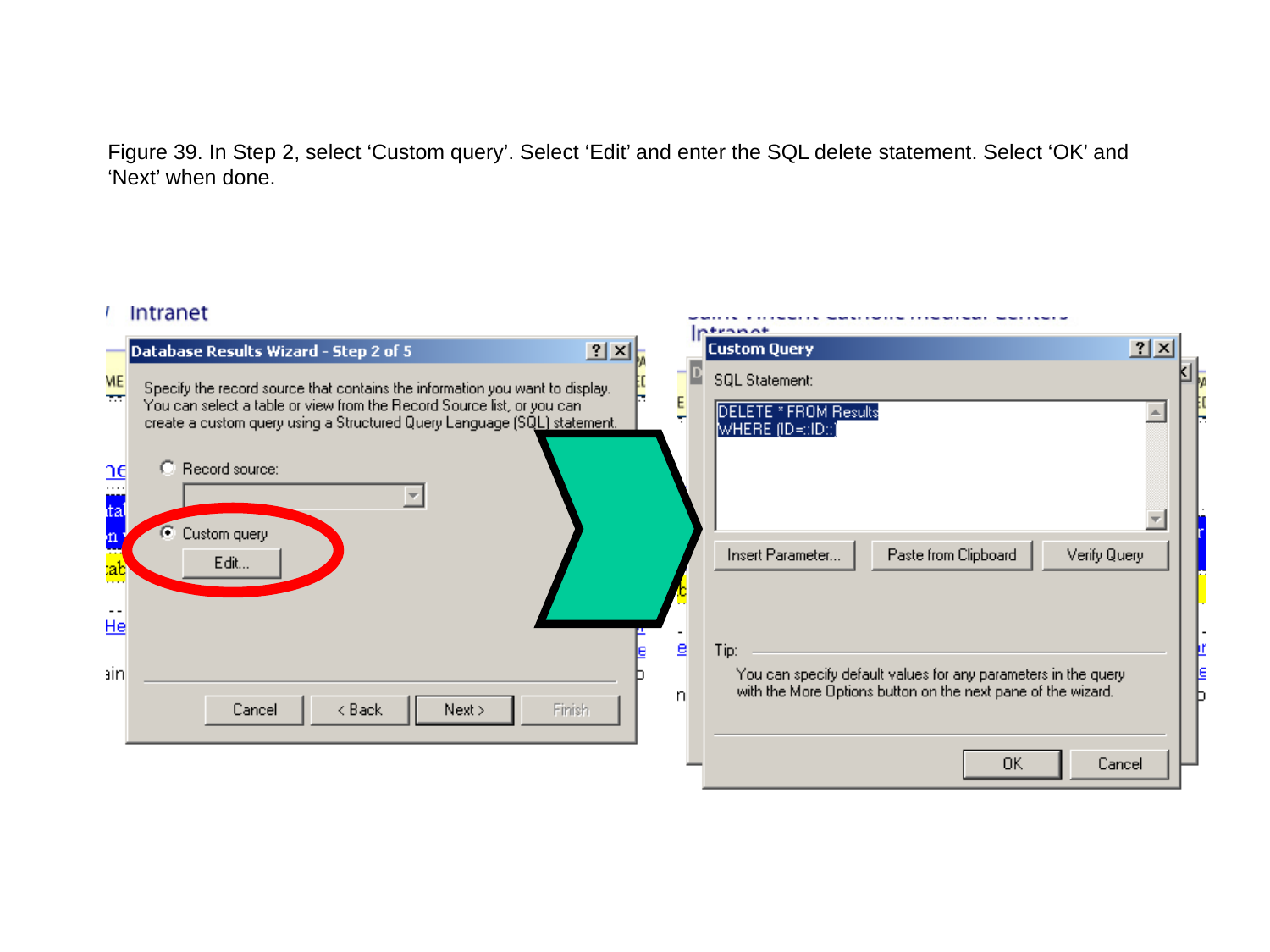

# Figure 39. In Step 2, select ‘Custom query’. Select ‘Edit’ and enter the SQL delete statement. Select ‘OK’ and ‘Next’ when done.

## Slide 40
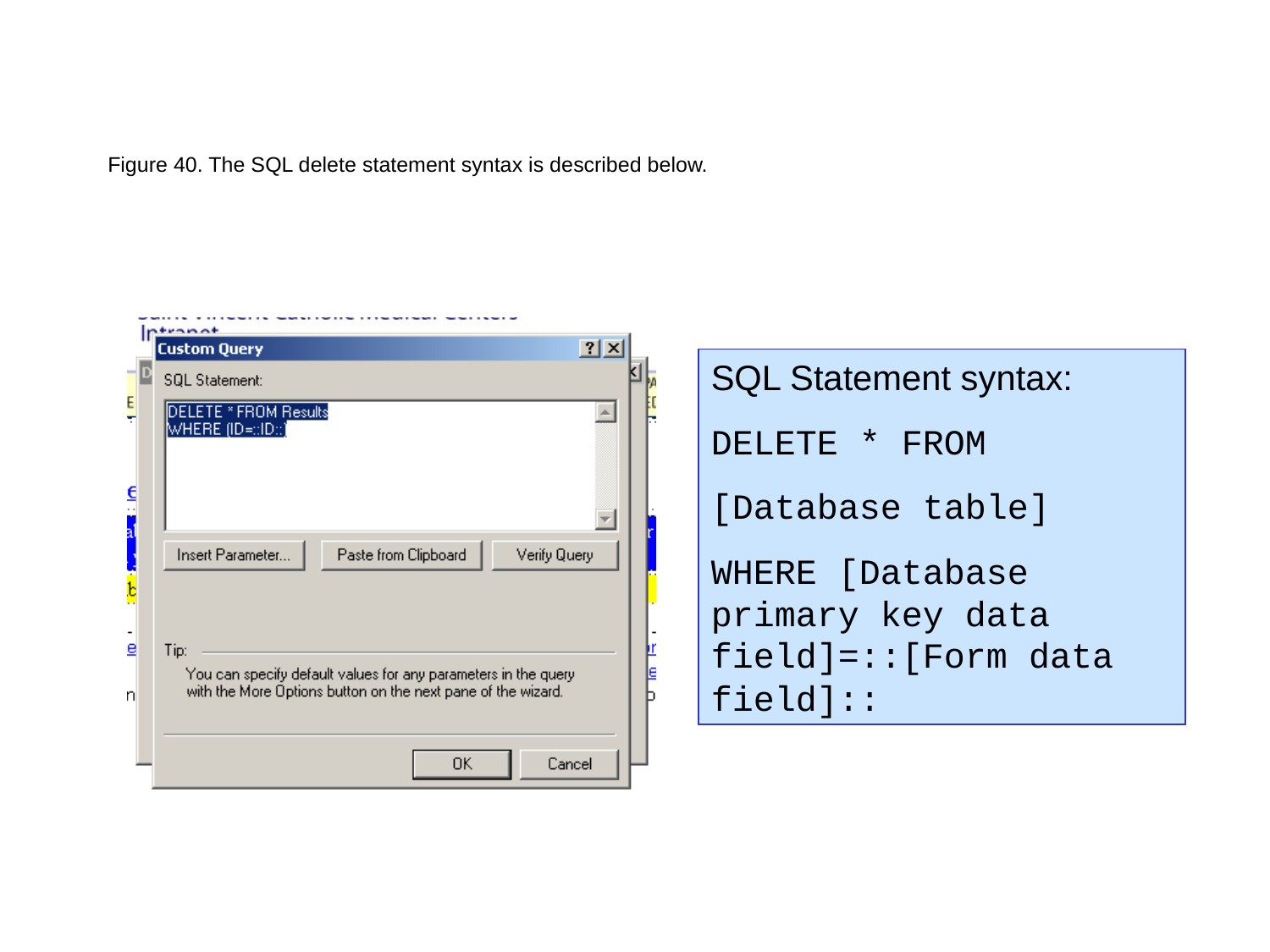

# Figure 40. The SQL delete statement syntax is described below.
SQL Statement syntax:
DELETE * FROM
[Database table]
WHERE [Database primary key data field]=::[Form data field]::

## Slide 41
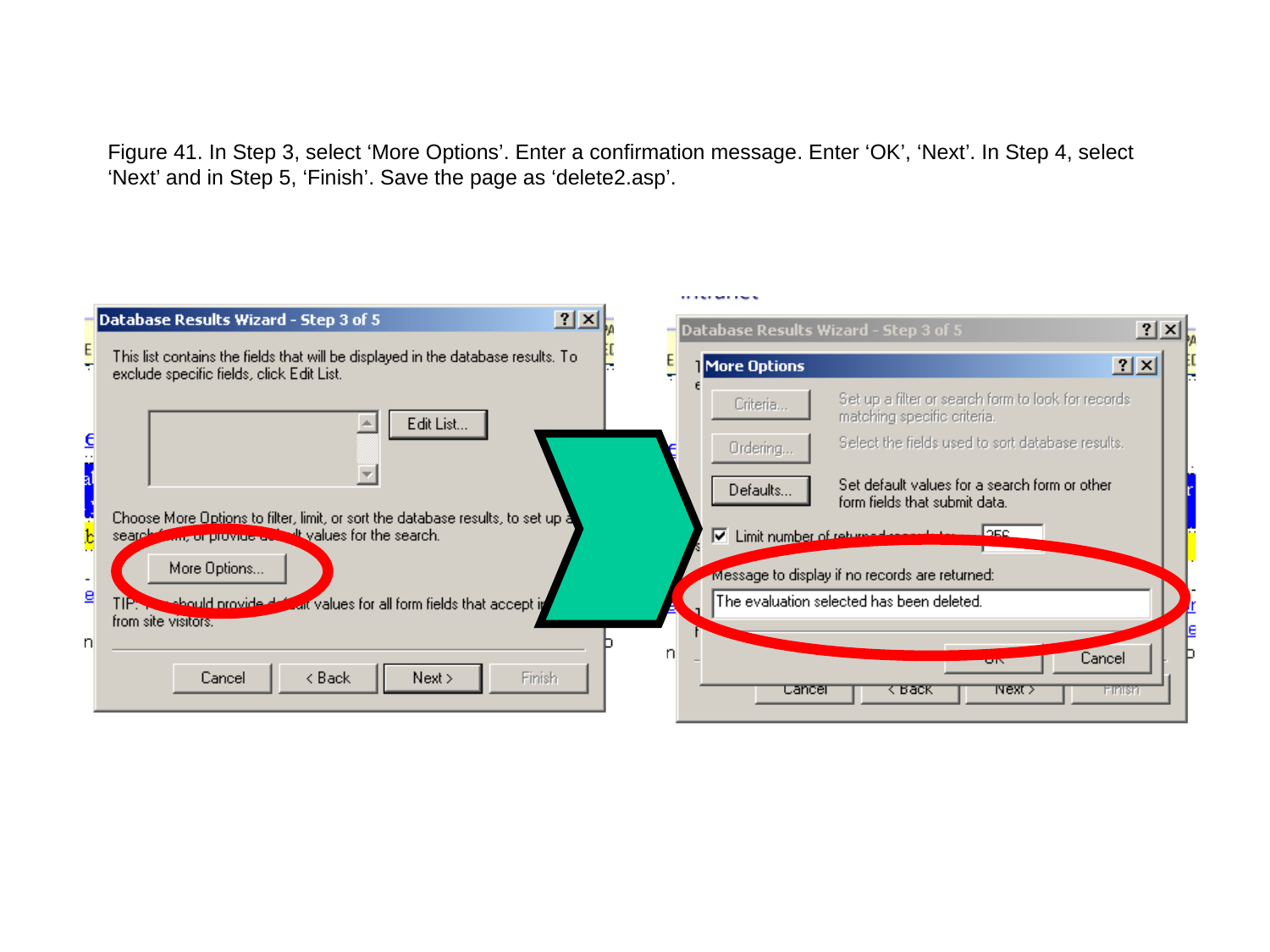

# Figure 41. In Step 3, select ‘More Options’. Enter a confirmation message. Enter ‘OK’, ‘Next’. In Step 4, select ‘Next’ and in Step 5, ‘Finish’. Save the page as ‘delete2.asp’.
