# Supplementary material for: Just-in-time Database-Driven Web Applications
Source: J Med Internet Res. 2003 Aug 29;5(3):e18. doi: 10.2196/jmir.5.3.e18 (PMC1550565; doi:10.2196/jmir.5.3.e18)
Supplement: Supplementary file 2 [file jmir_v5i3e18_app2.ppt]

## Slide 1
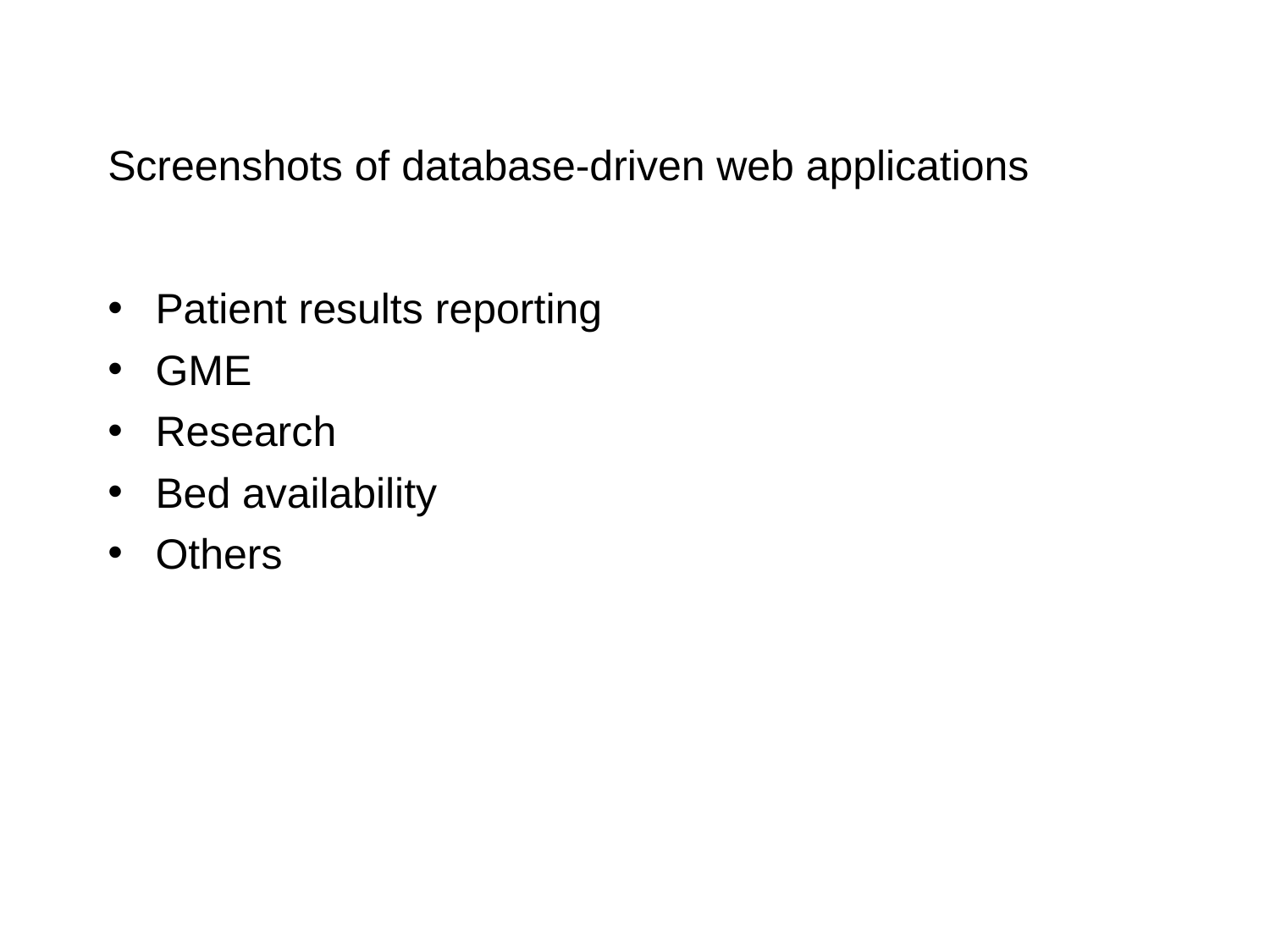

# Screenshots of database-driven web applications
Patient results reporting
GME
Research
Bed availability
Others

## Slide 2
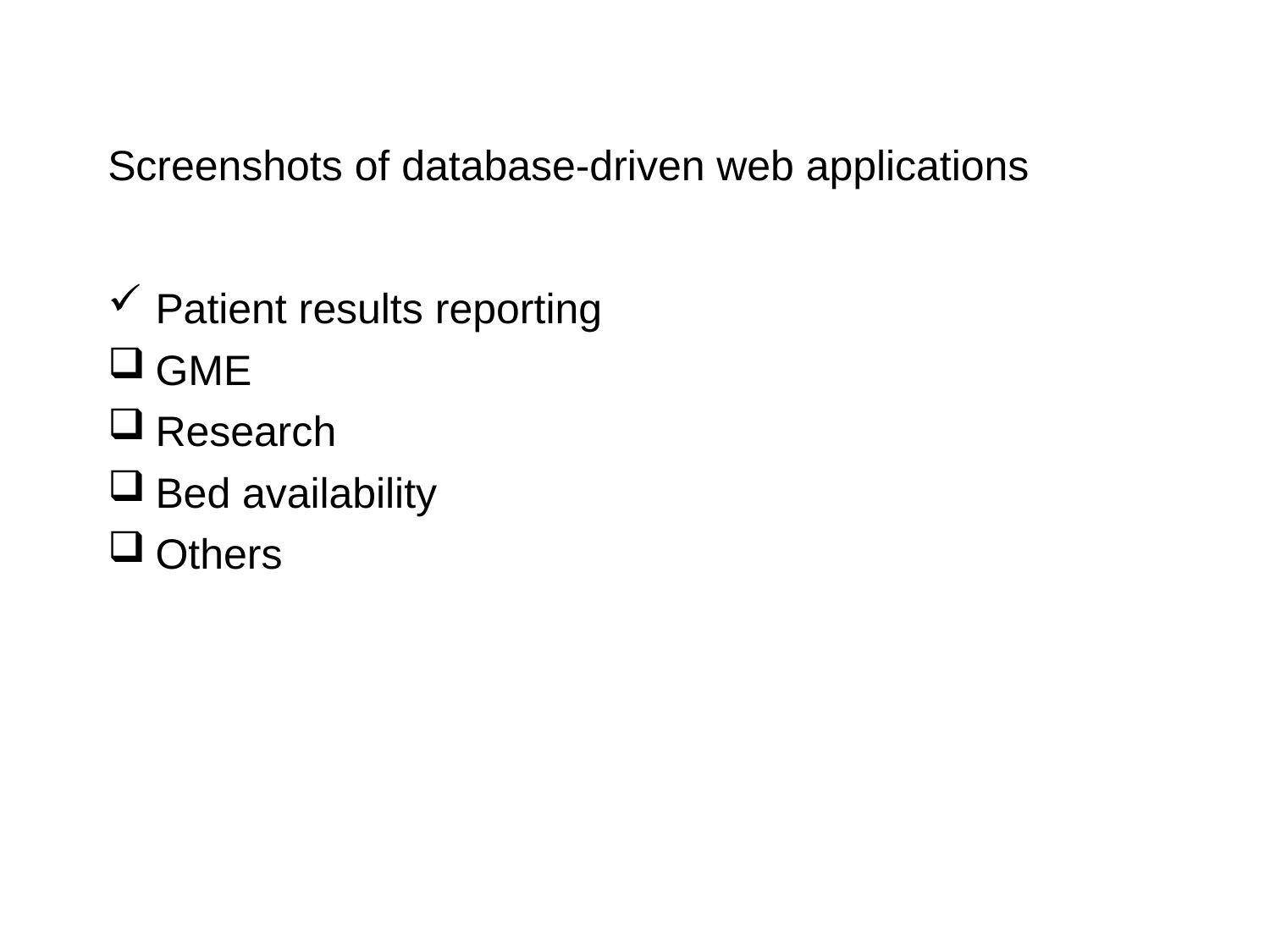

# Screenshots of database-driven web applications
Patient results reporting
GME
Research
Bed availability
Others

## Slide 3
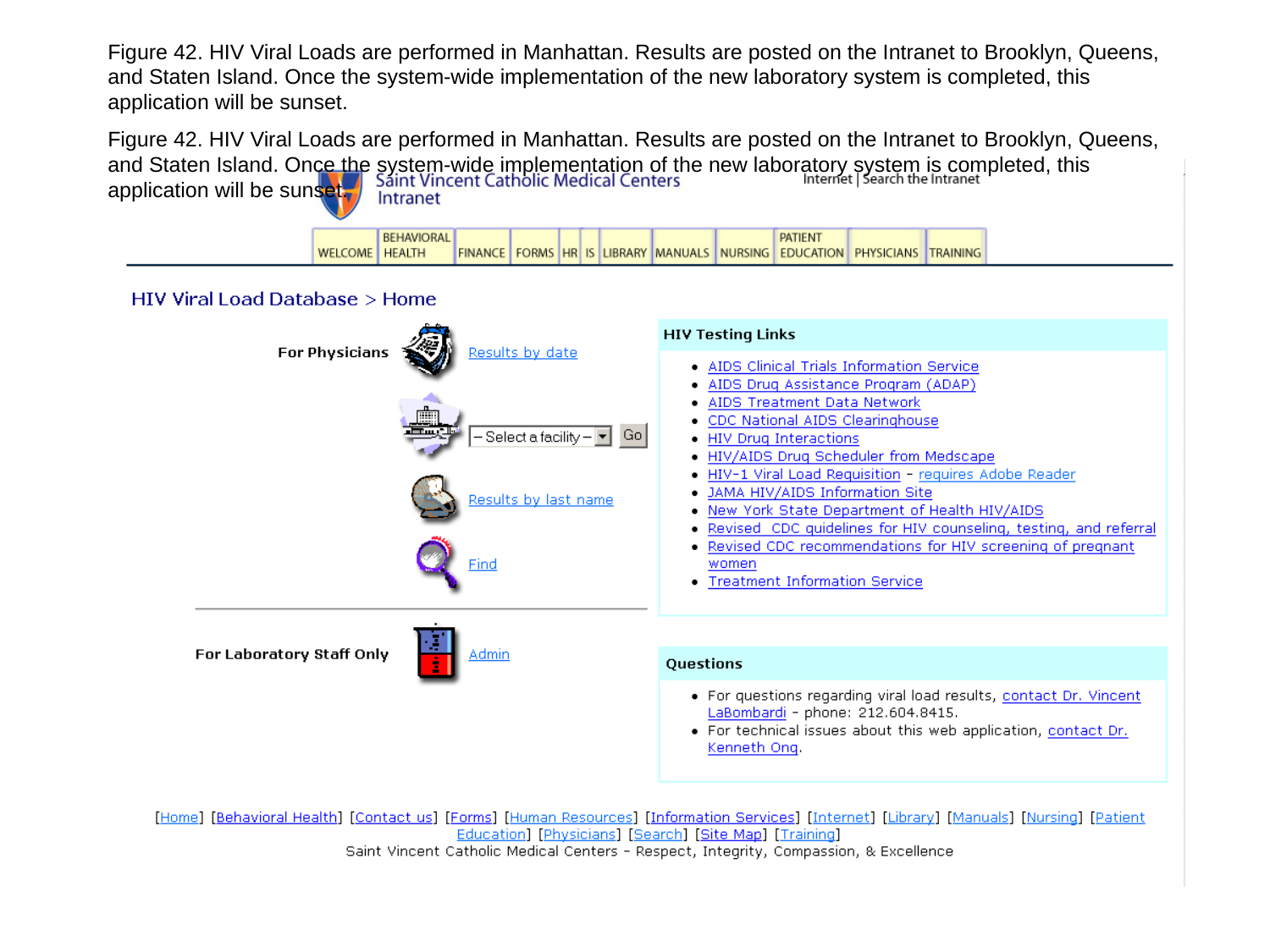

Figure 42. HIV Viral Loads are performed in Manhattan. Results are posted on the Intranet to Brooklyn, Queens, and Staten Island. Once the system-wide implementation of the new laboratory system is completed, this application will be sunset.
# Figure 42. HIV Viral Loads are performed in Manhattan. Results are posted on the Intranet to Brooklyn, Queens, and Staten Island. Once the system-wide implementation of the new laboratory system is completed, this application will be sunset.

## Slide 4
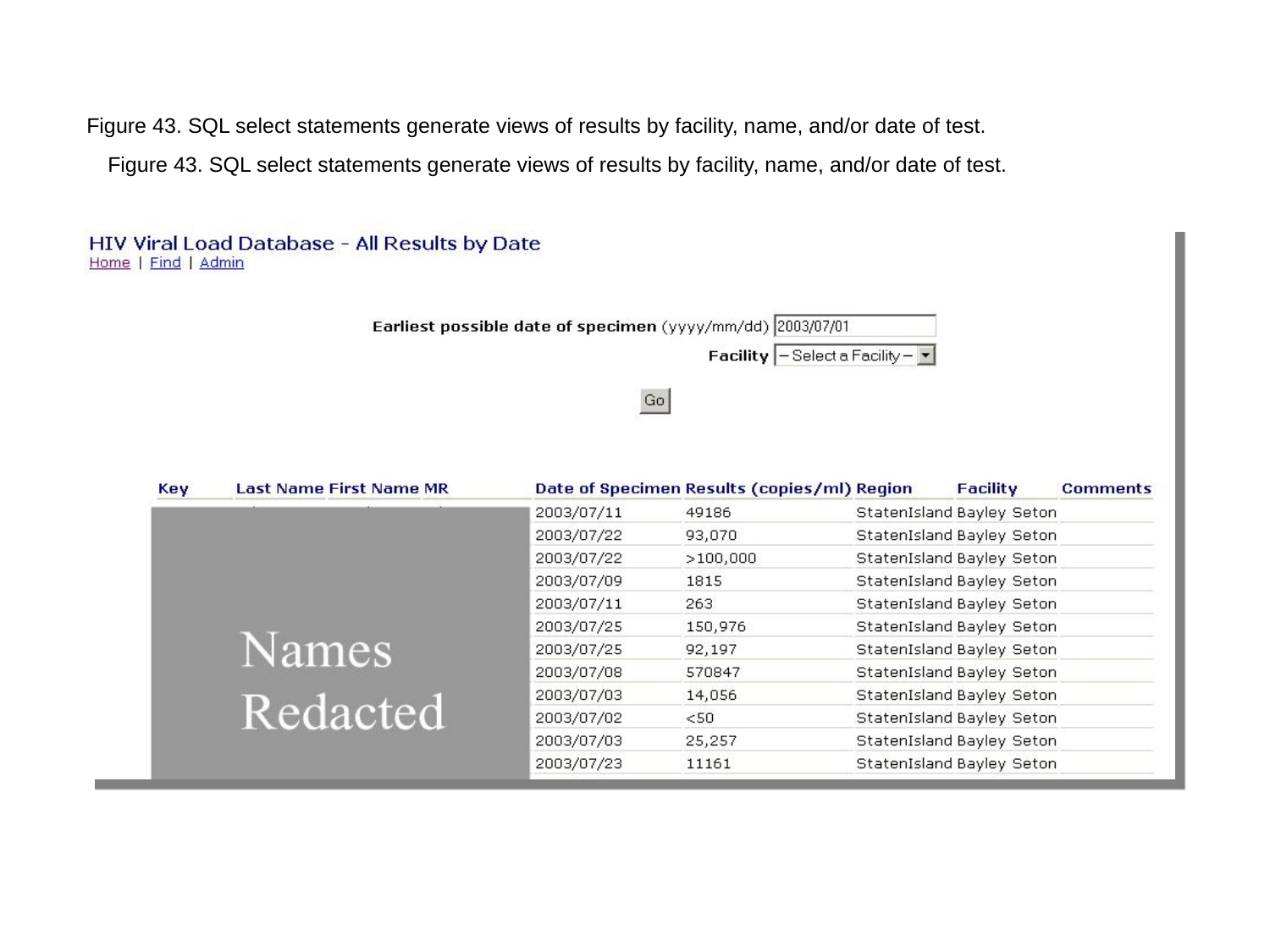

# Figure 43. SQL select statements generate views of results by facility, name, and/or date of test.
Figure 43. SQL select statements generate views of results by facility, name, and/or date of test.

## Slide 5
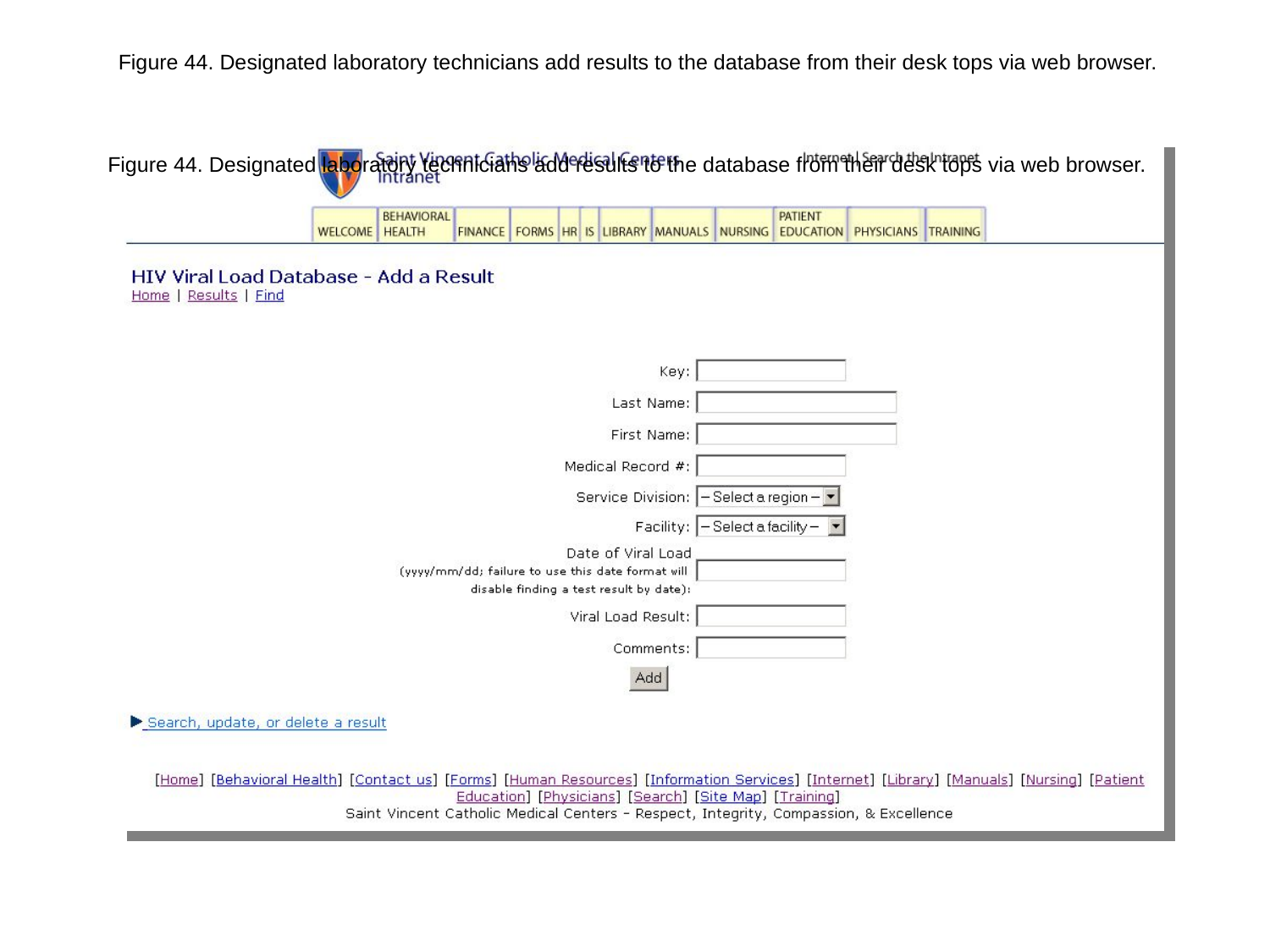

Figure 44. Designated laboratory technicians add results to the database from their desk tops via web browser.
# Figure 44. Designated laboratory technicians add results to the database from their desk tops via web browser.

## Slide 6
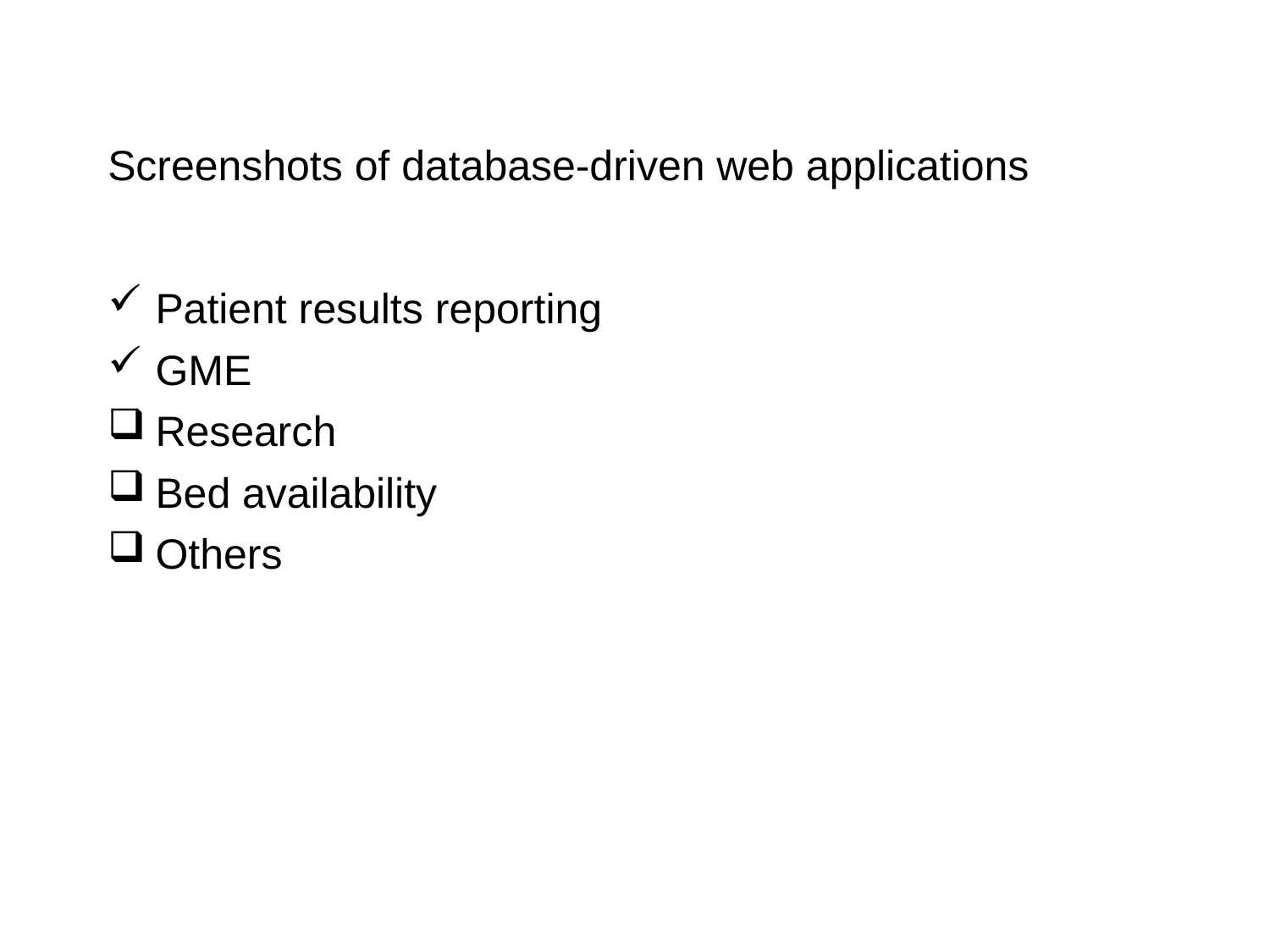

# Screenshots of database-driven web applications
Patient results reporting
GME
Research
Bed availability
Others

## Slide 7
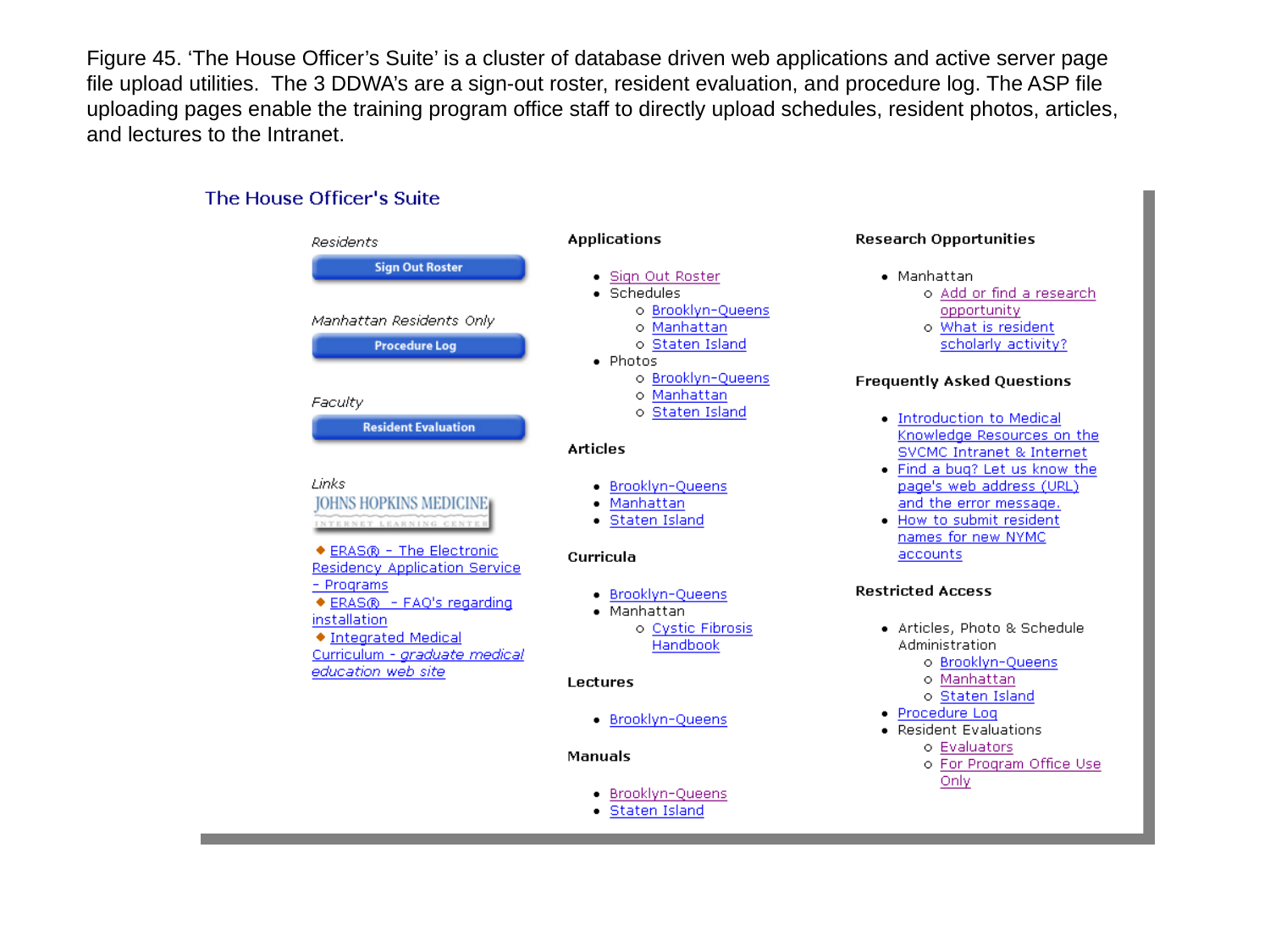

# Figure 45. ‘The House Officer’s Suite’ is a cluster of database driven web applications and active server page file upload utilities. The 3 DDWA’s are a sign-out roster, resident evaluation, and procedure log. The ASP file uploading pages enable the training program office staff to directly upload schedules, resident photos, articles, and lectures to the Intranet.

## Slide 8
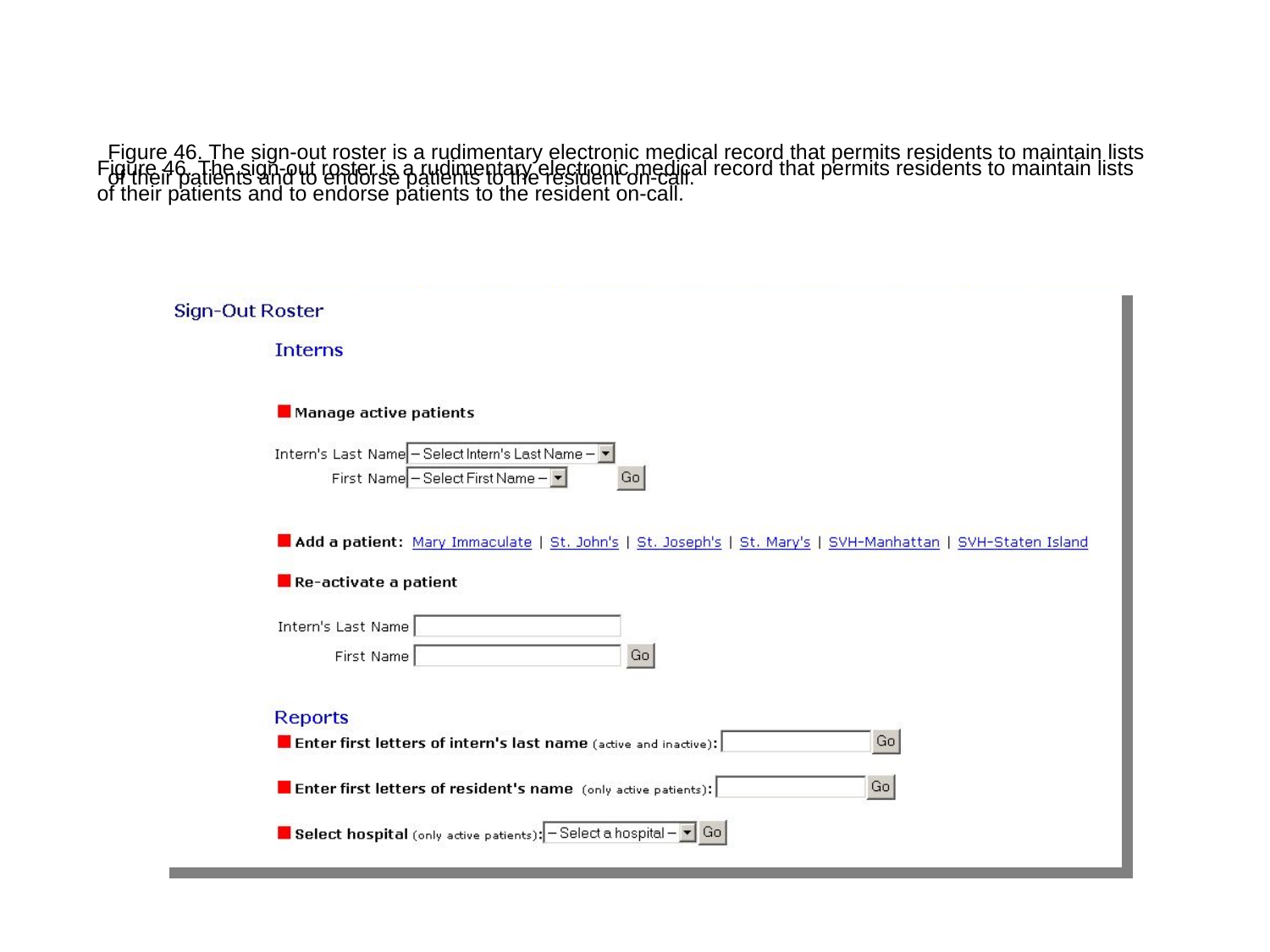

# Figure 46. The sign-out roster is a rudimentary electronic medical record that permits residents to maintain lists of their patients and to endorse patients to the resident on-call.
Figure 46. The sign-out roster is a rudimentary electronic medical record that permits residents to maintain lists of their patients and to endorse patients to the resident on-call.

## Slide 9
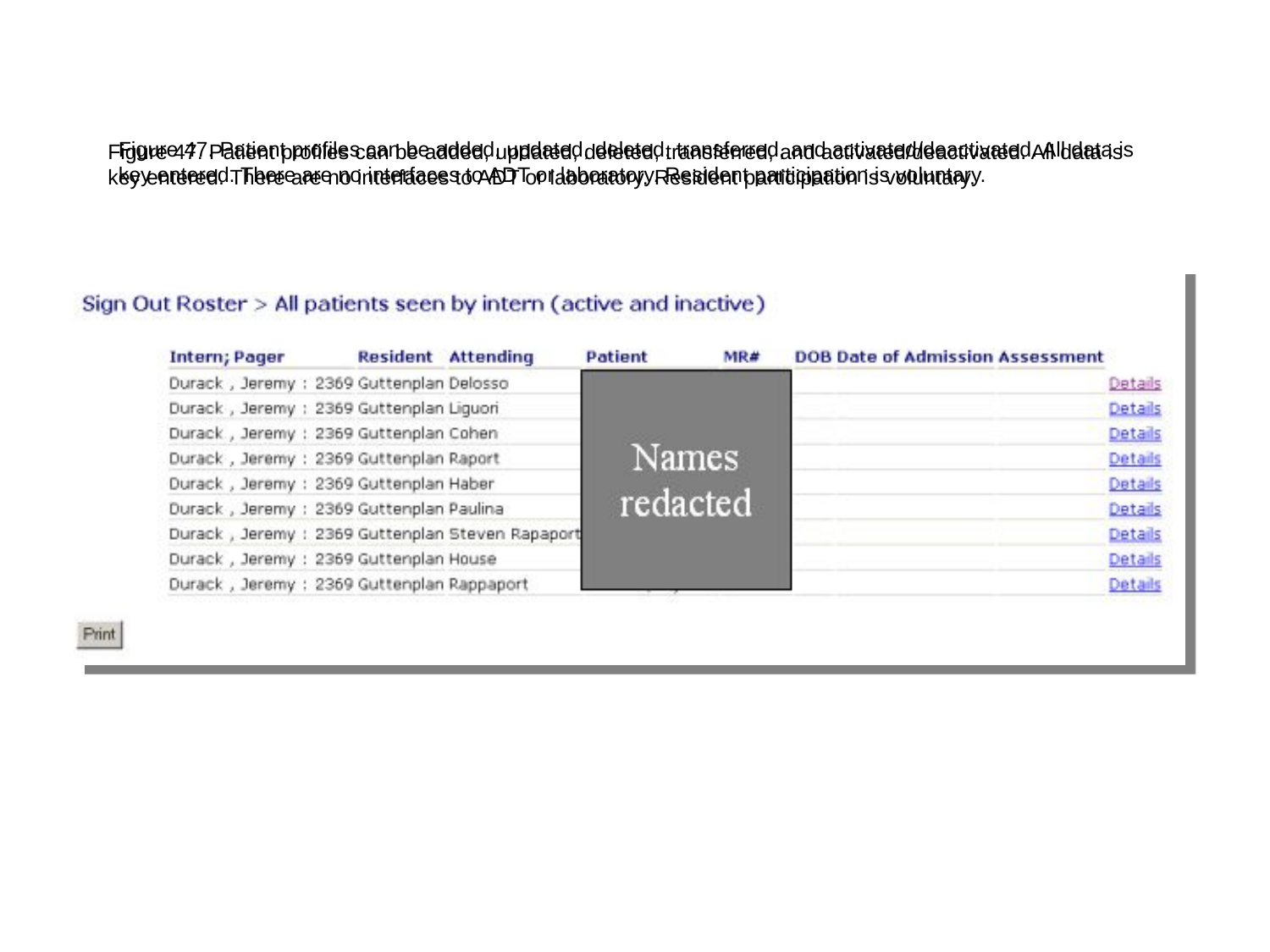

# Figure 47. Patient profiles can be added, updated, deleted, transferred, and activated/deactivated. All data is key entered. There are no interfaces to ADT or laboratory. Resident participation is voluntary.
Figure 47. Patient profiles can be added, updated, deleted, transferred, and activated/deactivated. All data is key entered. There are no interfaces to ADT or laboratory. Resident participation is voluntary.

## Slide 10
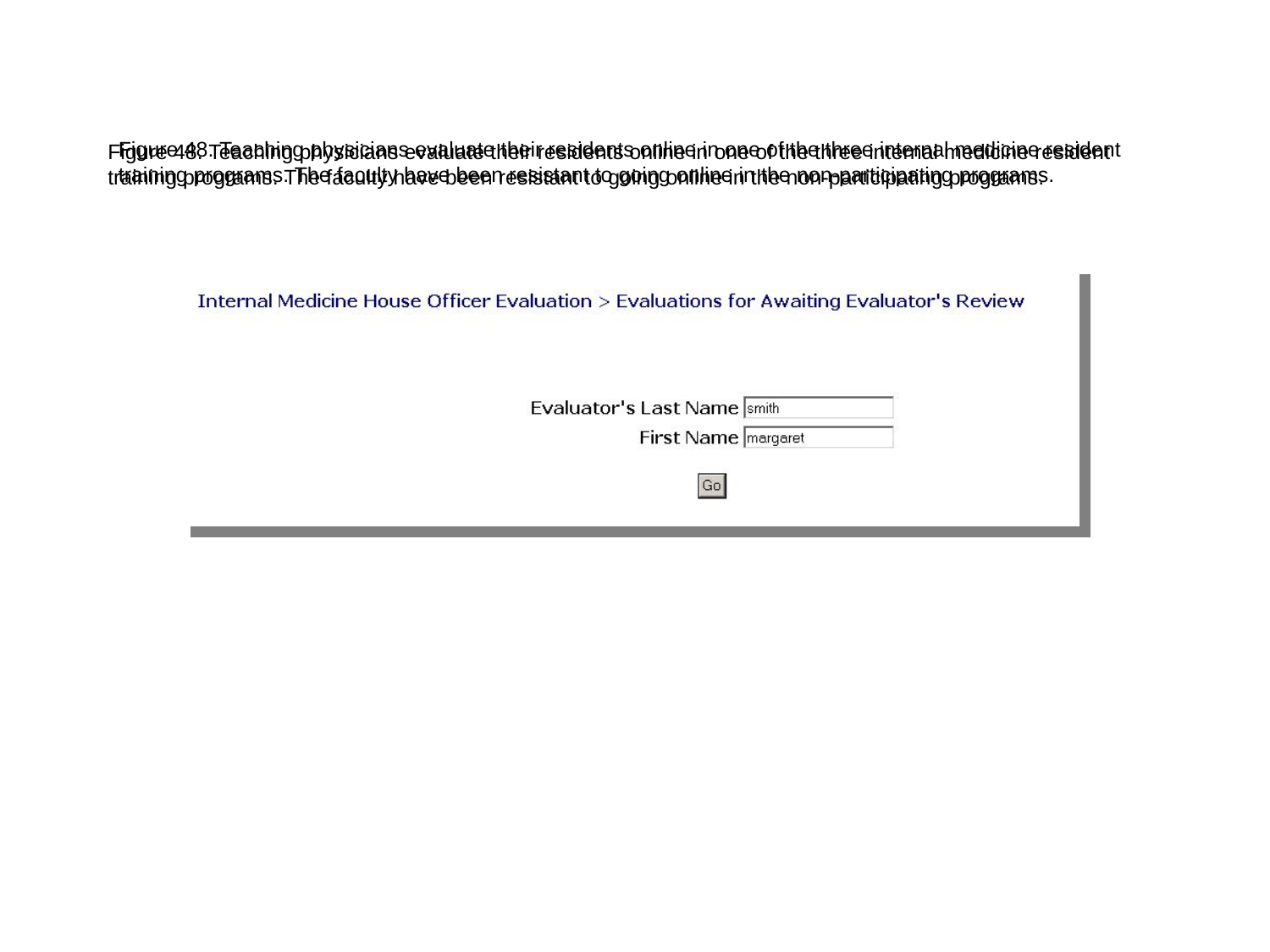

# Figure 48. Teaching physicians evaluate their residents online in one of the three internal medicine resident training programs. The faculty have been resistant to going online in the non-participating programs.
Figure 48. Teaching physicians evaluate their residents online in one of the three internal medicine resident training programs. The faculty have been resistant to going online in the non-participating programs.

## Slide 11
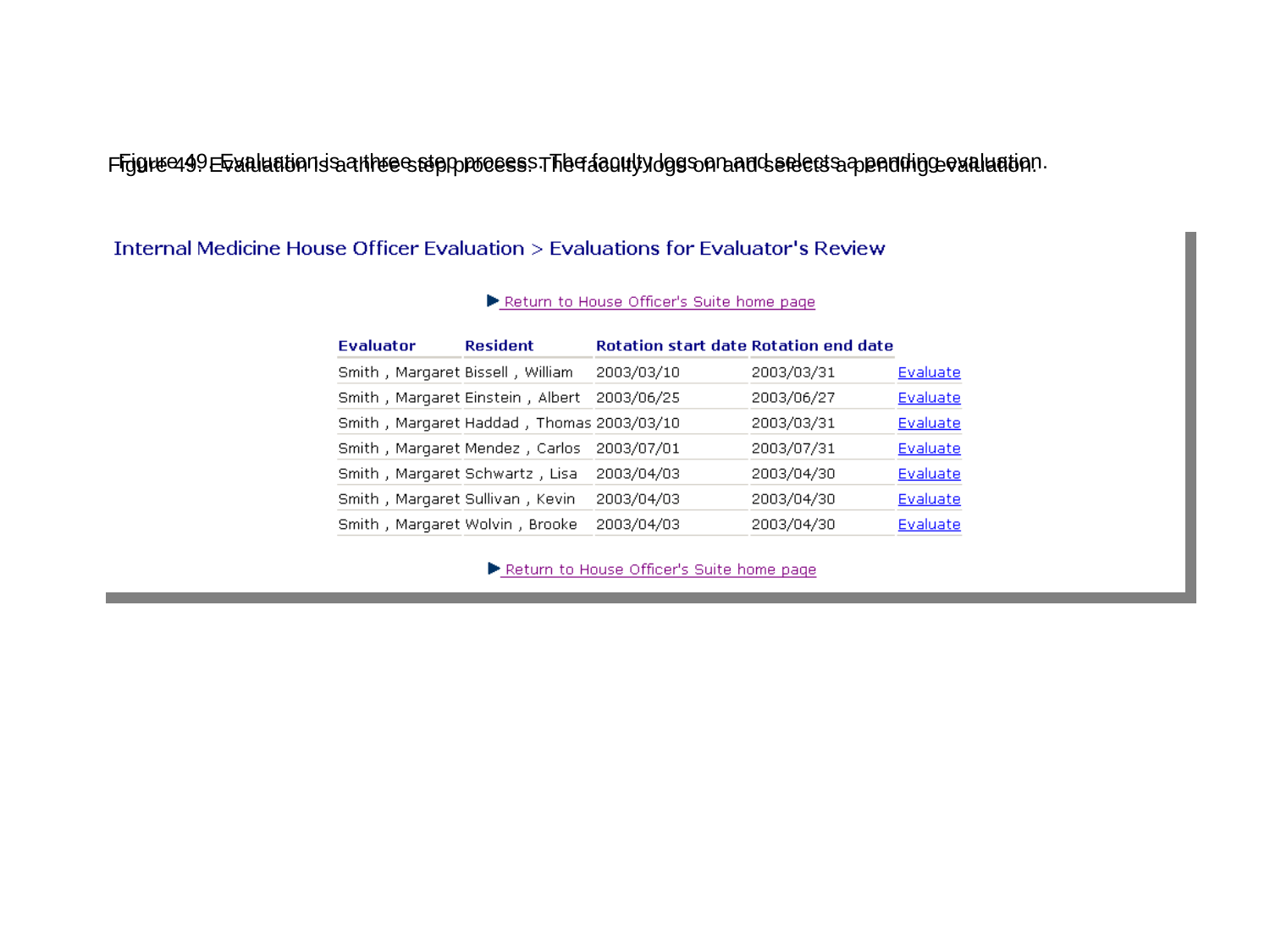

# Figure 49. Evaluation is a three step process. The faculty logs on and selects a pending evaluation.
Figure 49. Evaluation is a three step process. The faculty logs on and selects a pending evaluation.

## Slide 12
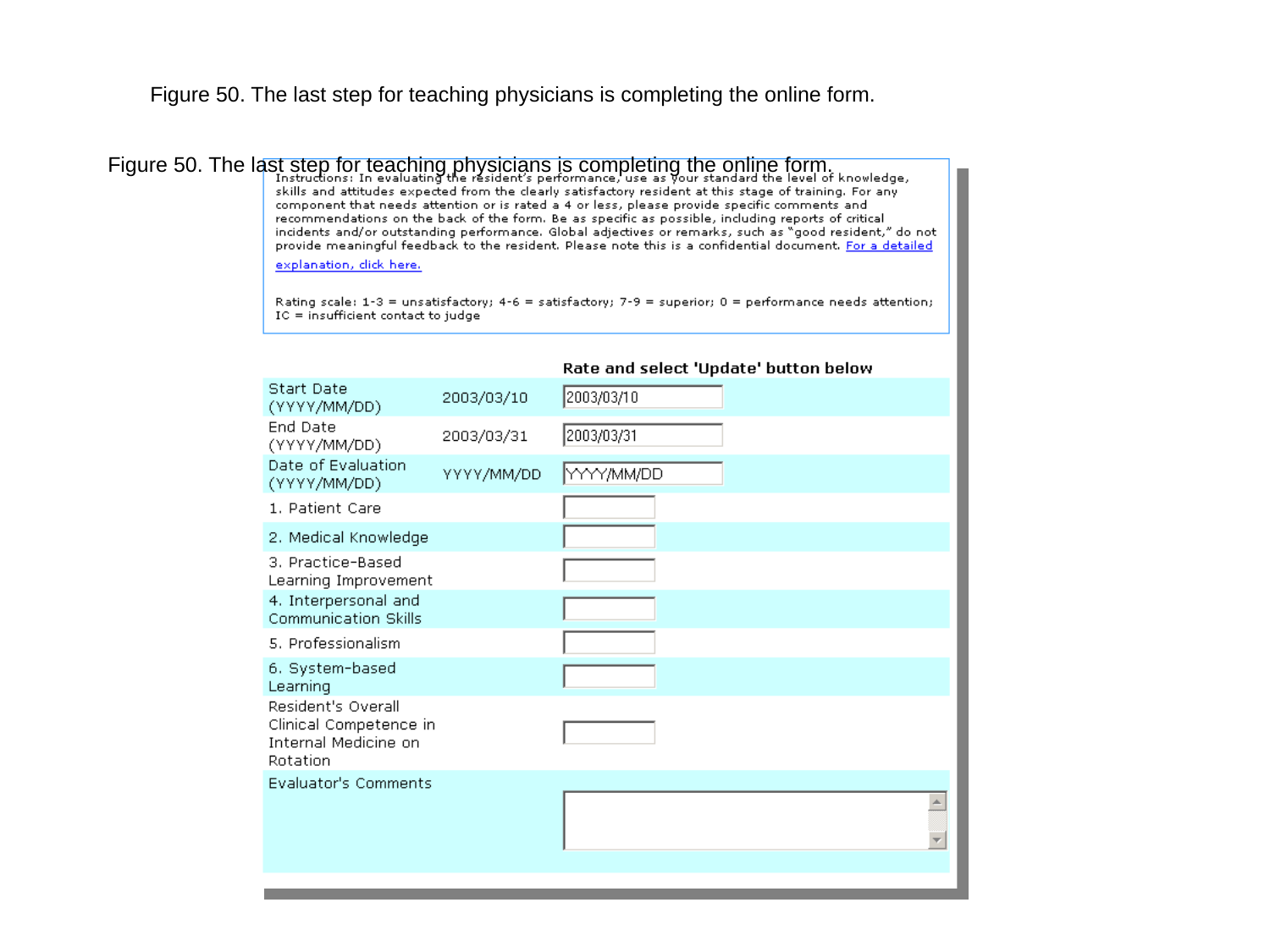

Figure 50. The last step for teaching physicians is completing the online form.
# Figure 50. The last step for teaching physicians is completing the online form.

## Slide 13
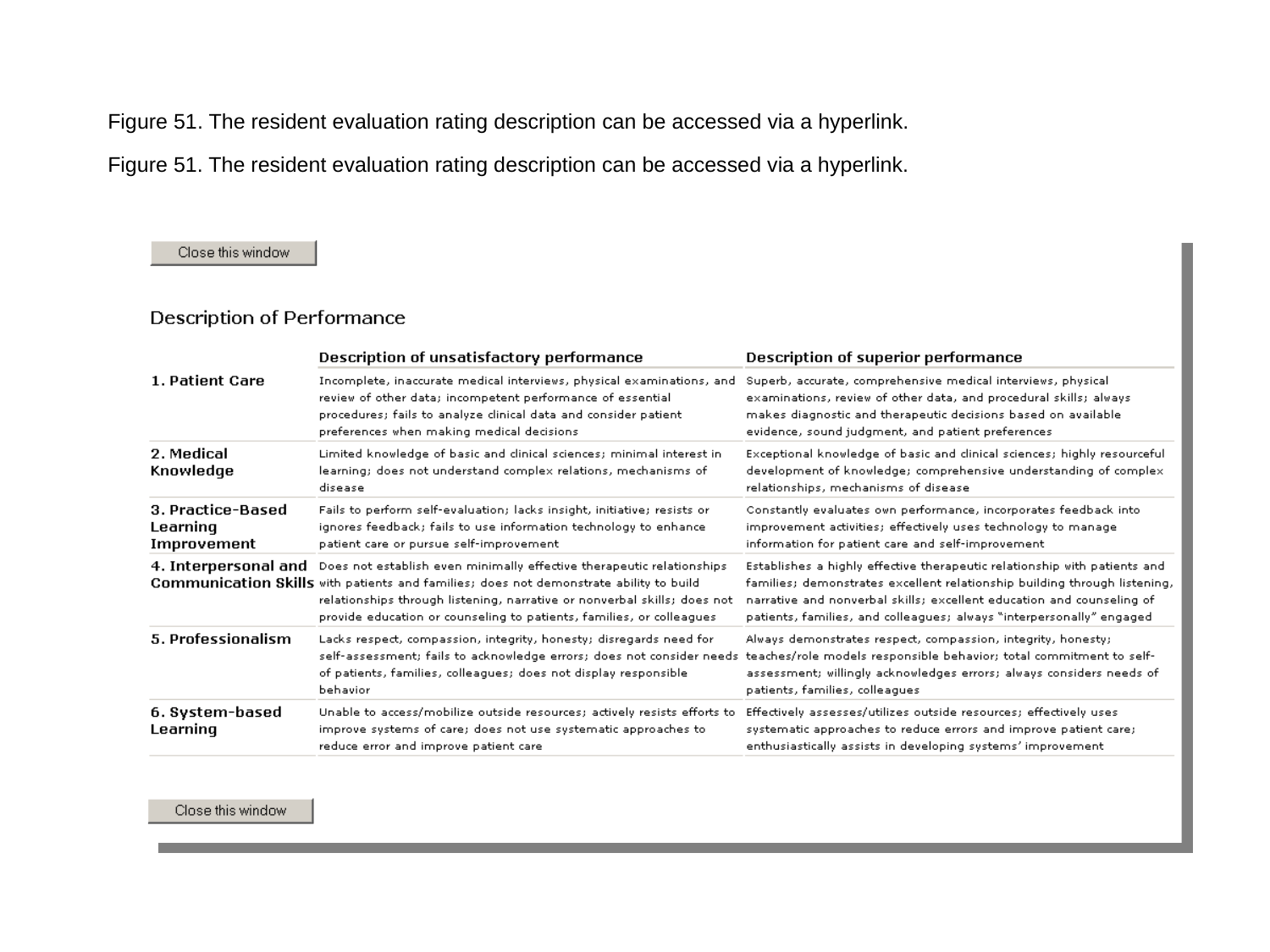

# Figure 51. The resident evaluation rating description can be accessed via a hyperlink.
Figure 51. The resident evaluation rating description can be accessed via a hyperlink.

## Slide 14
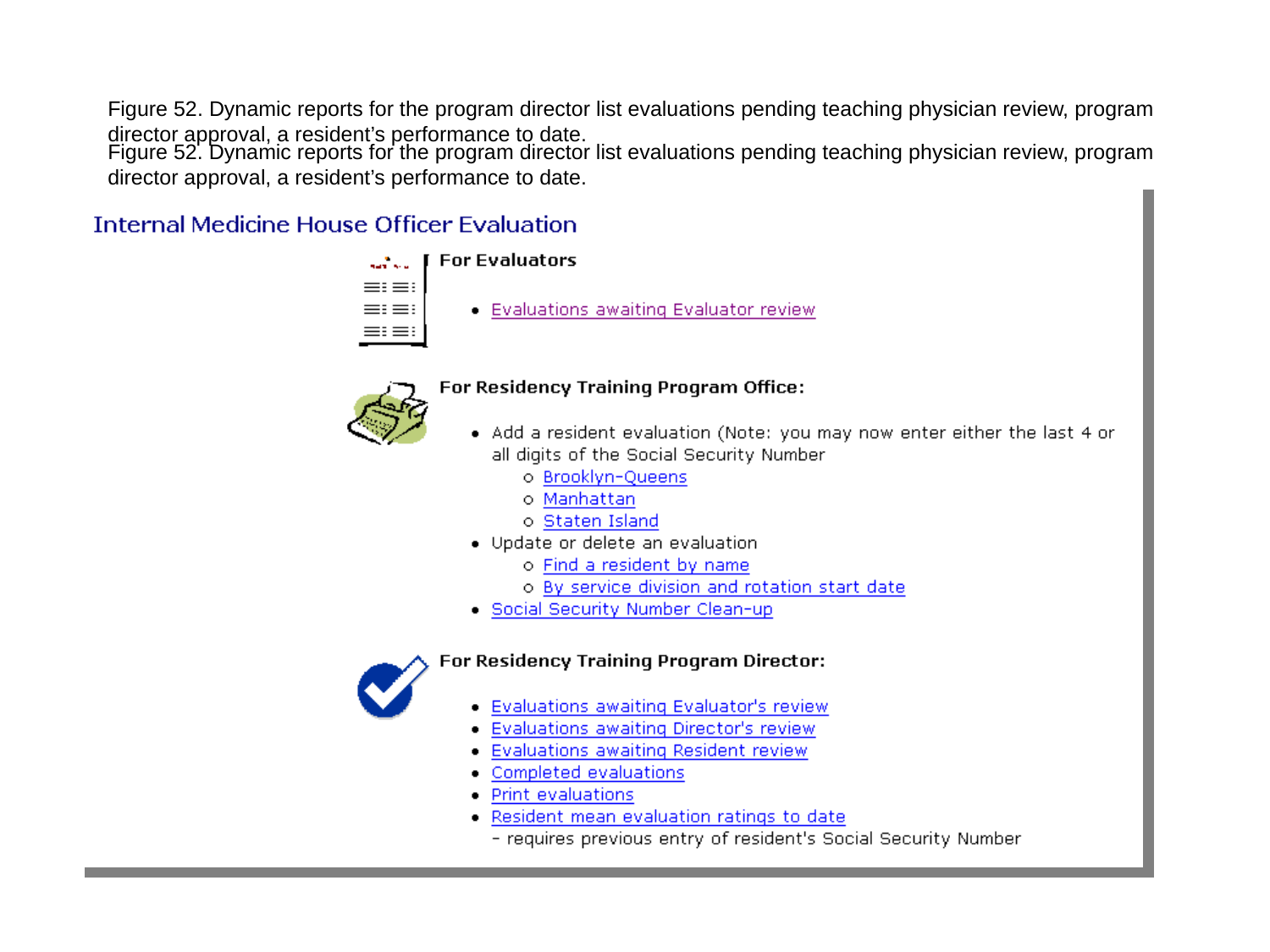

# Figure 52. Dynamic reports for the program director list evaluations pending teaching physician review, program director approval, a resident’s performance to date.
Figure 52. Dynamic reports for the program director list evaluations pending teaching physician review, program director approval, a resident’s performance to date.

## Slide 15
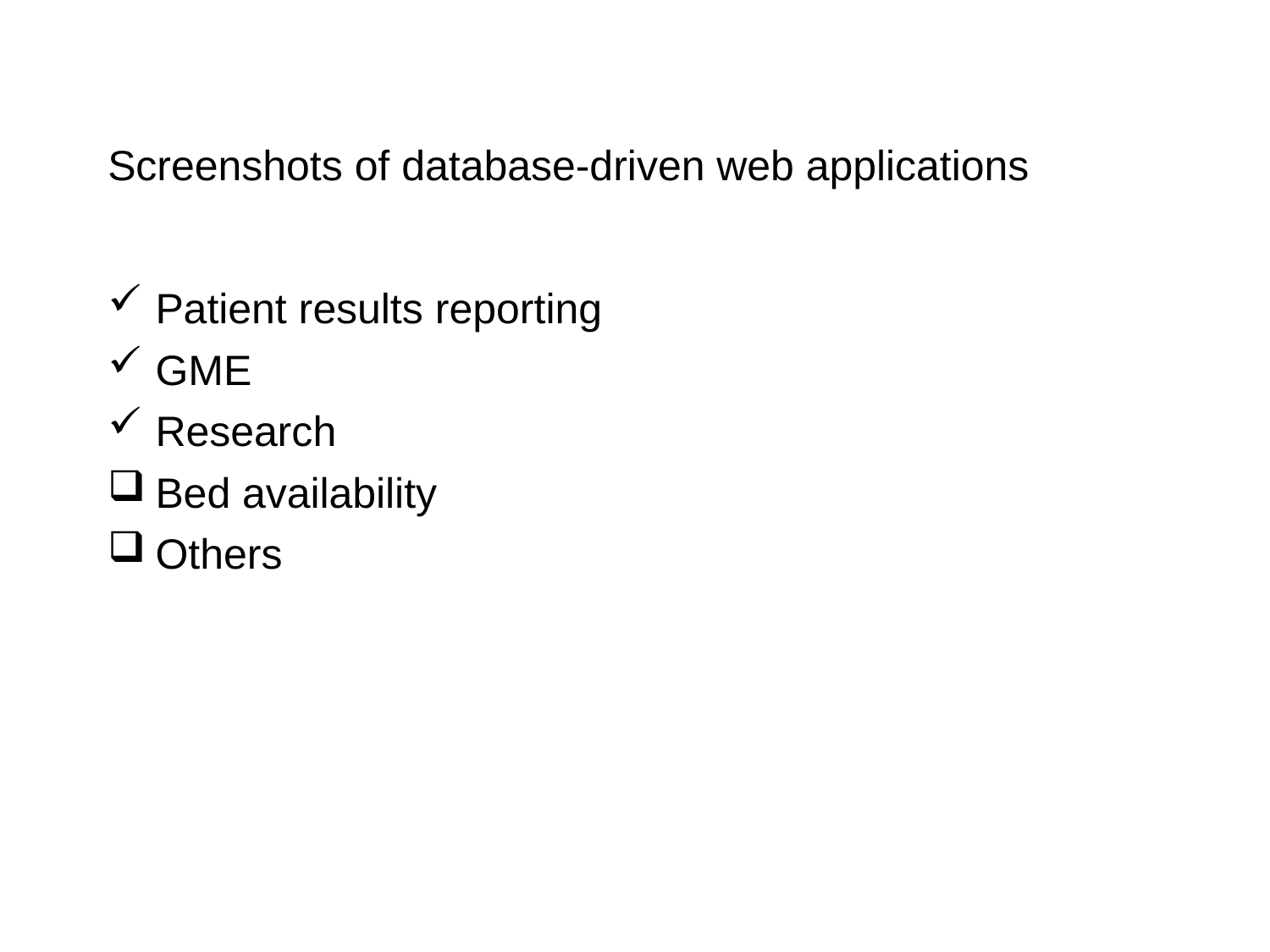

# Screenshots of database-driven web applications
Patient results reporting
GME
Research
Bed availability
Others

## Slide 16
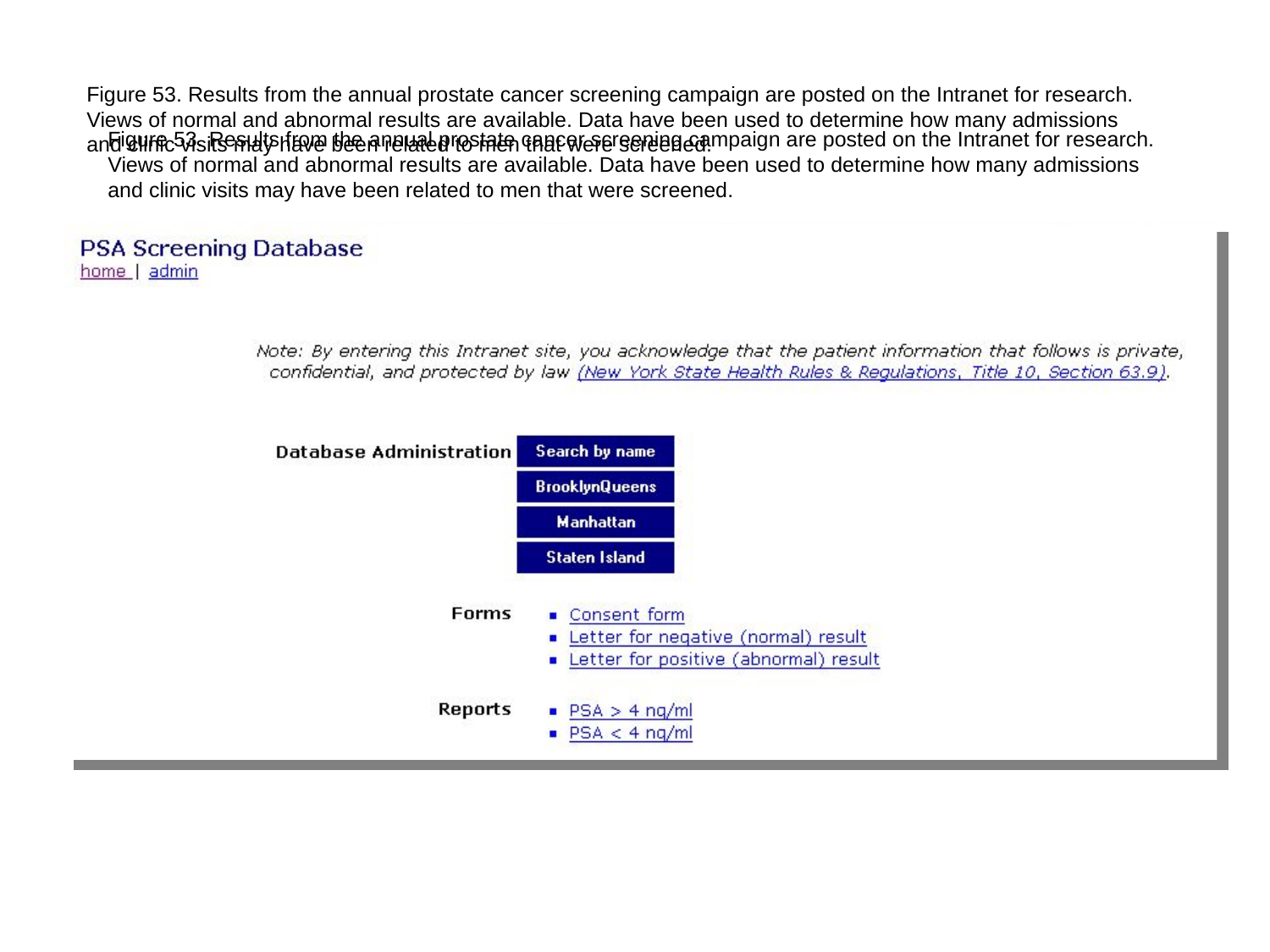

Figure 53. Results from the annual prostate cancer screening campaign are posted on the Intranet for research. Views of normal and abnormal results are available. Data have been used to determine how many admissions and clinic visits may have been related to men that were screened.
# Figure 53. Results from the annual prostate cancer screening campaign are posted on the Intranet for research. Views of normal and abnormal results are available. Data have been used to determine how many admissions and clinic visits may have been related to men that were screened.

## Slide 17
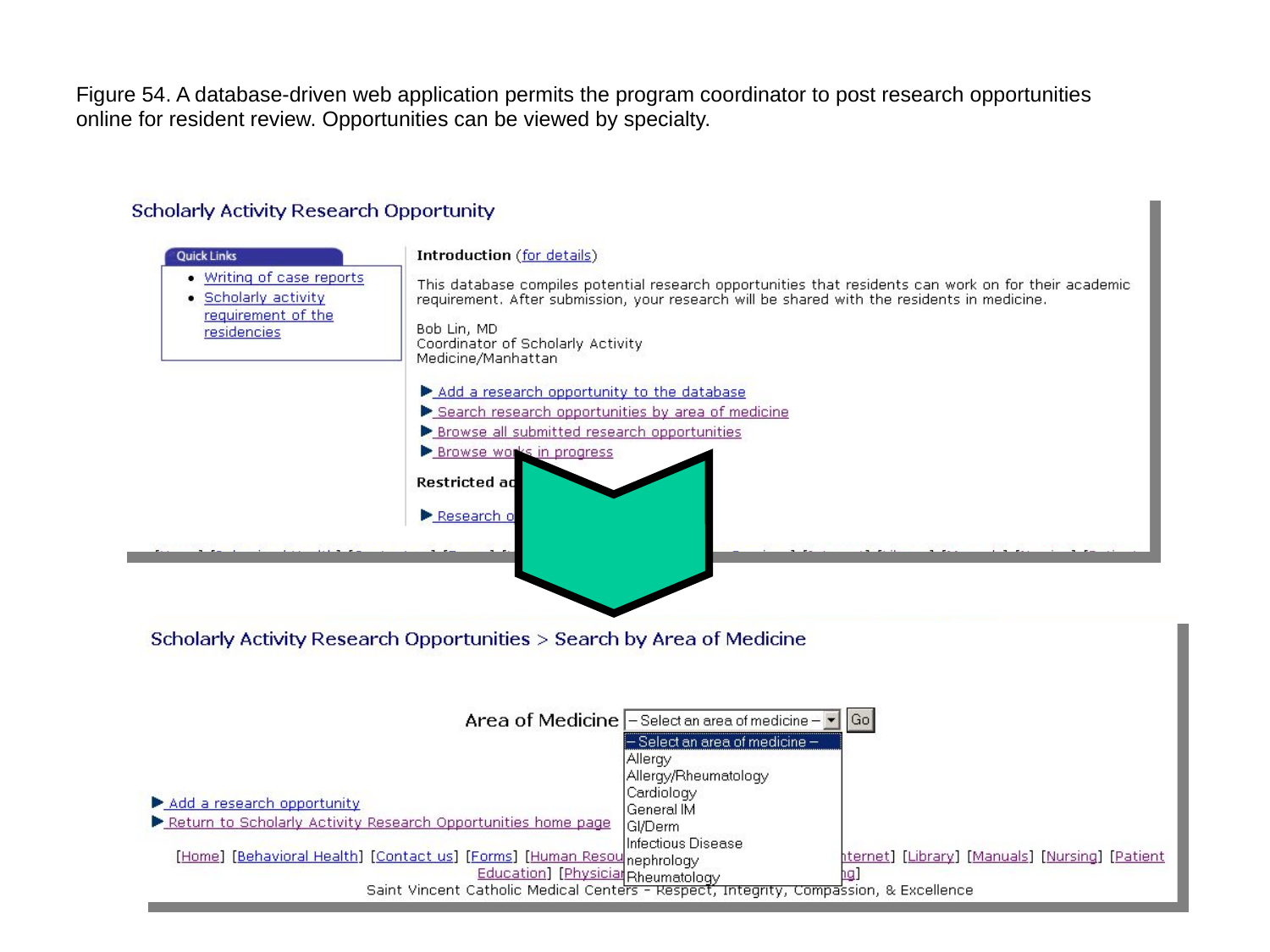

Figure 54. A database-driven web application permits the program coordinator to post research opportunities online for resident review. Opportunities can be viewed by specialty.

## Slide 18
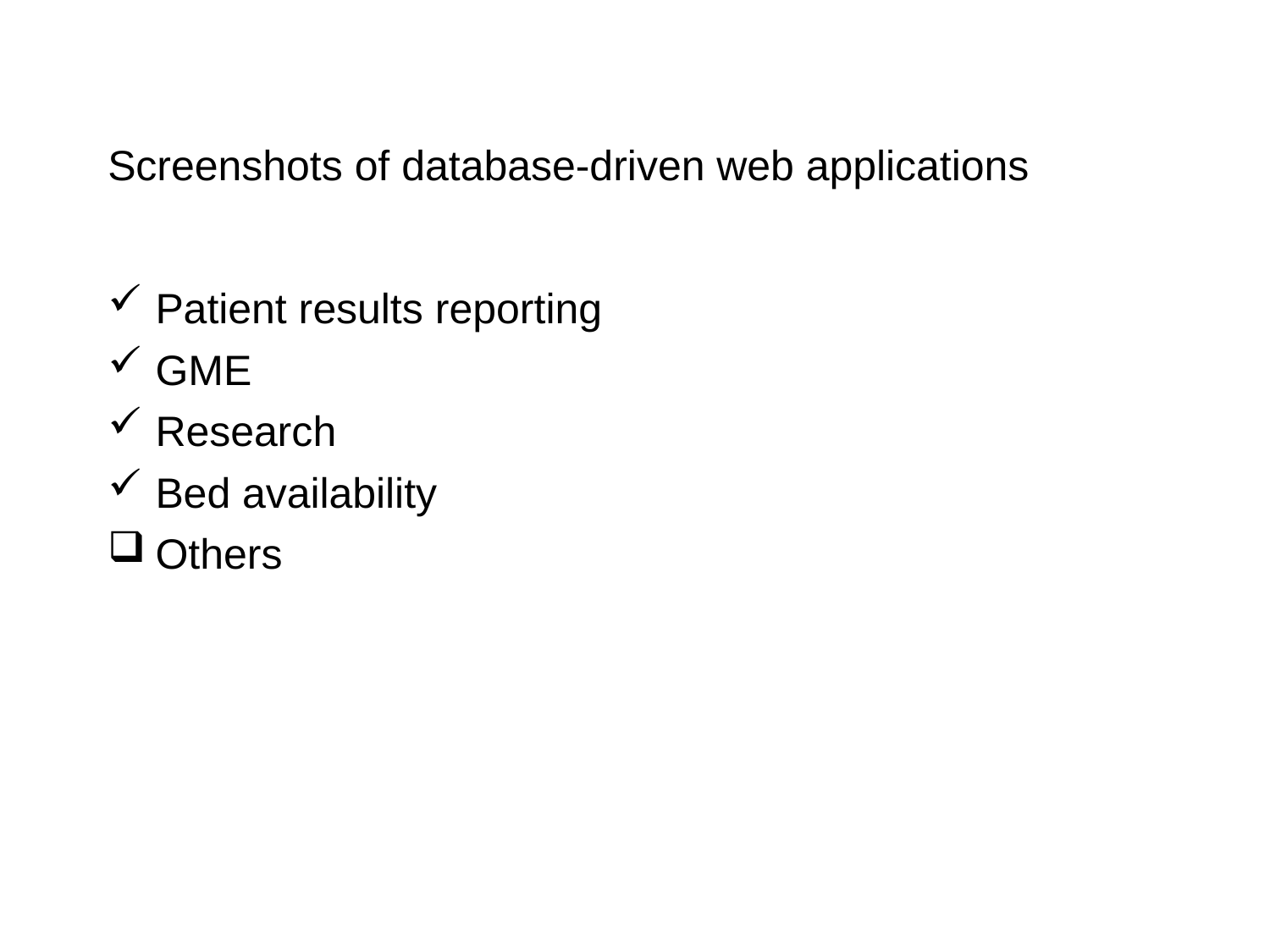

# Screenshots of database-driven web applications
Patient results reporting
GME
Research
Bed availability
Others

## Slide 19
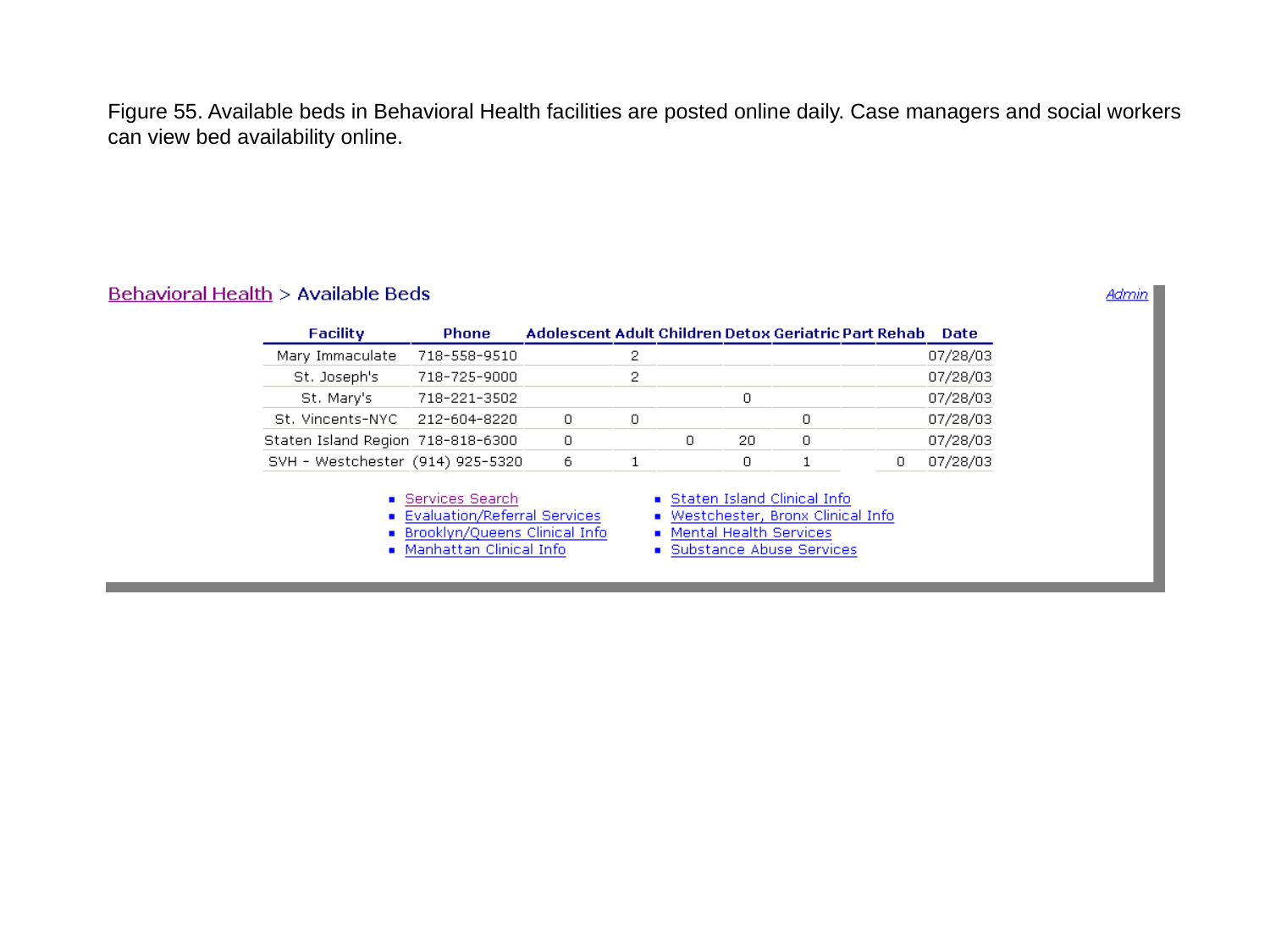

# Figure 55. Available beds in Behavioral Health facilities are posted online daily. Case managers and social workers can view bed availability online.

## Slide 20
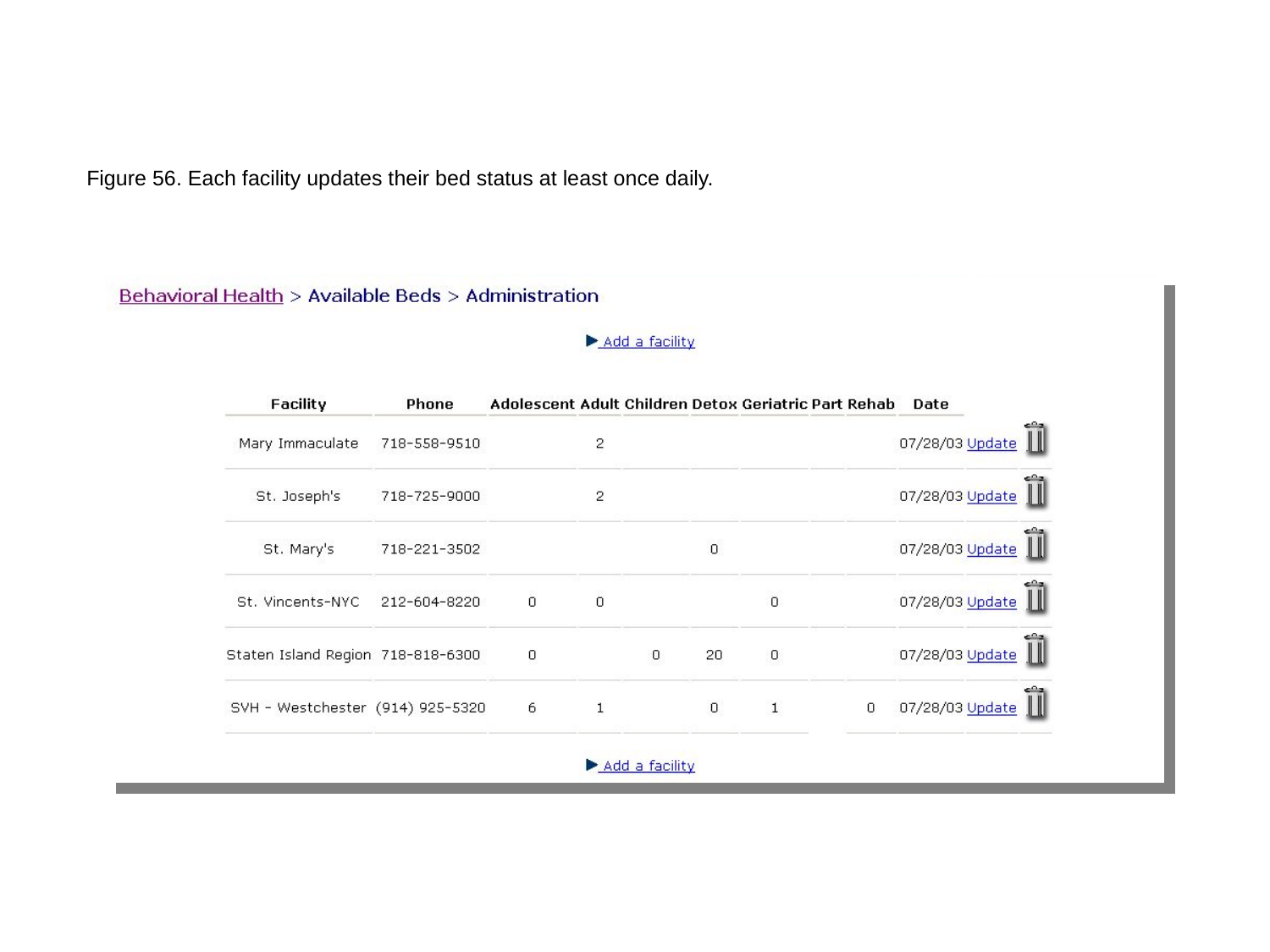

# Figure 56. Each facility updates their bed status at least once daily.

## Slide 21
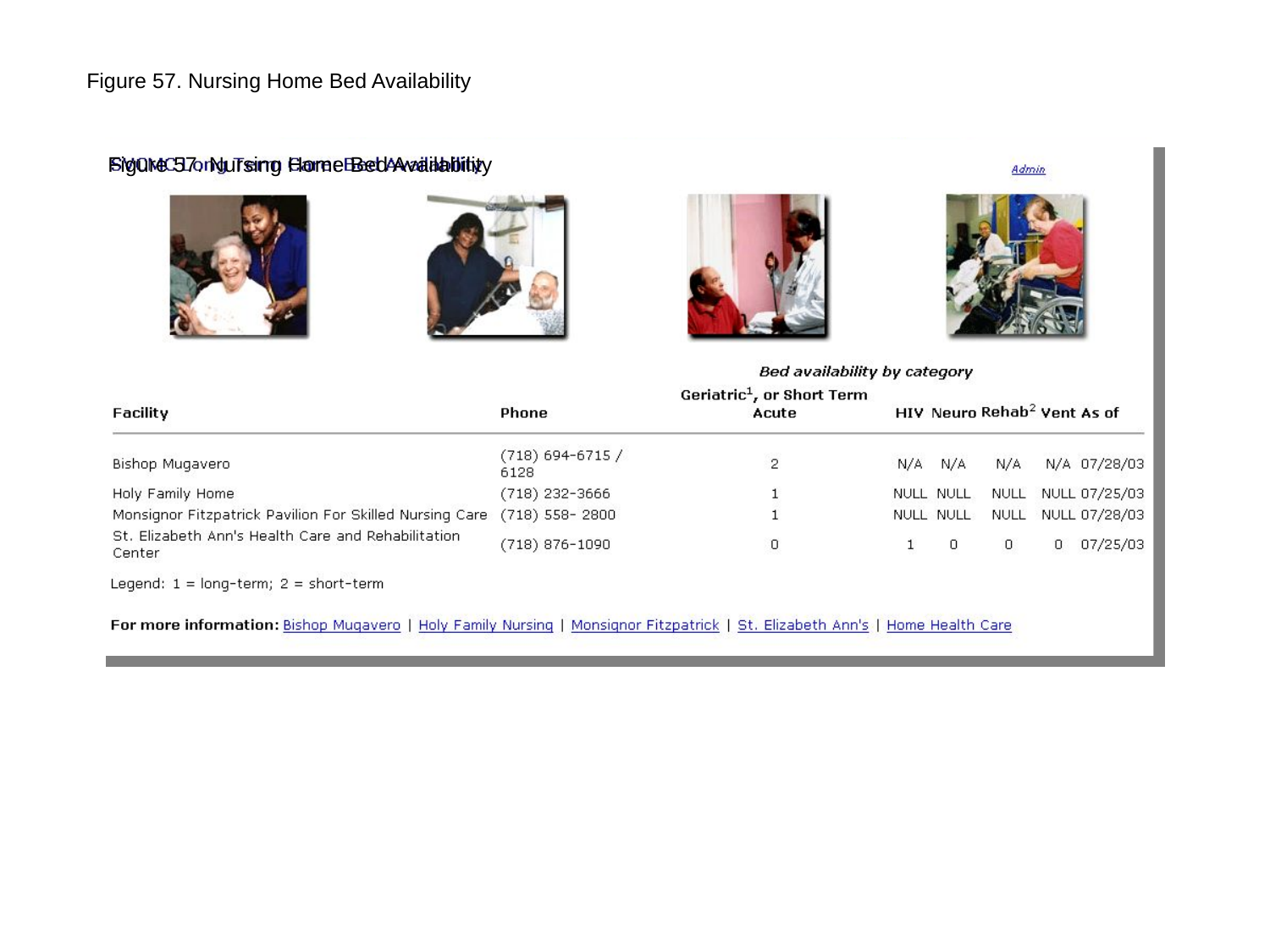

Figure 57. Nursing Home Bed Availability
# Figure 57. Nursing Home Bed Availability

## Slide 22
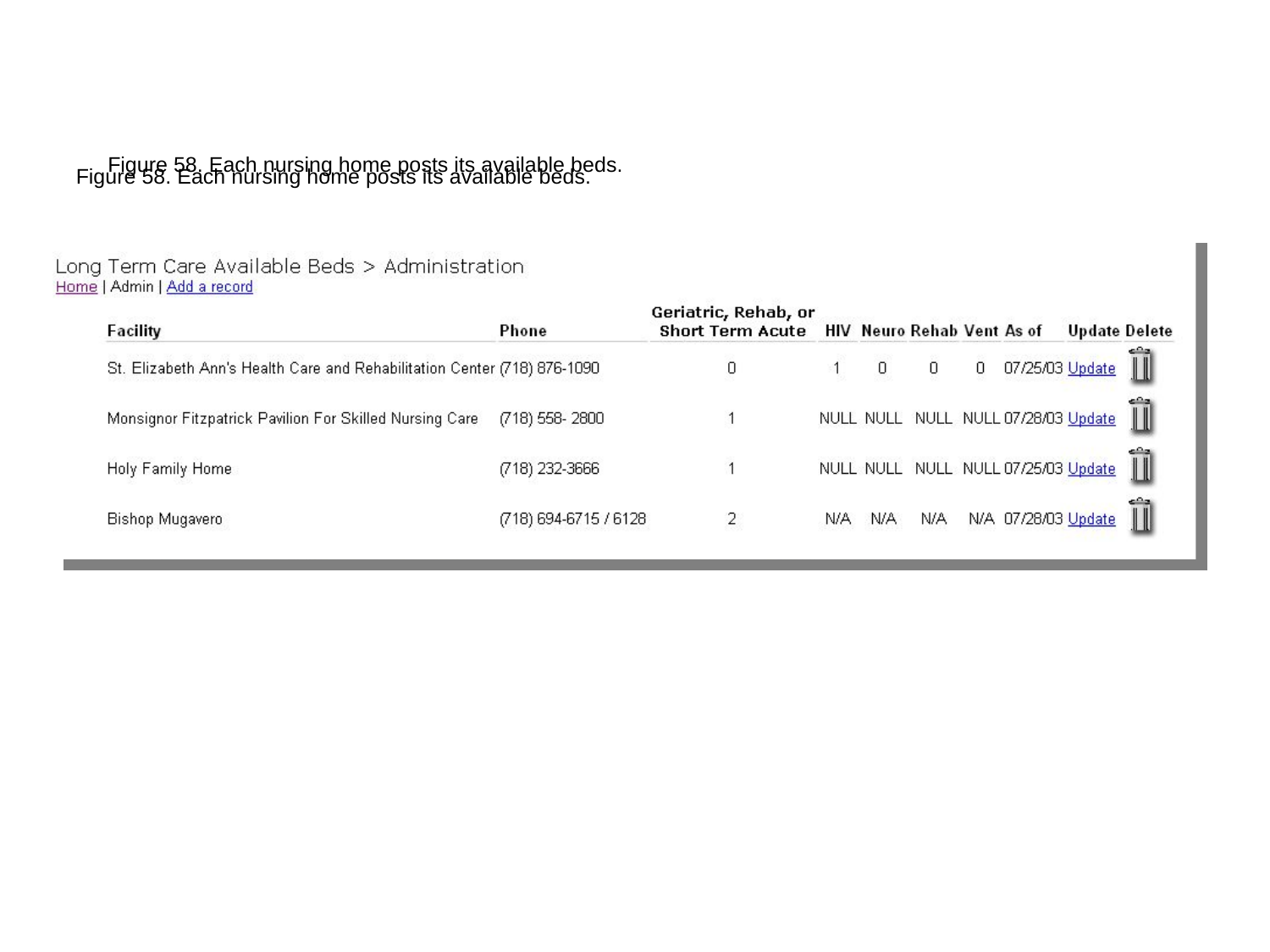

# Figure 58. Each nursing home posts its available beds.
Figure 58. Each nursing home posts its available beds.

## Slide 23
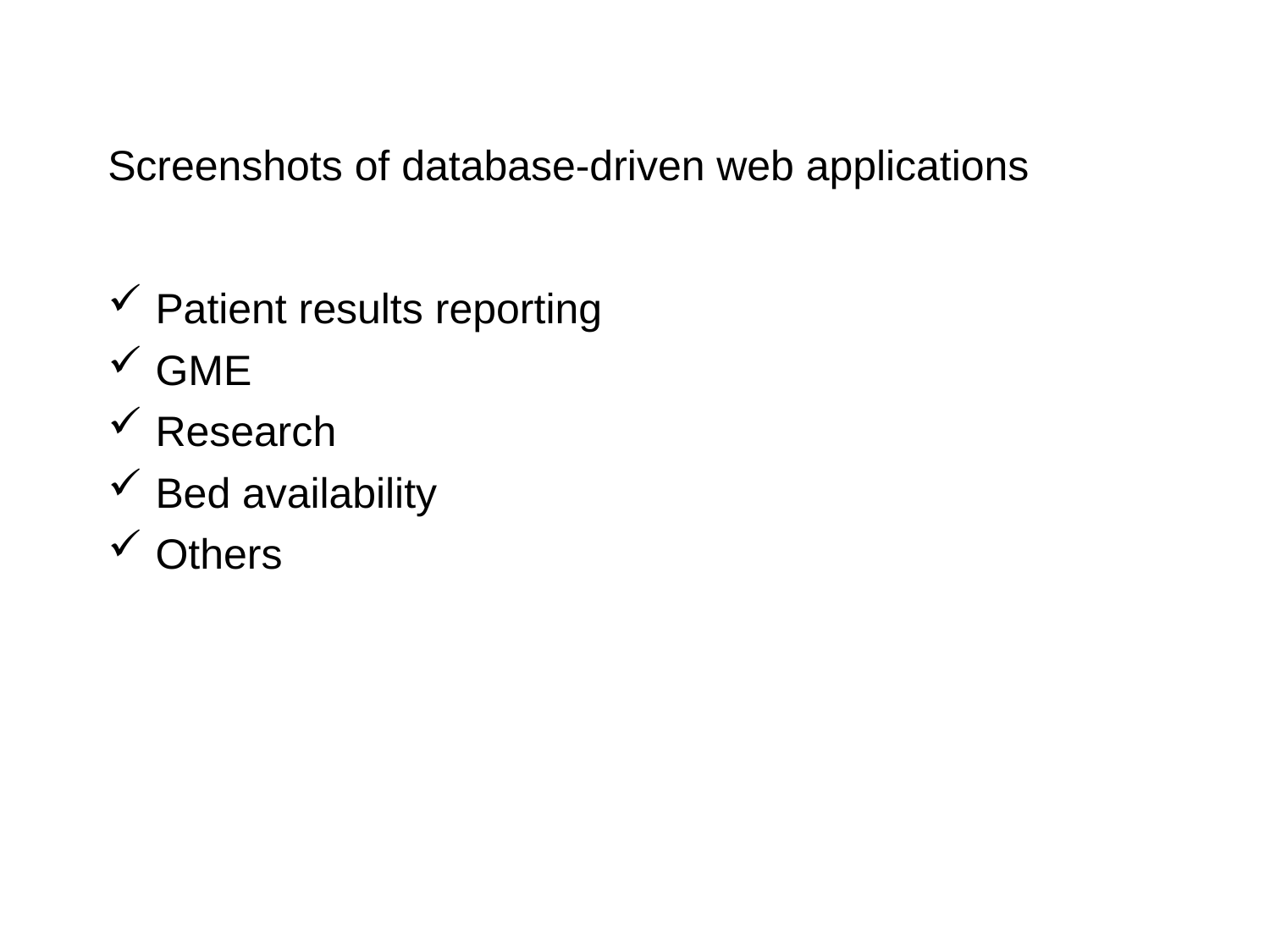

# Screenshots of database-driven web applications
Patient results reporting
GME
Research
Bed availability
Others

## Slide 24
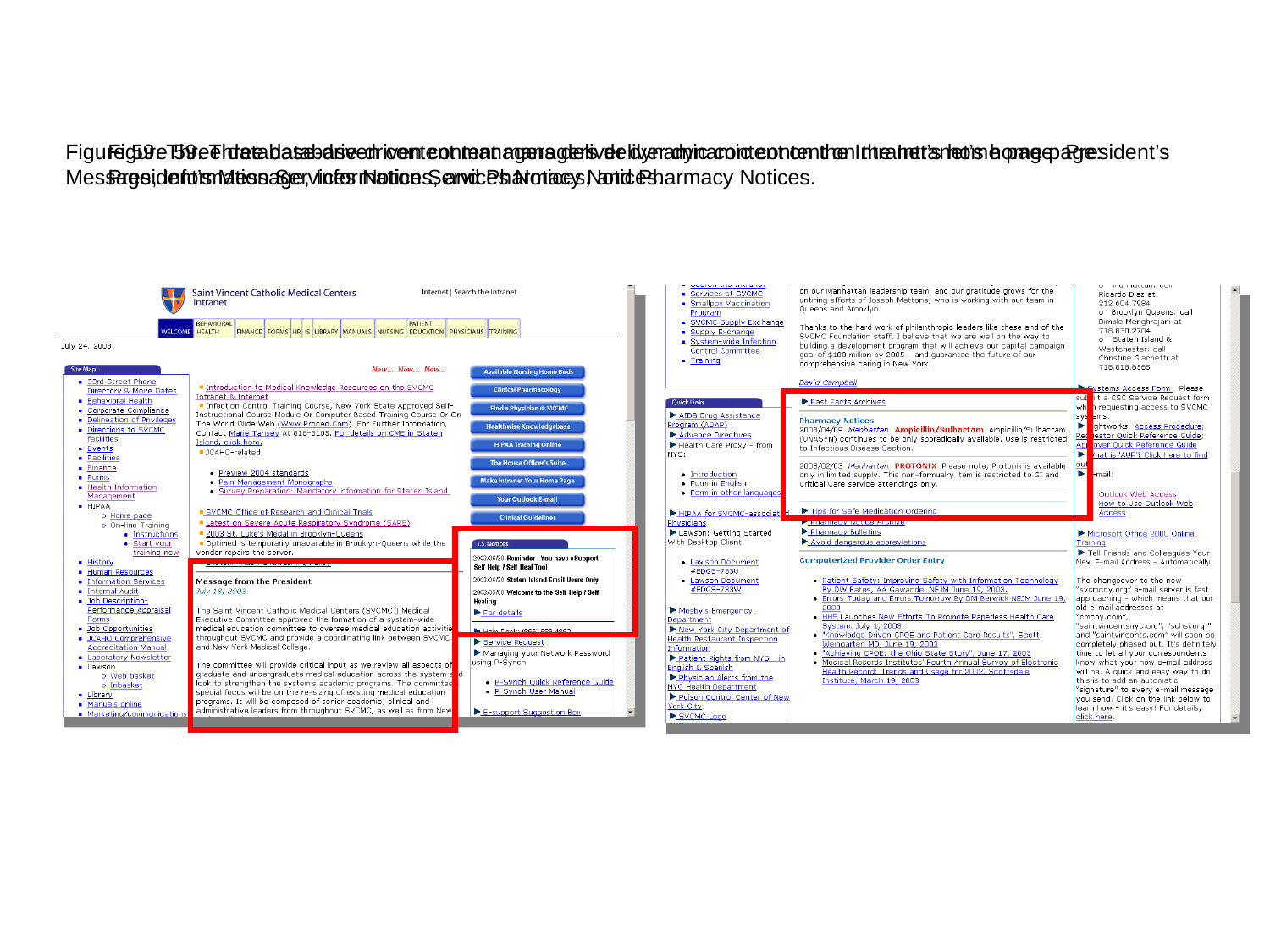

# Figure 59. Three database-driven content managers deliver dynamic content on the Intranet’s home page: President’s Message, Information Services Notices, and Pharmacy Notices.
Figure 59. Three database-driven content managers deliver dynamic content on the Intranet’s home page: President’s Message, Information Services Notices, and Pharmacy Notices.

## Slide 25
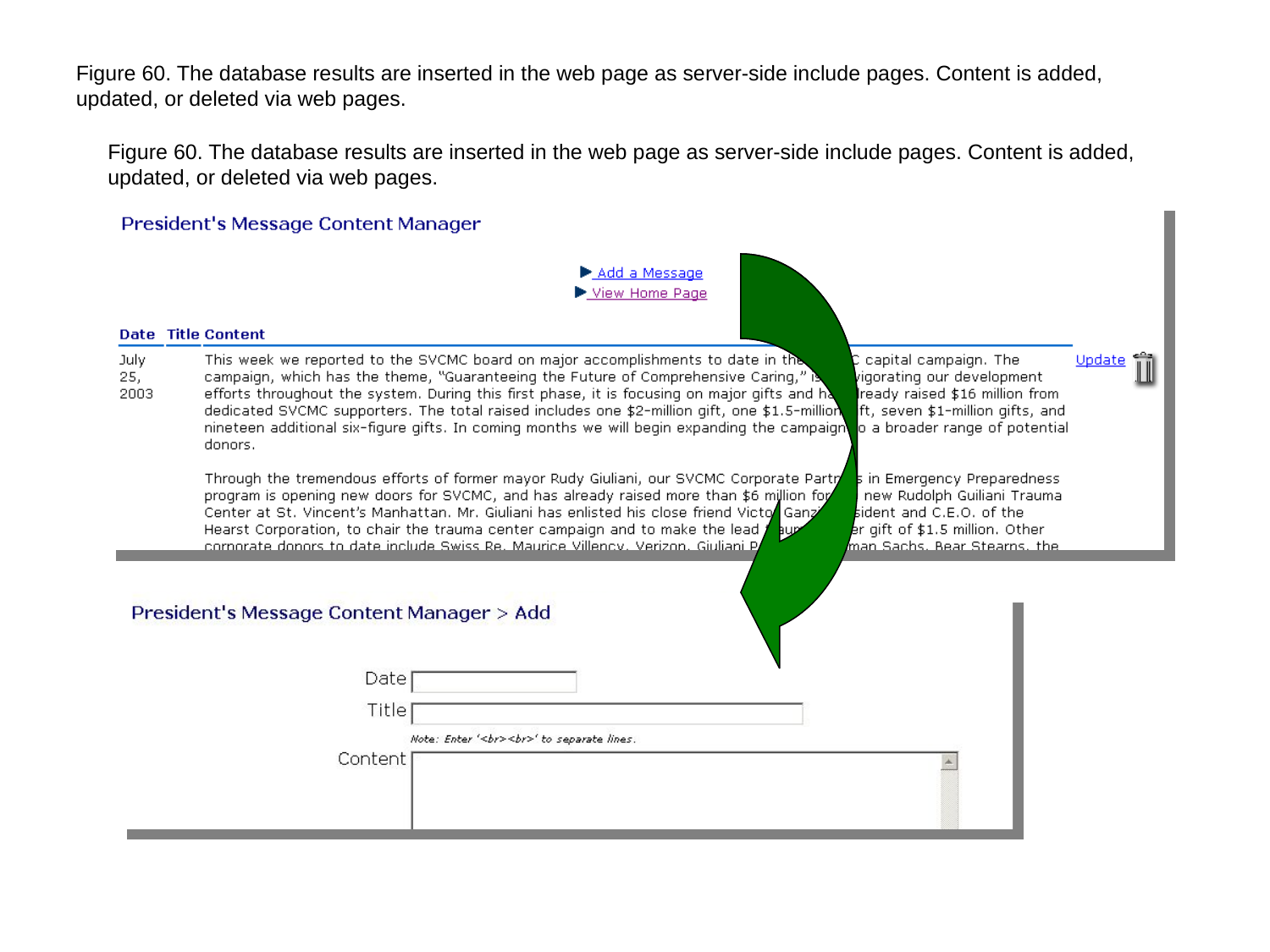

Figure 60. The database results are inserted in the web page as server-side include pages. Content is added, updated, or deleted via web pages.
# Figure 60. The database results are inserted in the web page as server-side include pages. Content is added, updated, or deleted via web pages.

## Slide 26
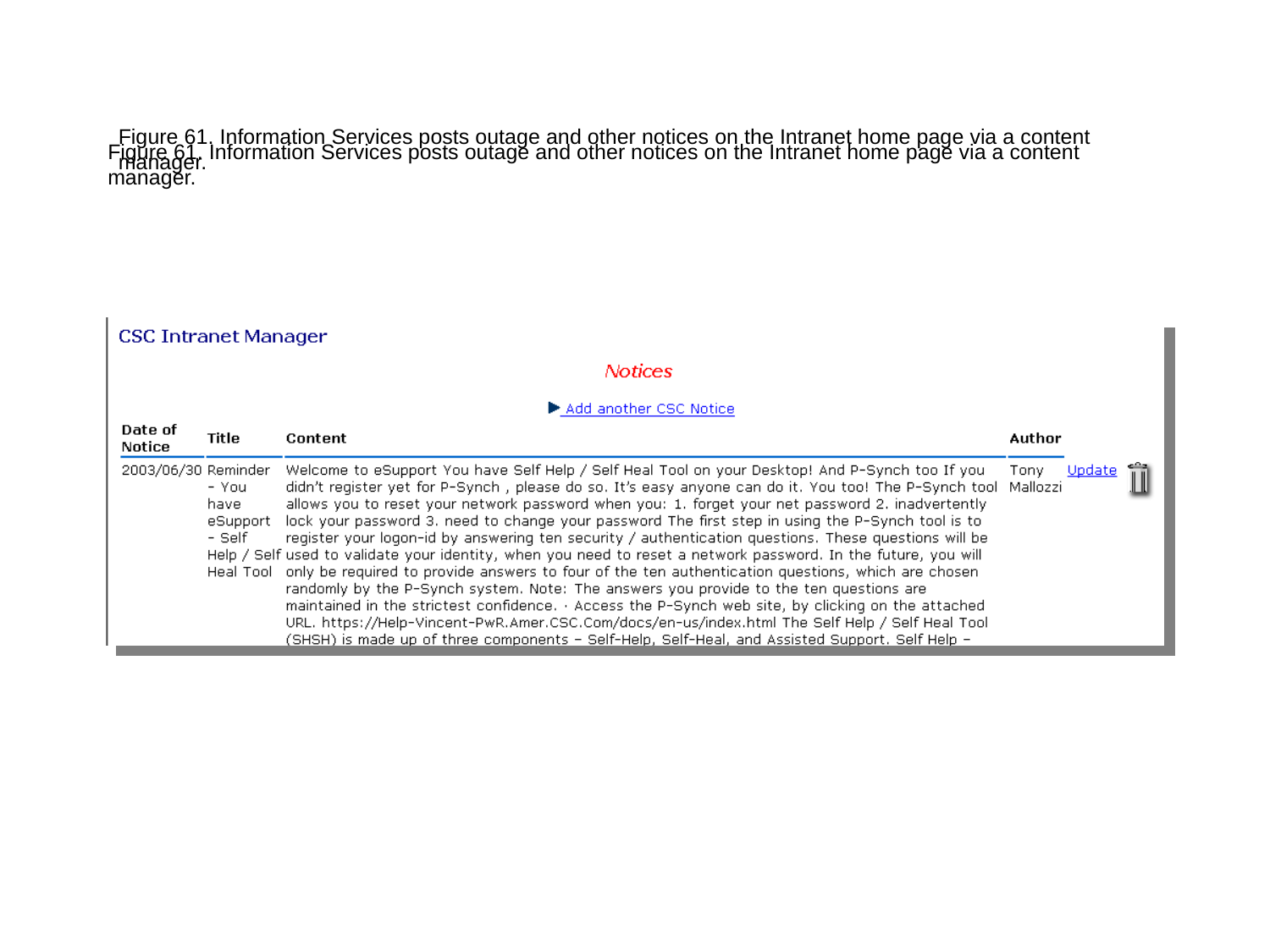

# Figure 61. Information Services posts outage and other notices on the Intranet home page via a content manager.
Figure 61. Information Services posts outage and other notices on the Intranet home page via a content manager.

## Slide 27
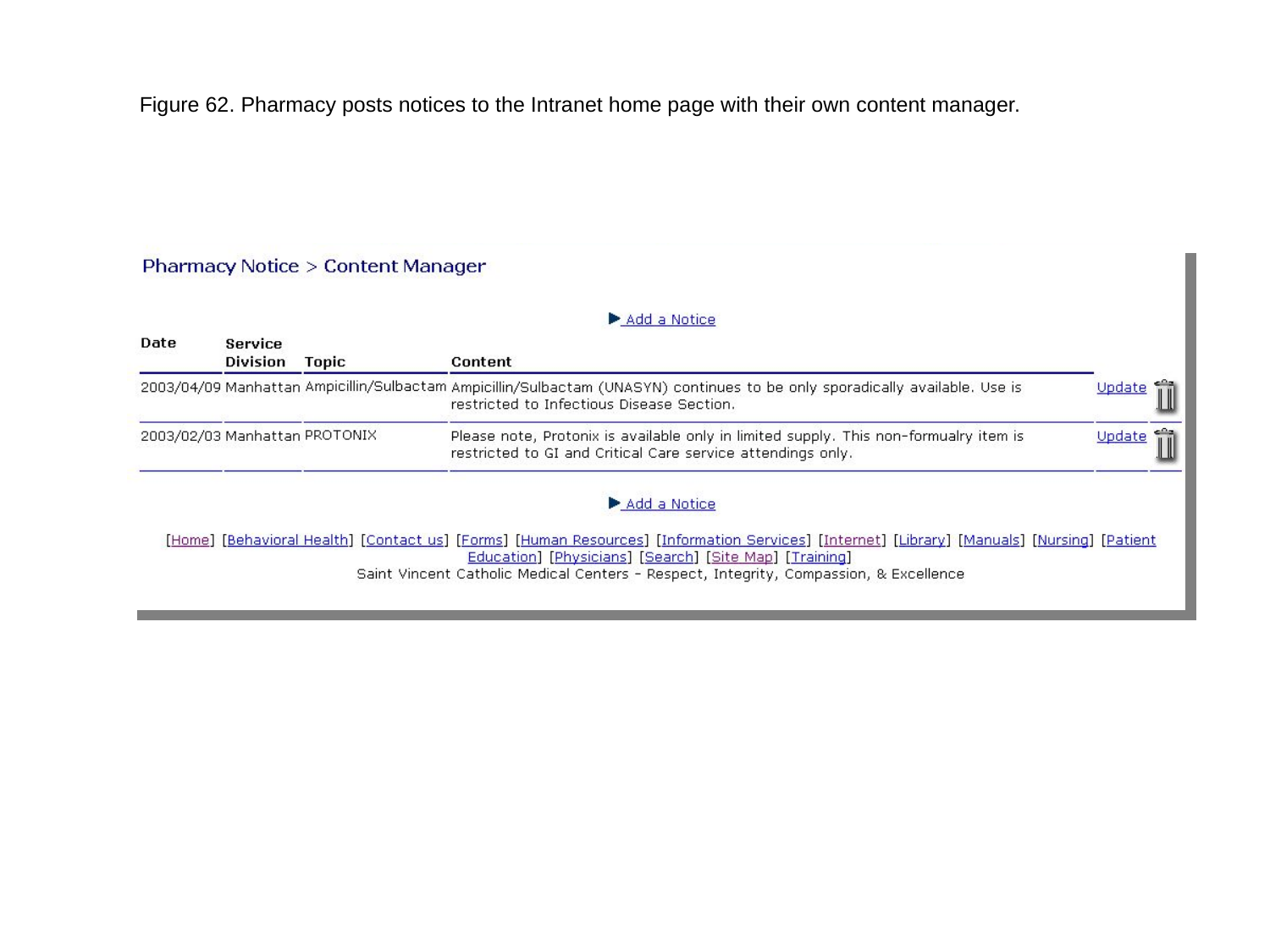

Figure 62. Pharmacy posts notices to the Intranet home page with their own content manager.

## Slide 28
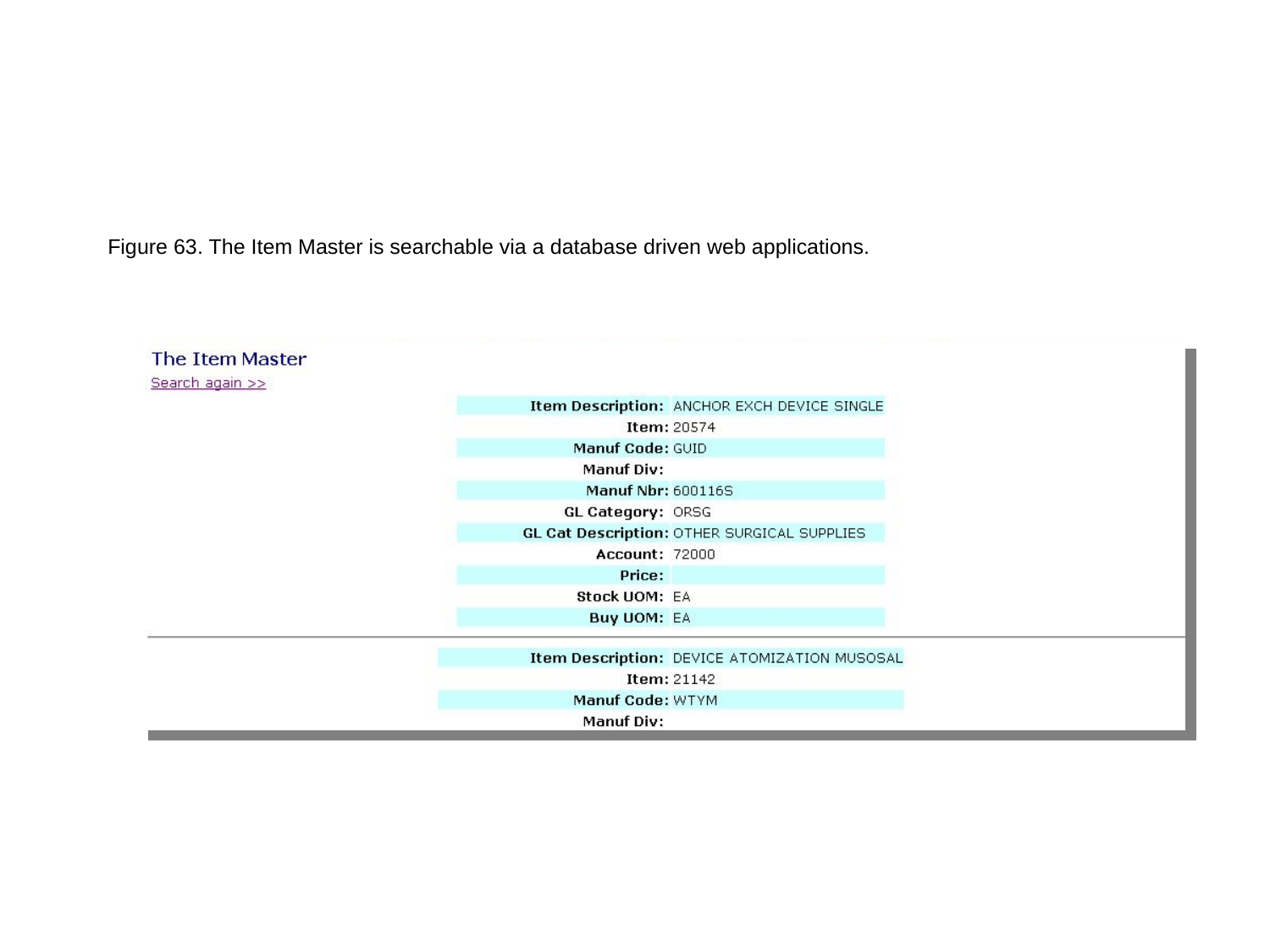

# Figure 63. The Item Master is searchable via a database driven web applications.

## Slide 29
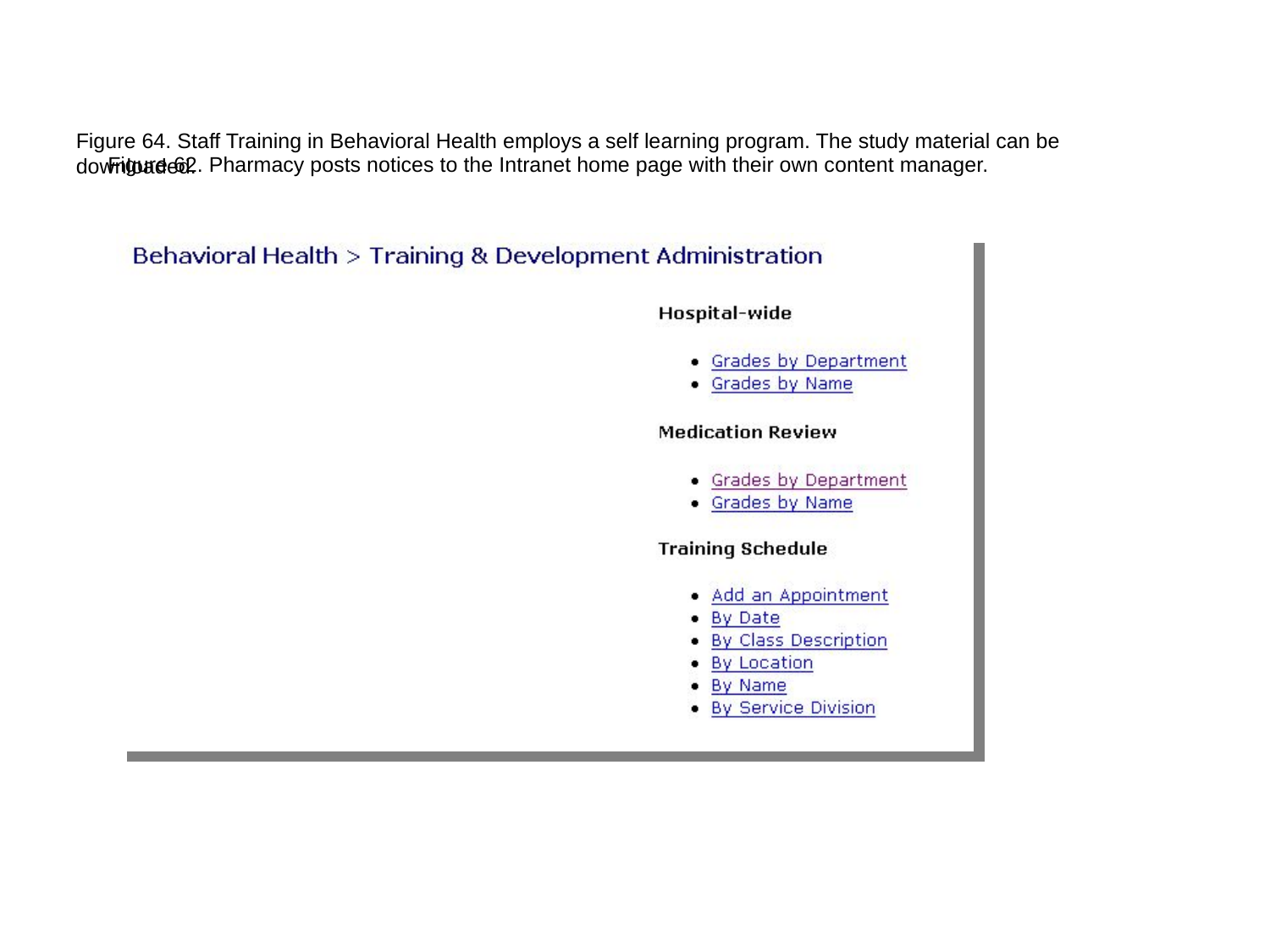

# Figure 62. Pharmacy posts notices to the Intranet home page with their own content manager.
Figure 64. Staff Training in Behavioral Health employs a self learning program. The study material can be downloaded.

## Slide 30
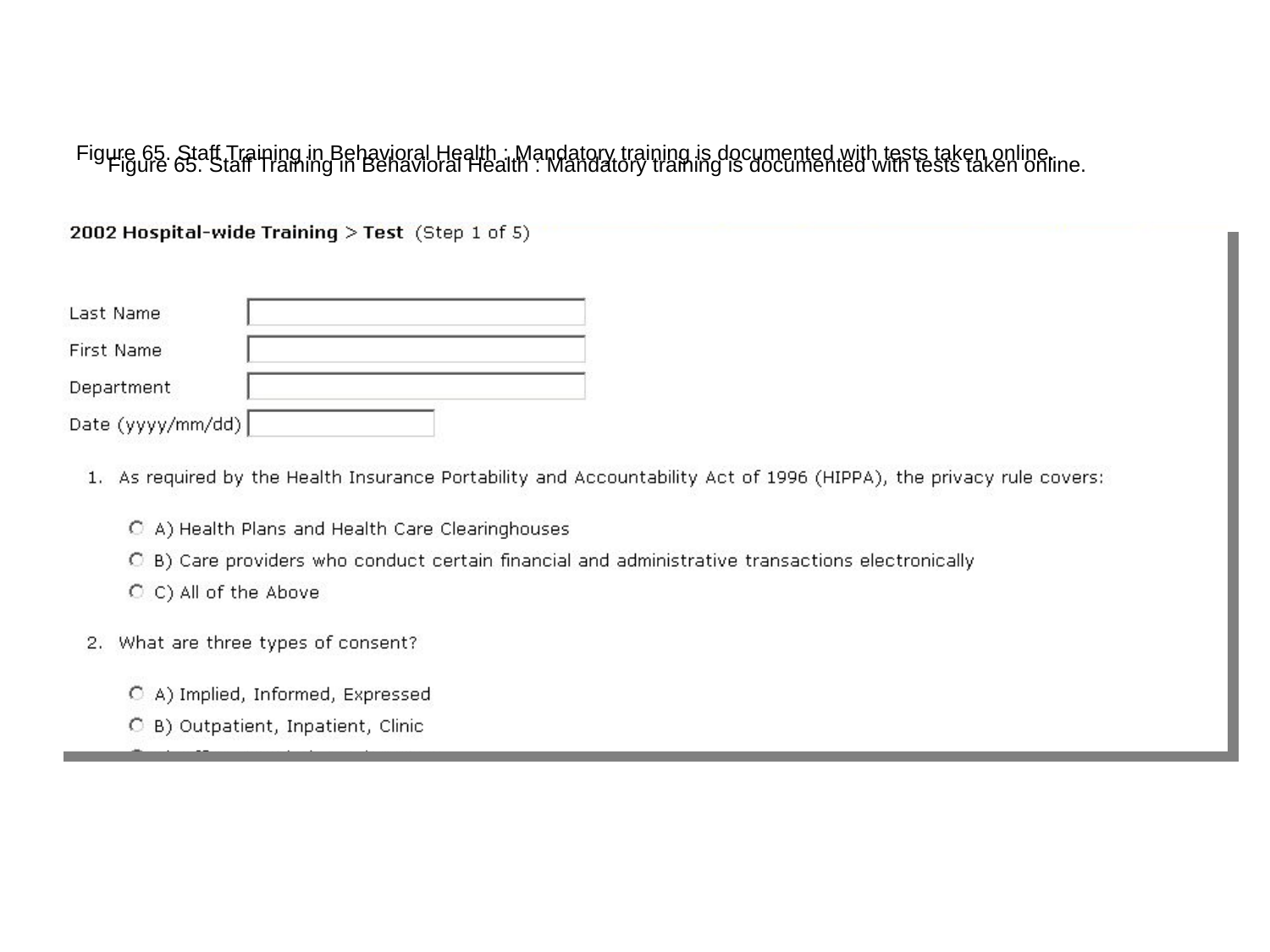

# Figure 65. Staff Training in Behavioral Health : Mandatory training is documented with tests taken online.
Figure 65. Staff Training in Behavioral Health : Mandatory training is documented with tests taken online.

## Slide 31
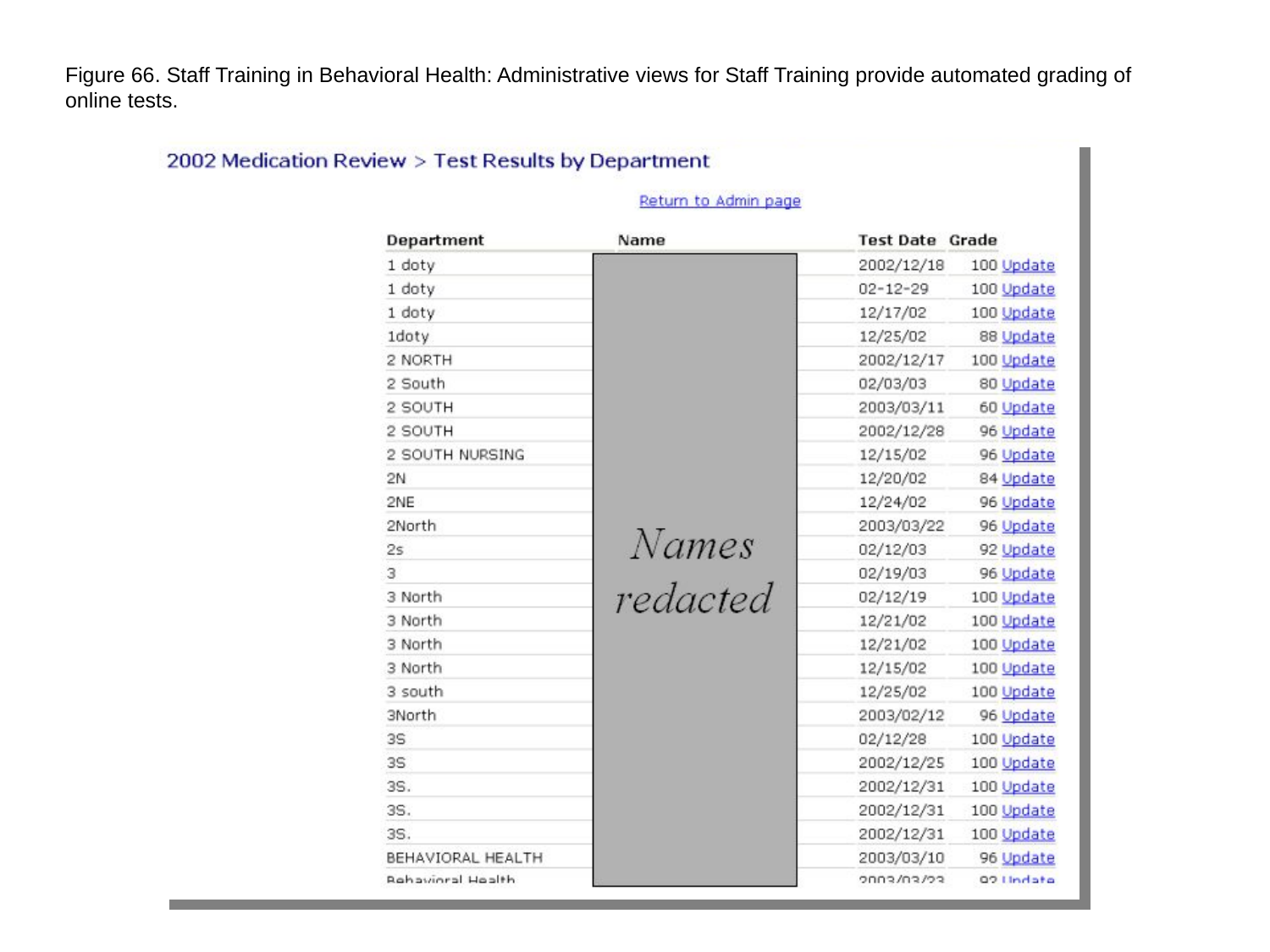

# Figure 66. Staff Training in Behavioral Health: Administrative views for Staff Training provide automated grading of online tests.

## Slide 32
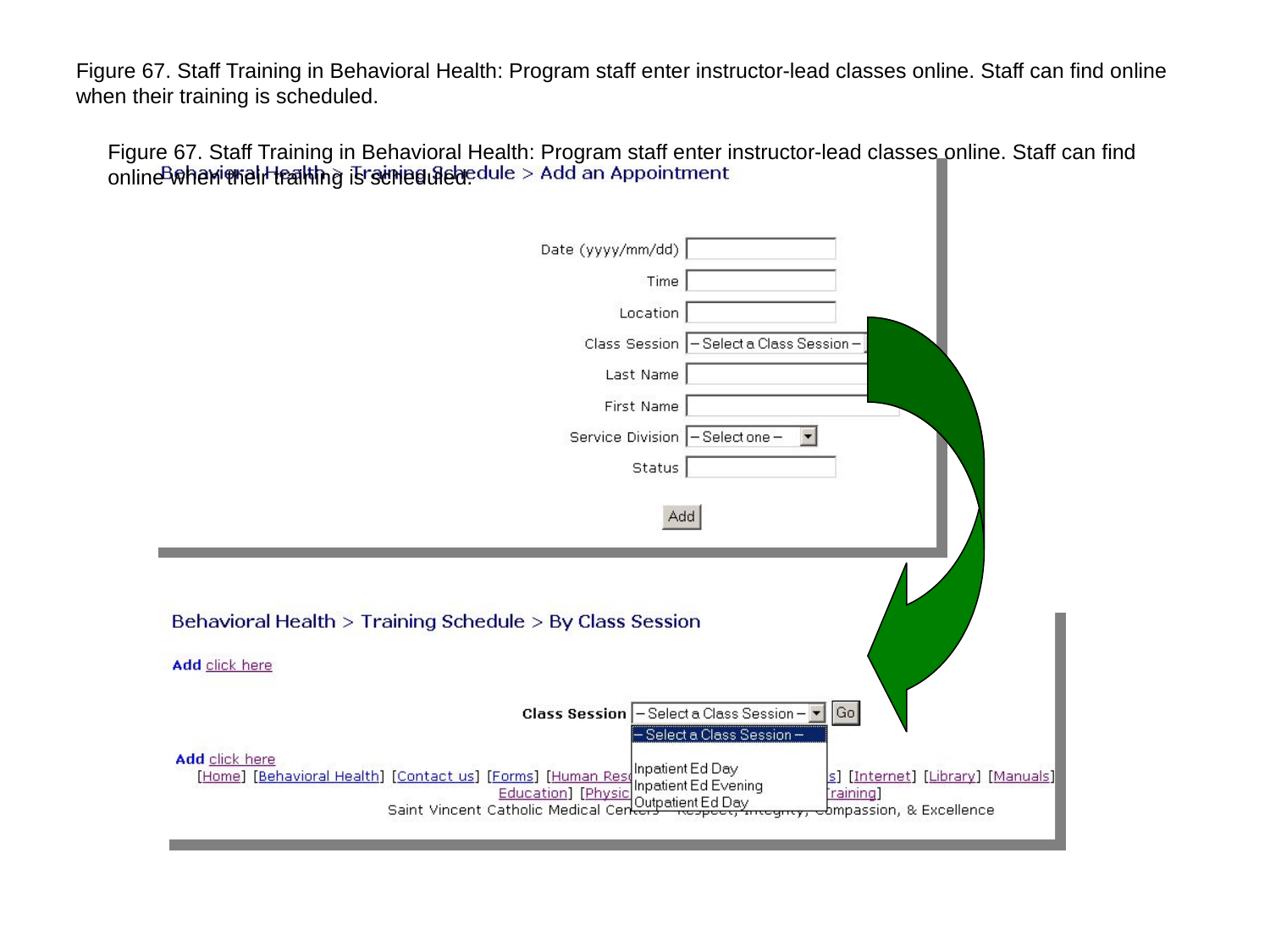

Figure 67. Staff Training in Behavioral Health: Program staff enter instructor-lead classes online. Staff can find online when their training is scheduled.
# Figure 67. Staff Training in Behavioral Health: Program staff enter instructor-lead classes online. Staff can find online when their training is scheduled.

## Slide 33
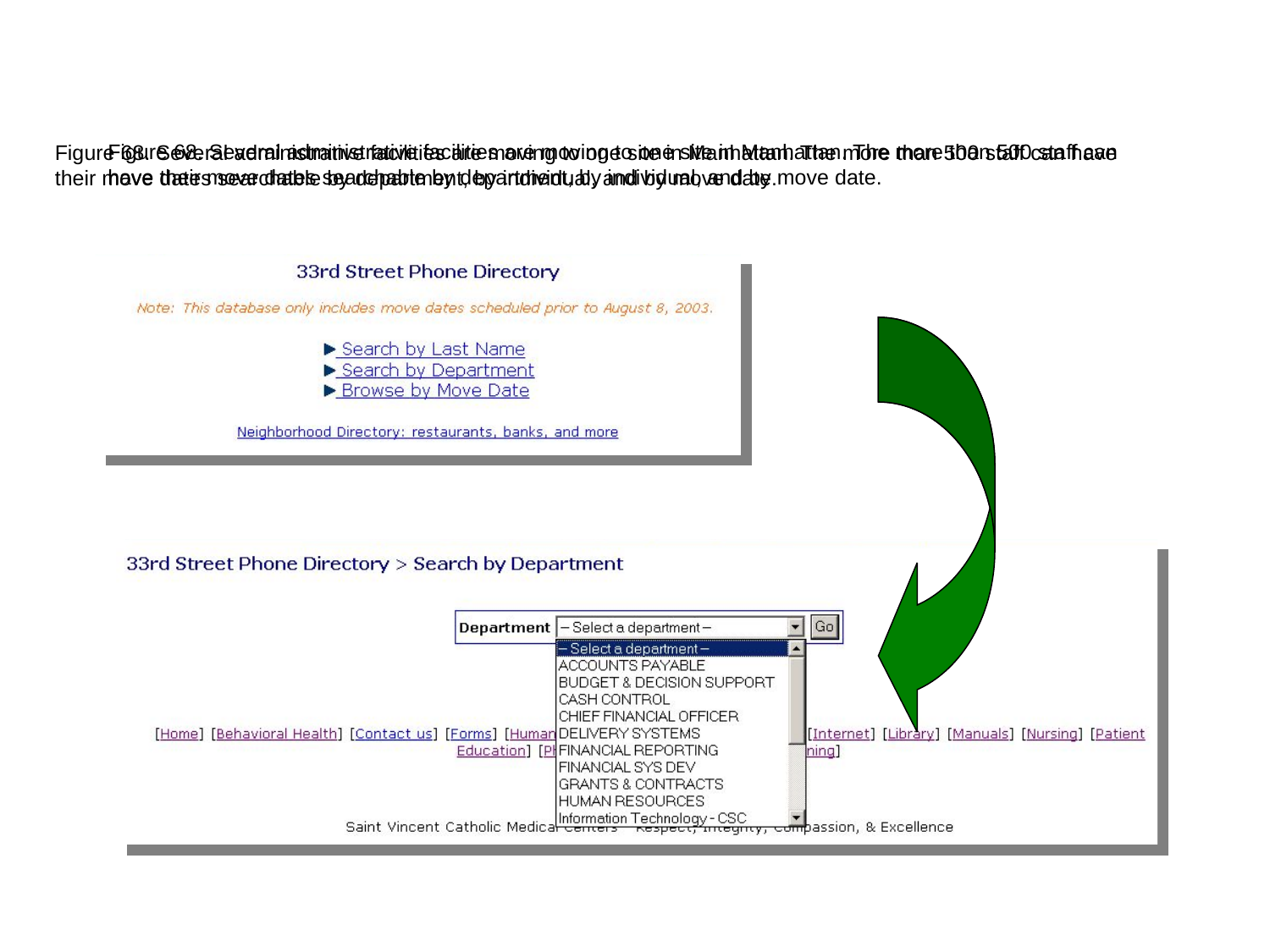

# Figure 68. Several administrative facilities are moving to one site in Manhattan. The more than 500 staff can have their move dates searchable by department, by individual, and by move date.
Figure 68. Several administrative facilities are moving to one site in Manhattan. The more than 500 staff can have their move dates searchable by department, by individual, and by move date.

## Slide 34
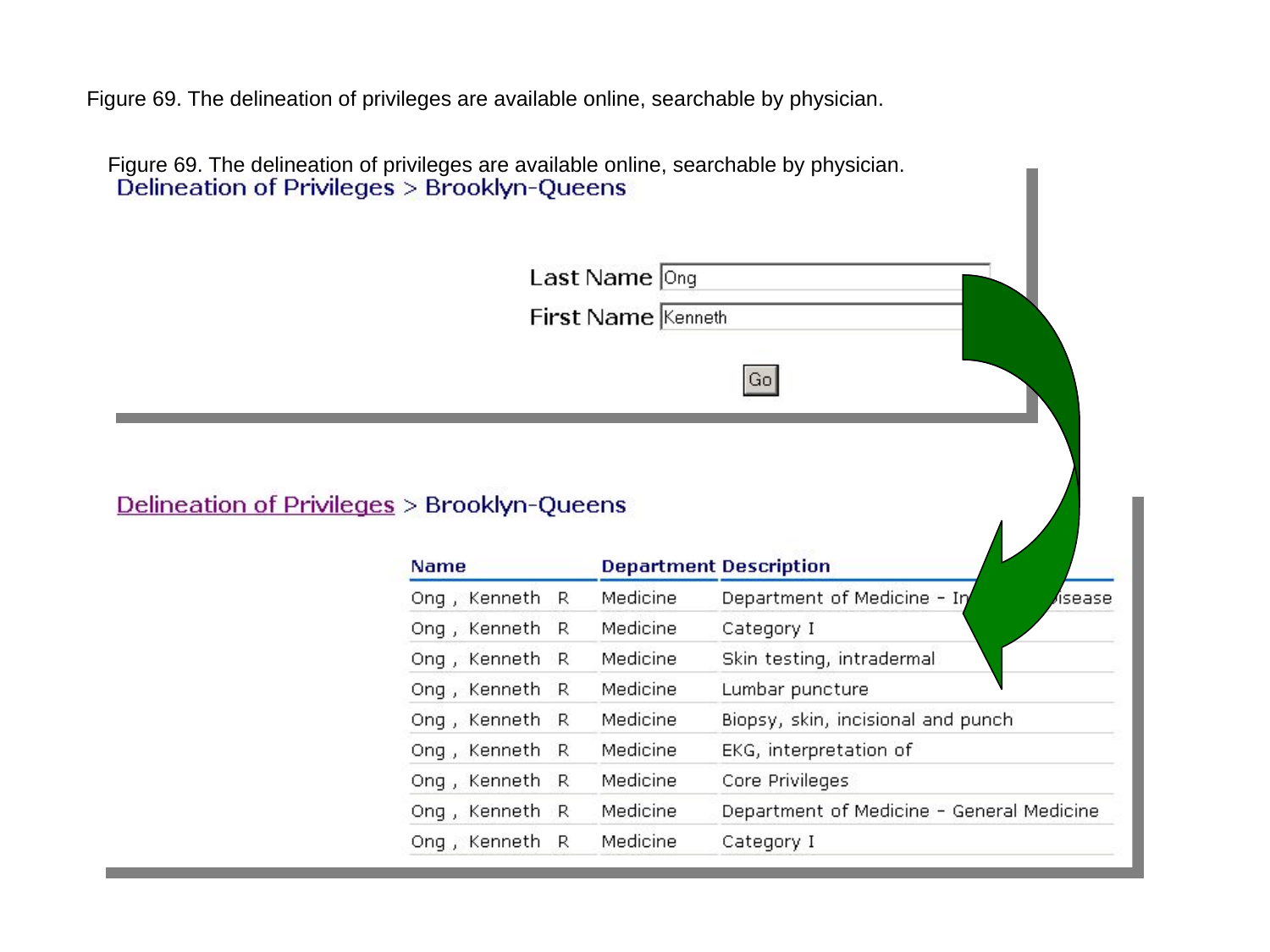

Figure 69. The delineation of privileges are available online, searchable by physician.
# Figure 69. The delineation of privileges are available online, searchable by physician.

## Slide 35
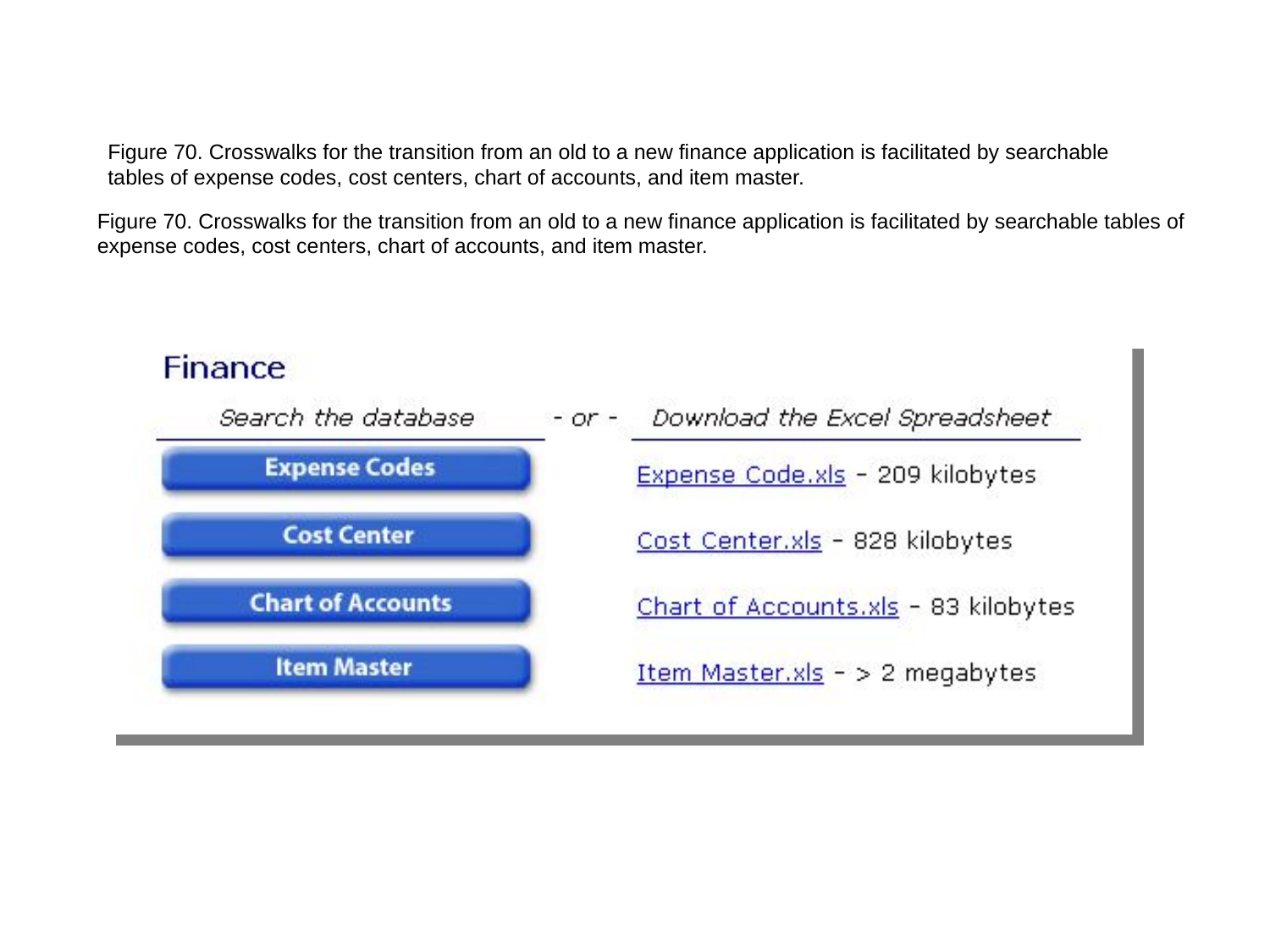

# Figure 70. Crosswalks for the transition from an old to a new finance application is facilitated by searchable tables of expense codes, cost centers, chart of accounts, and item master.
Figure 70. Crosswalks for the transition from an old to a new finance application is facilitated by searchable tables of expense codes, cost centers, chart of accounts, and item master.

## Slide 36
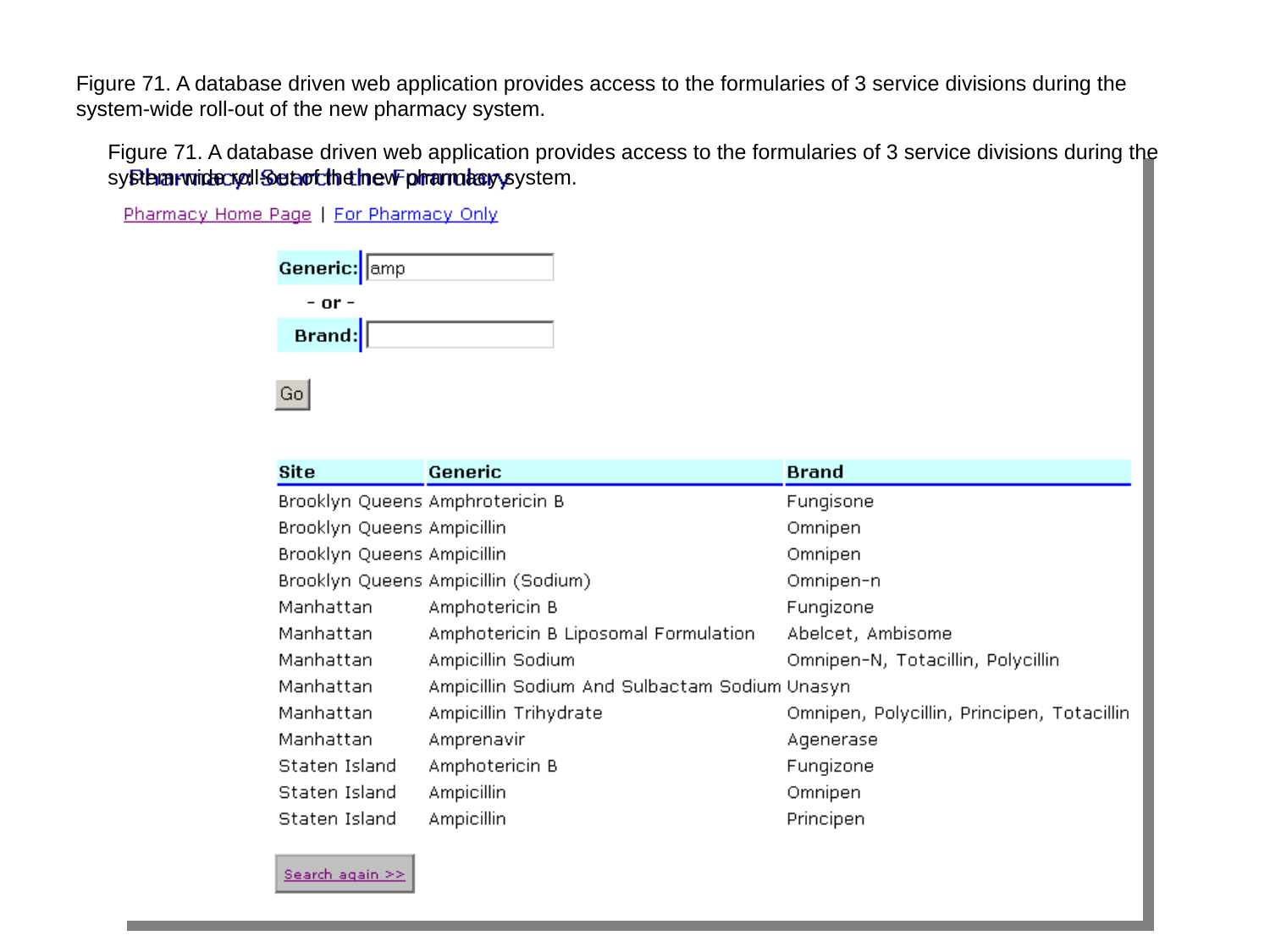

Figure 71. A database driven web application provides access to the formularies of 3 service divisions during the system-wide roll-out of the new pharmacy system.
# Figure 71. A database driven web application provides access to the formularies of 3 service divisions during the system-wide roll-out of the new pharmacy system.

## Slide 37
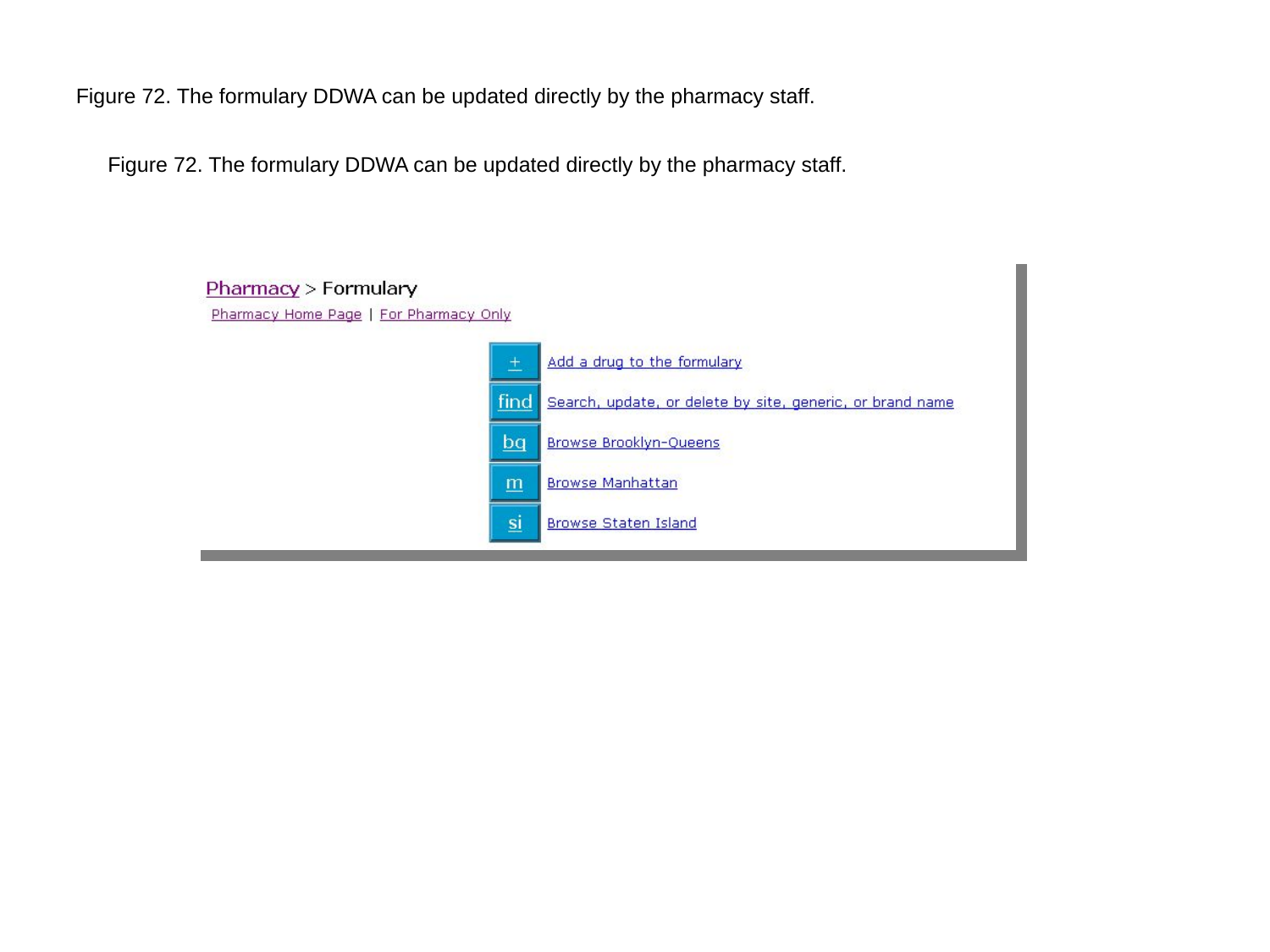

Figure 72. The formulary DDWA can be updated directly by the pharmacy staff.
# Figure 72. The formulary DDWA can be updated directly by the pharmacy staff.

## Slide 38
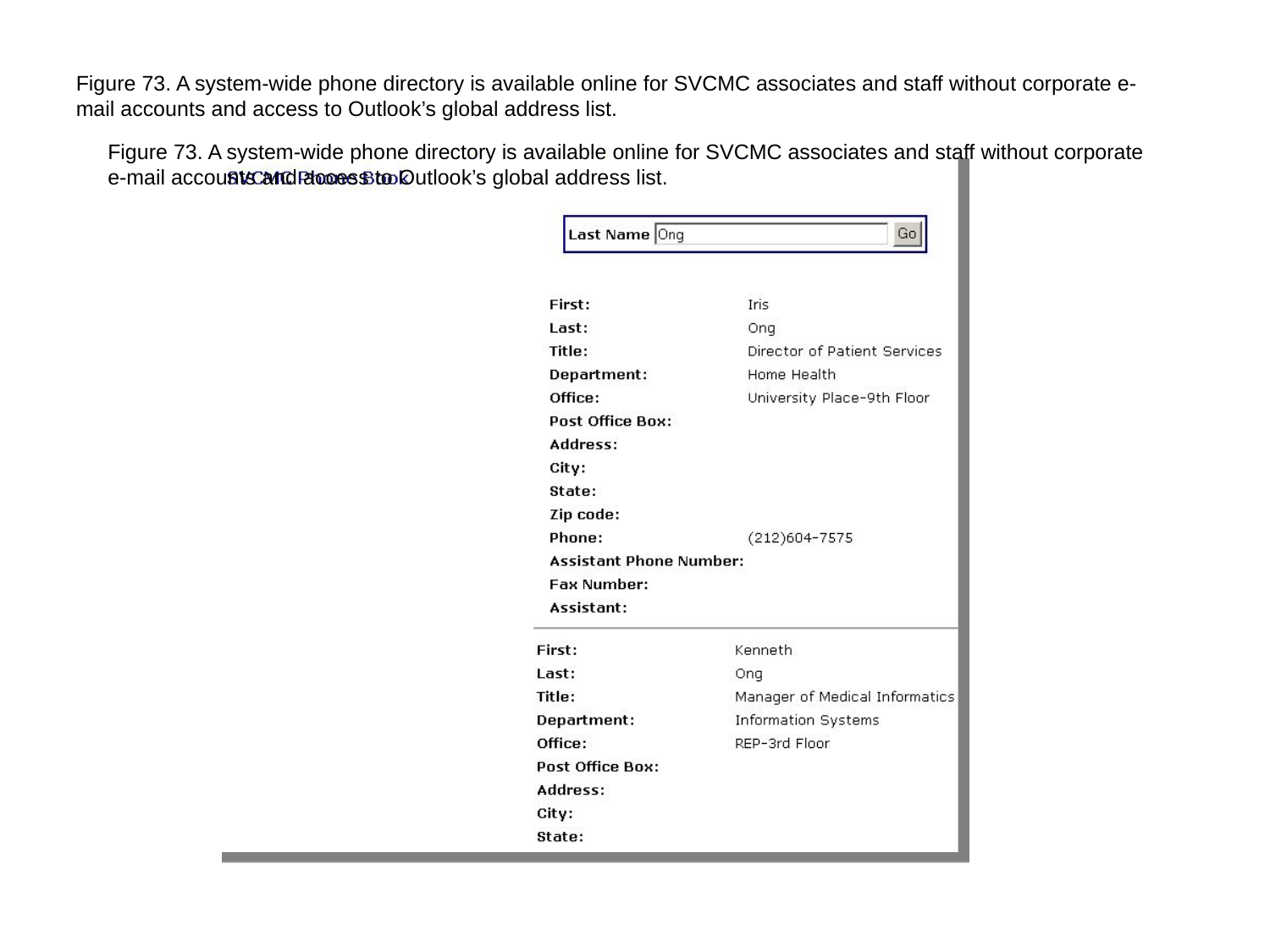

Figure 73. A system-wide phone directory is available online for SVCMC associates and staff without corporate e-mail accounts and access to Outlook’s global address list.
# Figure 73. A system-wide phone directory is available online for SVCMC associates and staff without corporate e-mail accounts and access to Outlook’s global address list.

## Slide 39
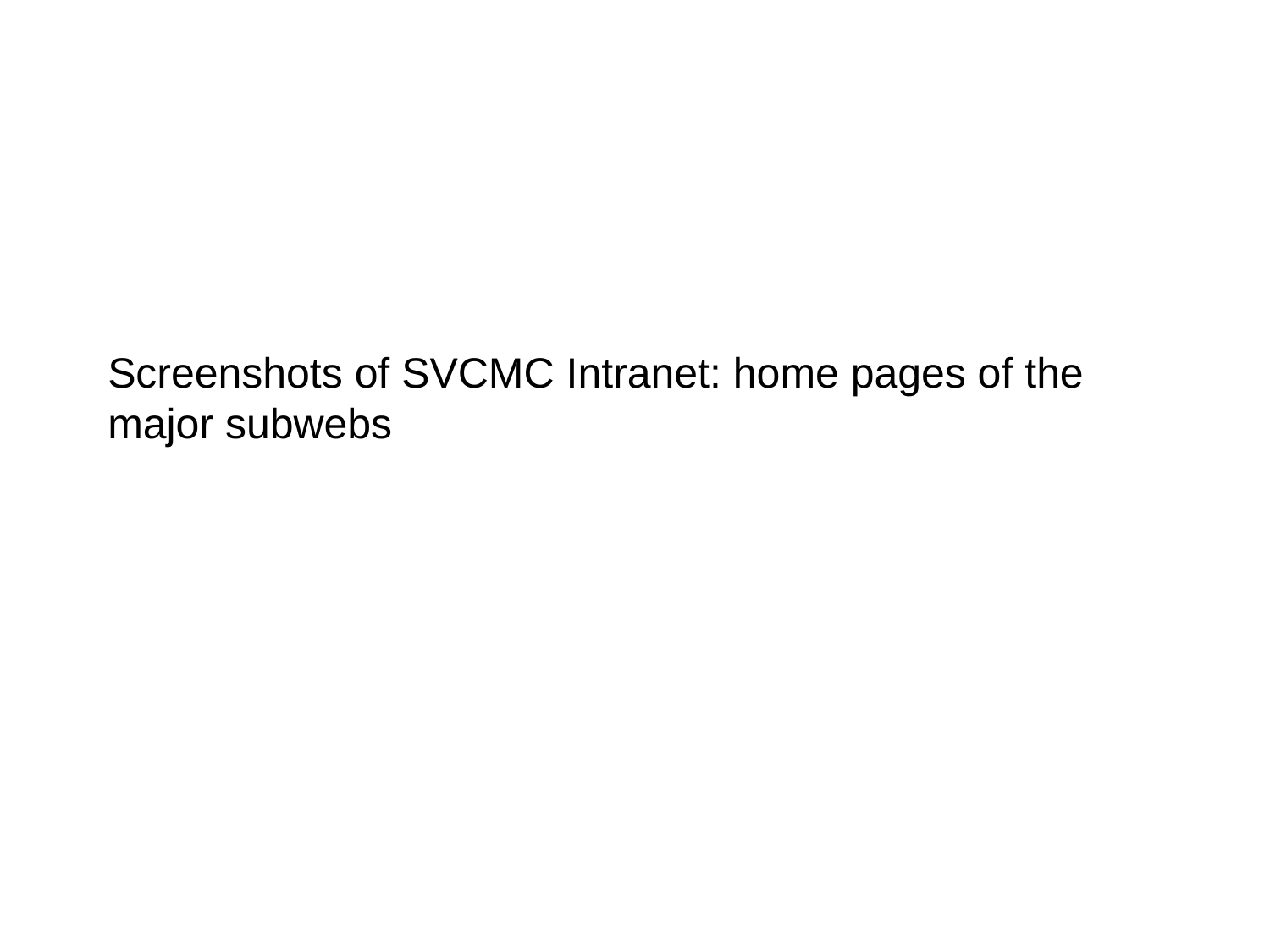

# Screenshots of SVCMC Intranet: home pages of the major subwebs

## Slide 40
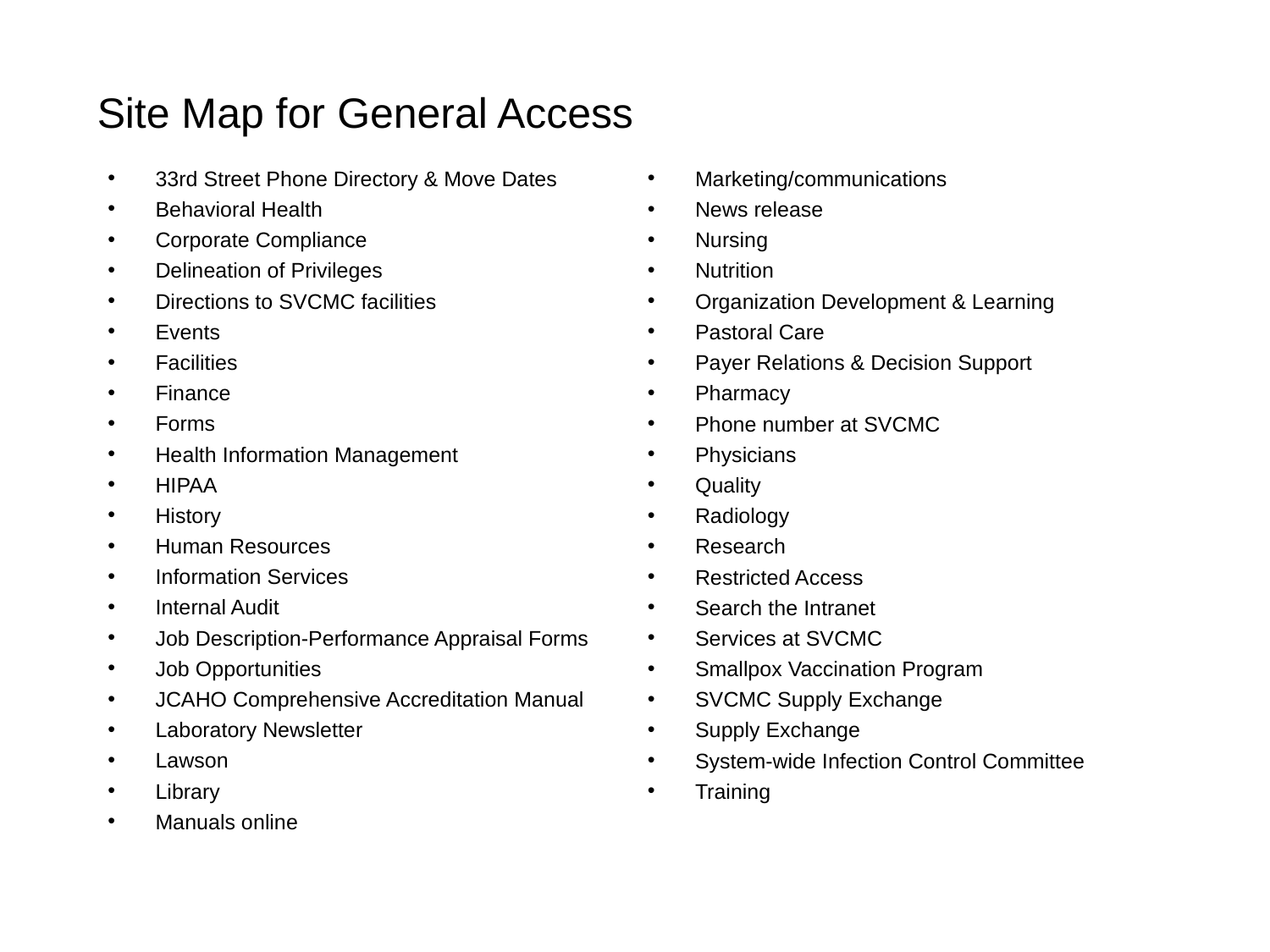

# Site Map for General Access
33rd Street Phone Directory & Move Dates
Behavioral Health
Corporate Compliance
Delineation of Privileges
Directions to SVCMC facilities
Events
Facilities
Finance
Forms
Health Information Management
HIPAA
History
Human Resources
Information Services
Internal Audit
Job Description-Performance Appraisal Forms
Job Opportunities
JCAHO Comprehensive Accreditation Manual
Laboratory Newsletter
Lawson
Library
Manuals online
Marketing/communications
News release
Nursing
Nutrition
Organization Development & Learning
Pastoral Care
Payer Relations & Decision Support
Pharmacy
Phone number at SVCMC
Physicians
Quality
Radiology
Research
Restricted Access
Search the Intranet
Services at SVCMC
Smallpox Vaccination Program
SVCMC Supply Exchange
Supply Exchange
System-wide Infection Control Committee
Training

## Slide 41
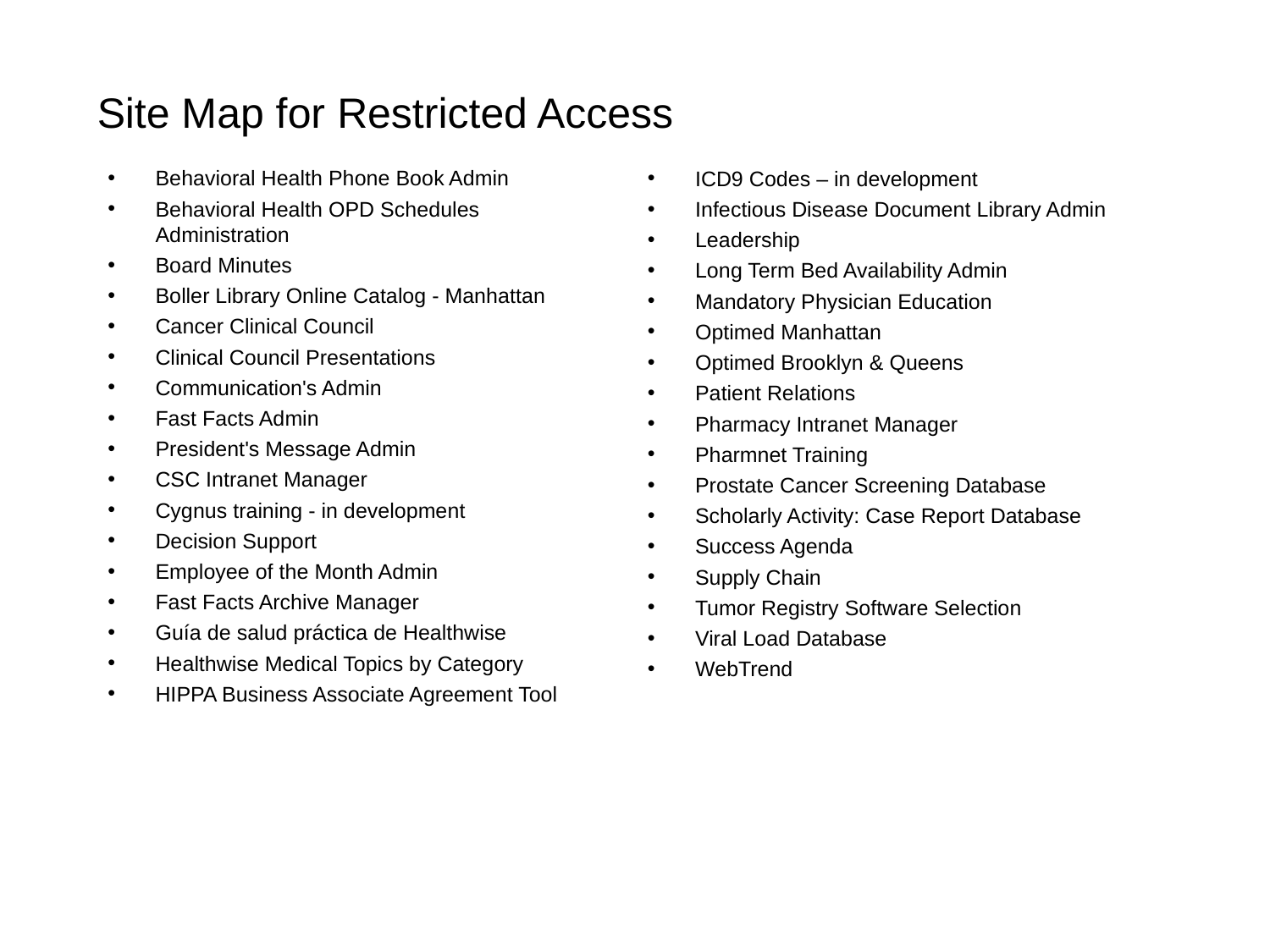

# Site Map for Restricted Access
Behavioral Health Phone Book Admin
Behavioral Health OPD Schedules Administration
Board Minutes
Boller Library Online Catalog - Manhattan
Cancer Clinical Council
Clinical Council Presentations
Communication's Admin
Fast Facts Admin
President's Message Admin
CSC Intranet Manager
Cygnus training - in development
Decision Support
Employee of the Month Admin
Fast Facts Archive Manager
Guía de salud práctica de Healthwise
Healthwise Medical Topics by Category
HIPPA Business Associate Agreement Tool
ICD9 Codes – in development
Infectious Disease Document Library Admin
Leadership
Long Term Bed Availability Admin
Mandatory Physician Education
Optimed Manhattan
Optimed Brooklyn & Queens
Patient Relations
Pharmacy Intranet Manager
Pharmnet Training
Prostate Cancer Screening Database
Scholarly Activity: Case Report Database
Success Agenda
Supply Chain
Tumor Registry Software Selection
Viral Load Database
WebTrend

## Slide 42
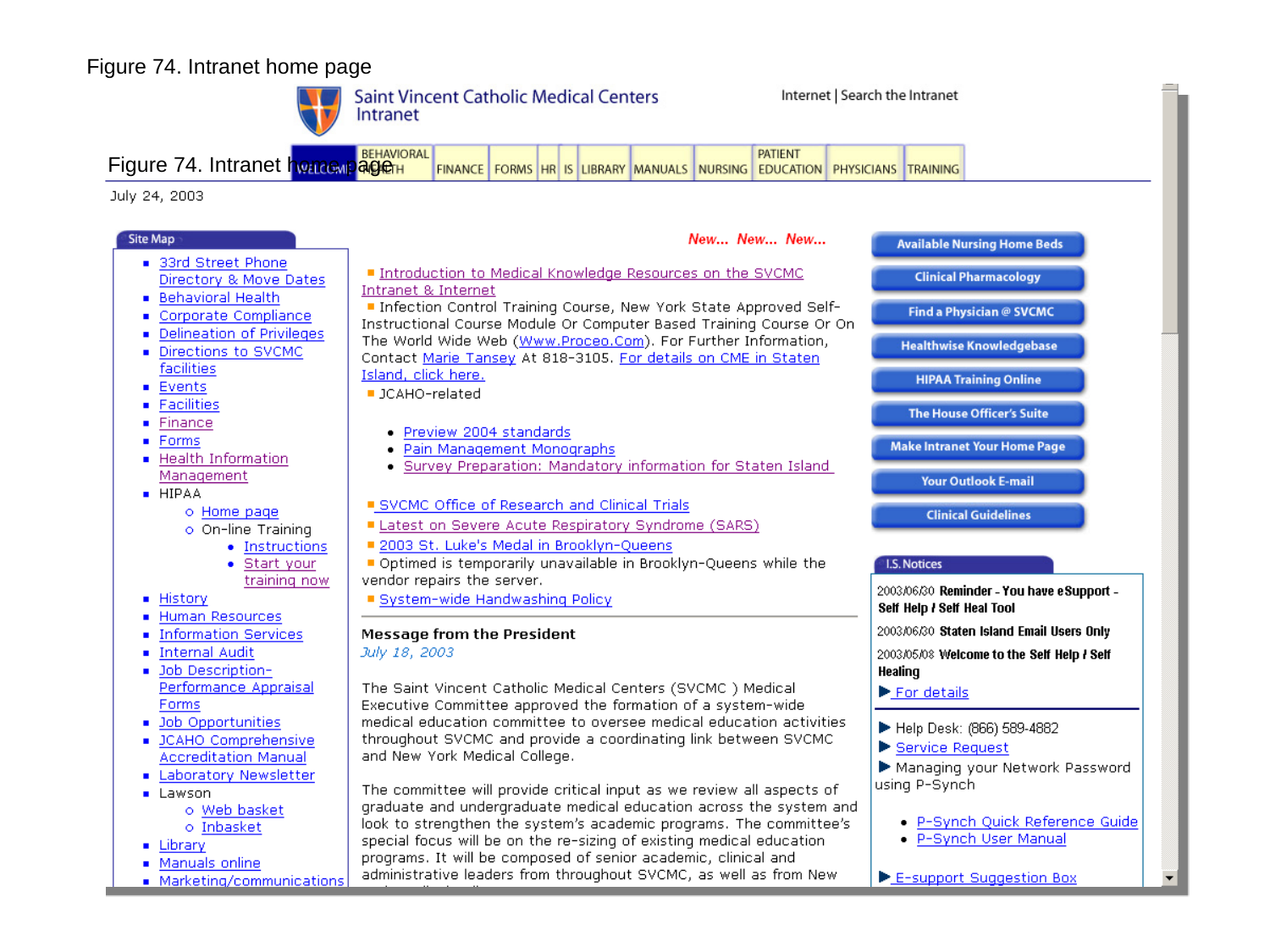

Figure 74. Intranet home page
# Figure 74. Intranet home page

## Slide 43
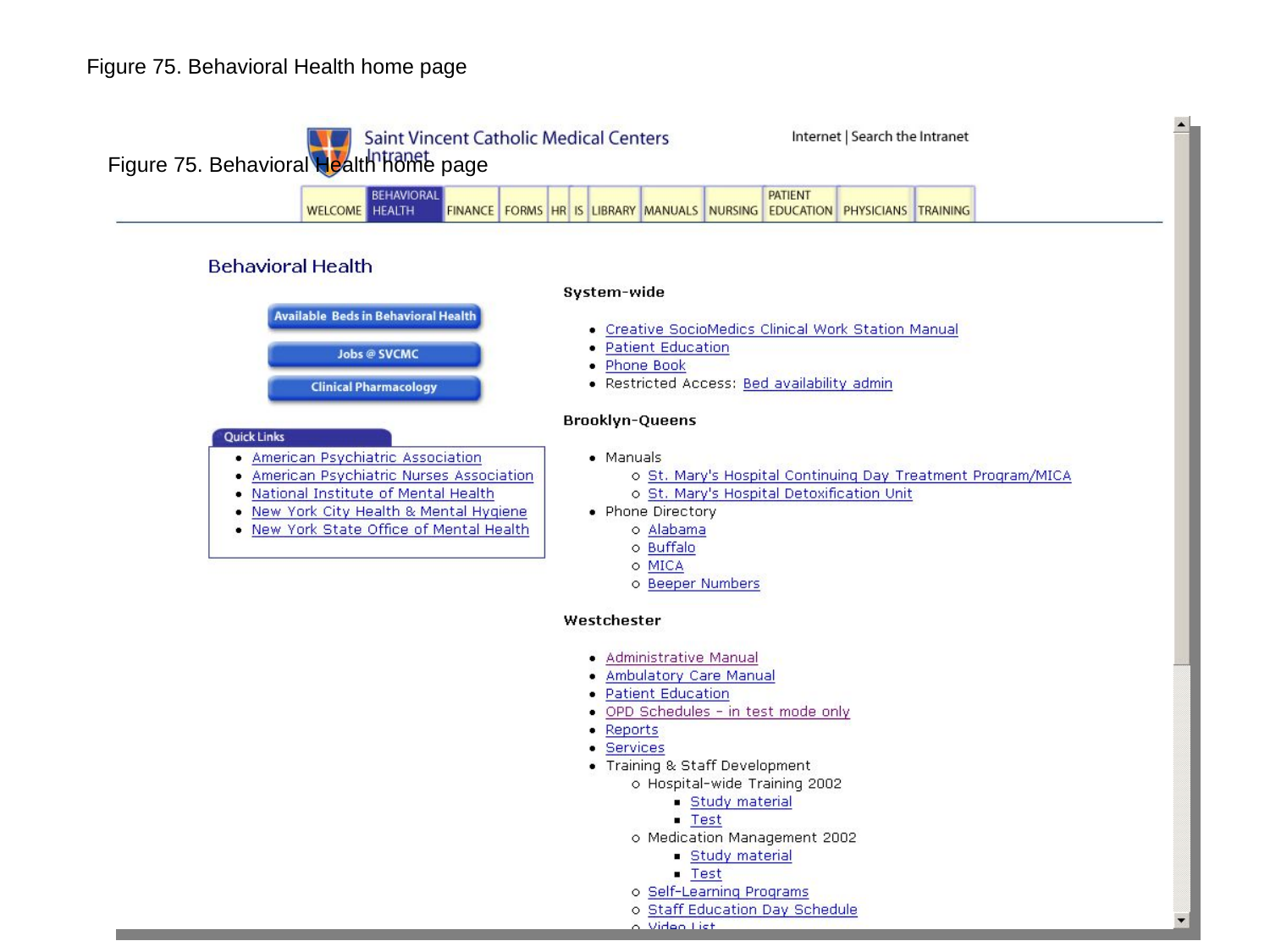

Figure 75. Behavioral Health home page
# Figure 75. Behavioral Health home page

## Slide 44
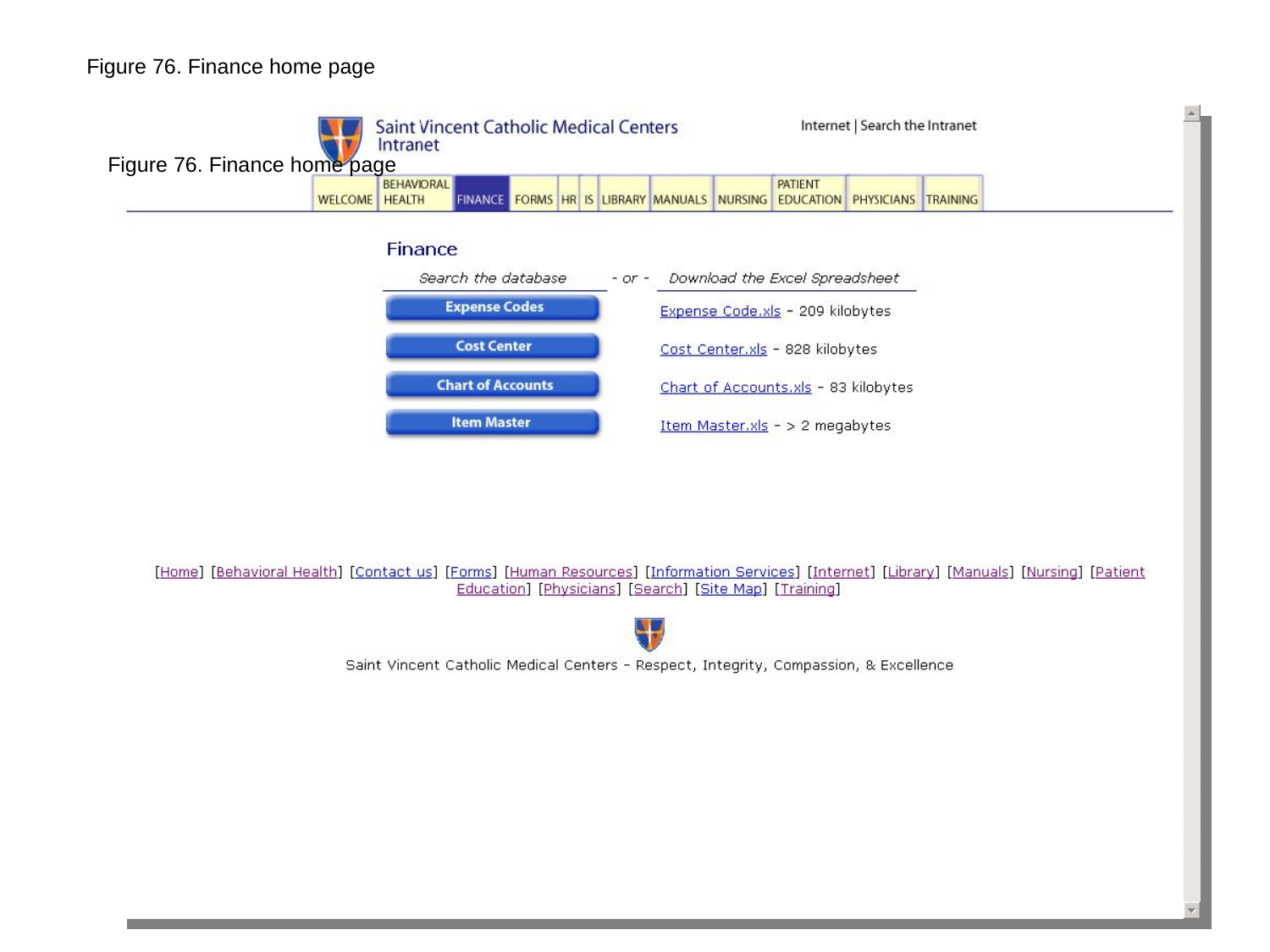

Figure 76. Finance home page
# Figure 76. Finance home page

## Slide 45
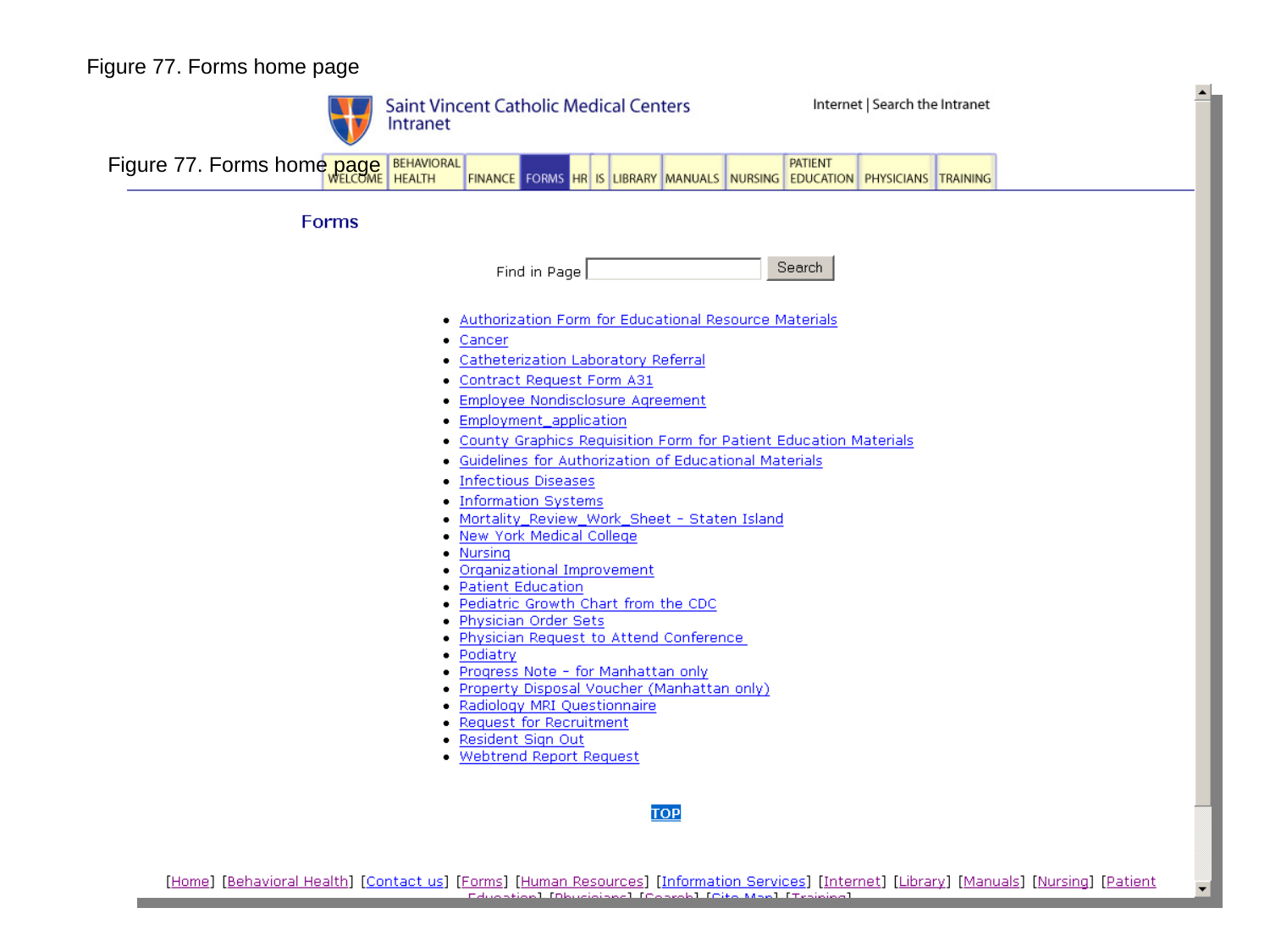

Figure 77. Forms home page
# Figure 77. Forms home page

## Slide 46
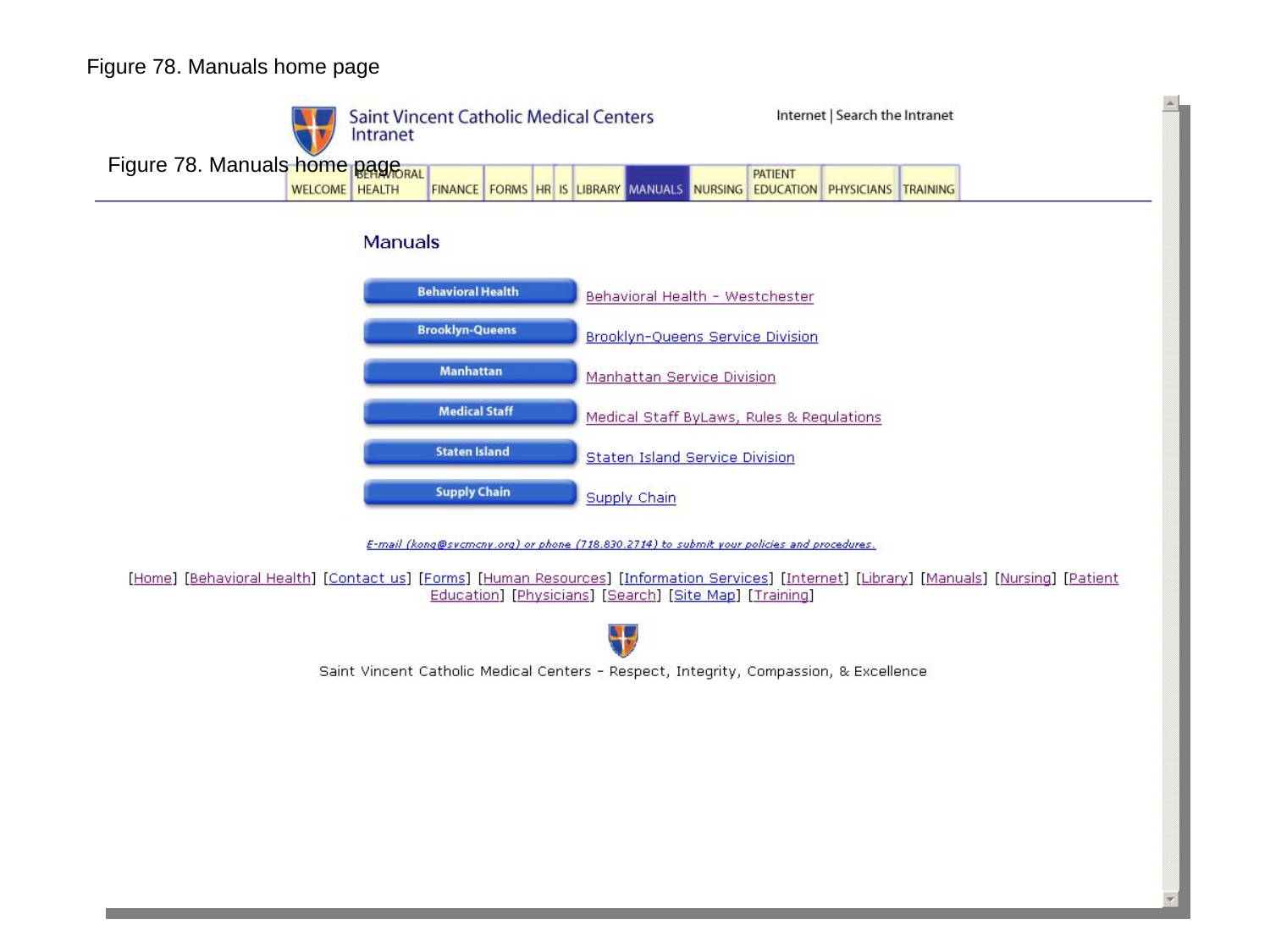

Figure 78. Manuals home page
# Figure 78. Manuals home page

## Slide 47
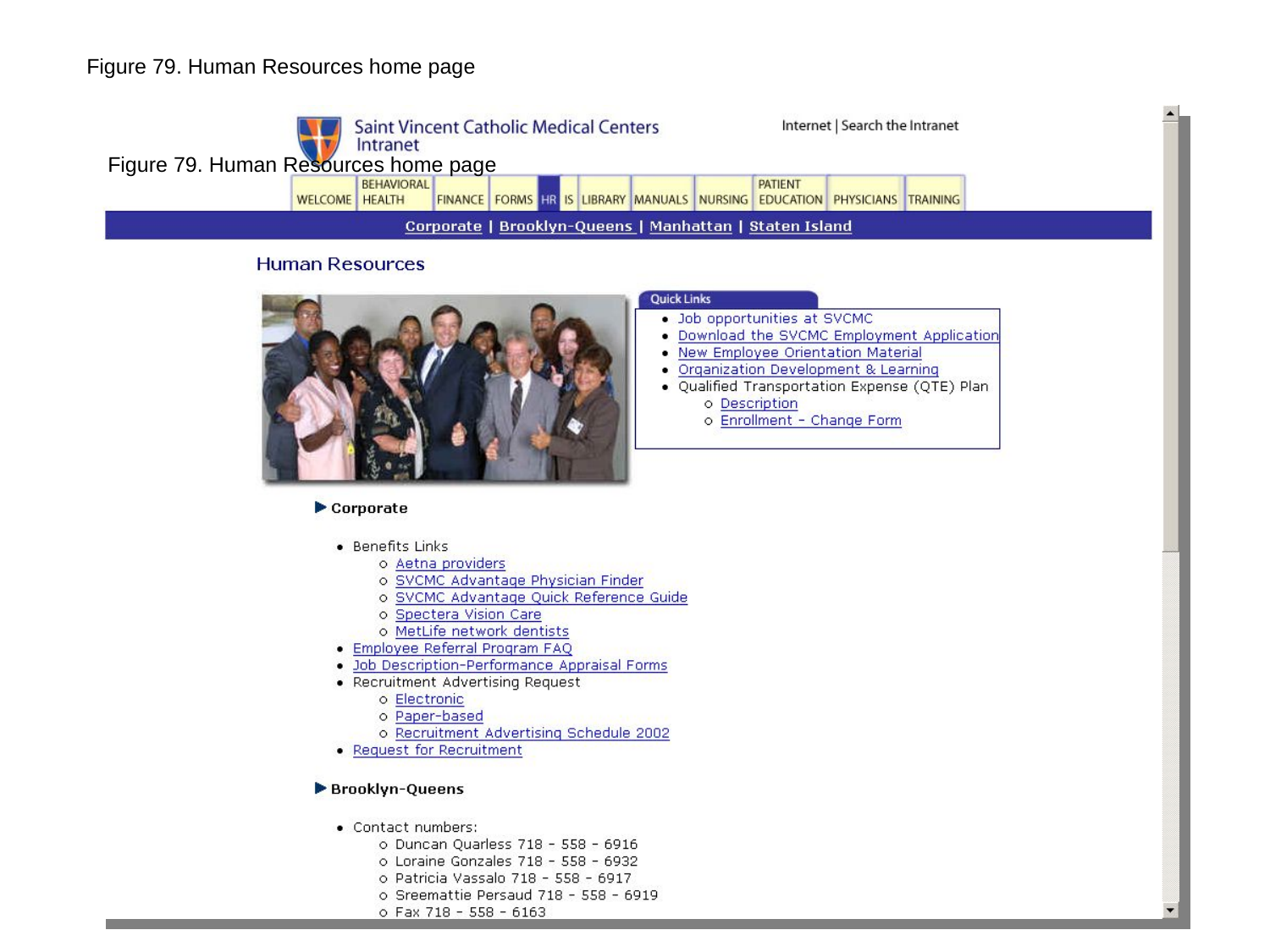

Figure 79. Human Resources home page
# Figure 79. Human Resources home page

## Slide 48
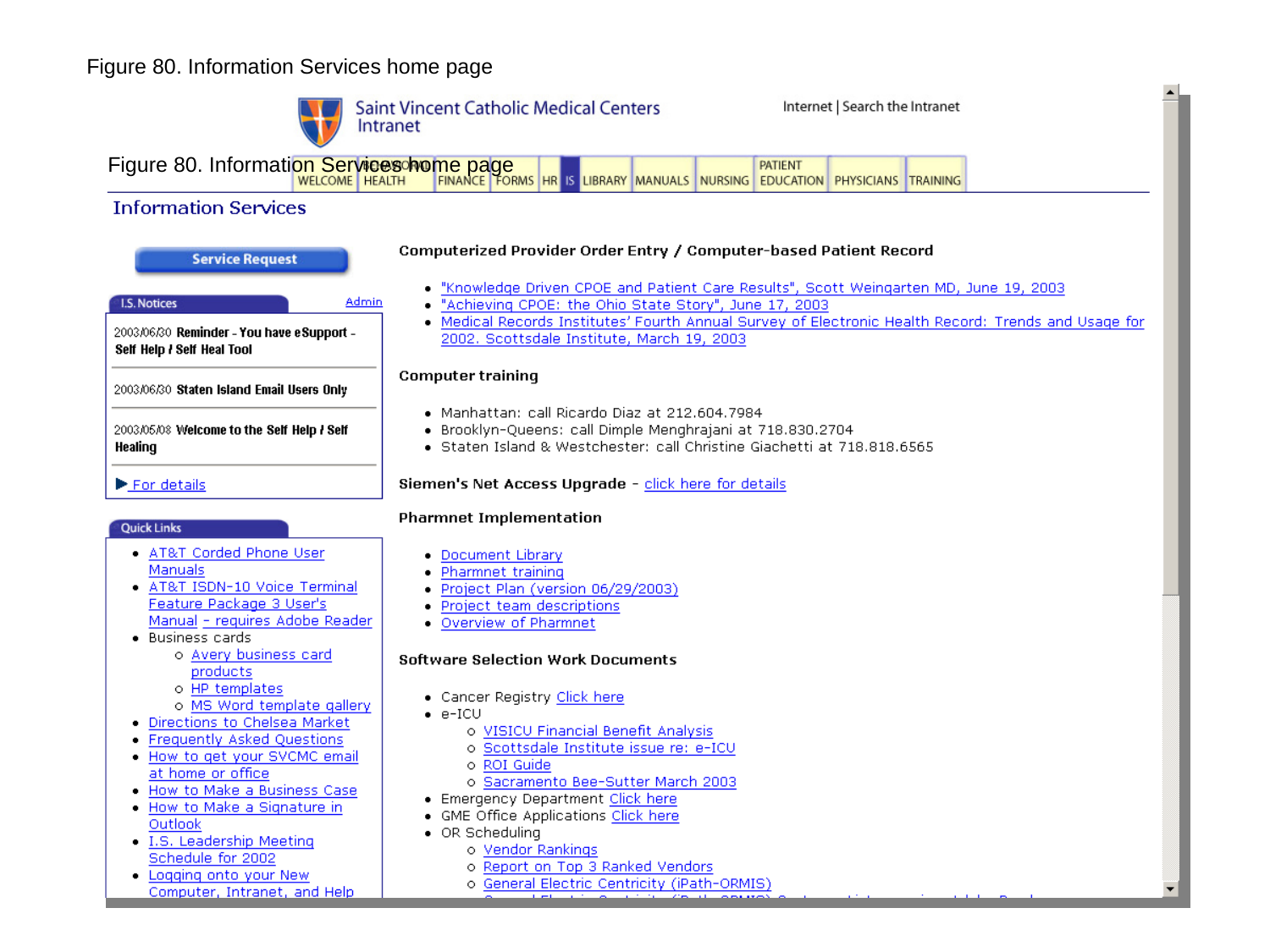

Figure 80. Information Services home page
# Figure 80. Information Services home page

## Slide 49
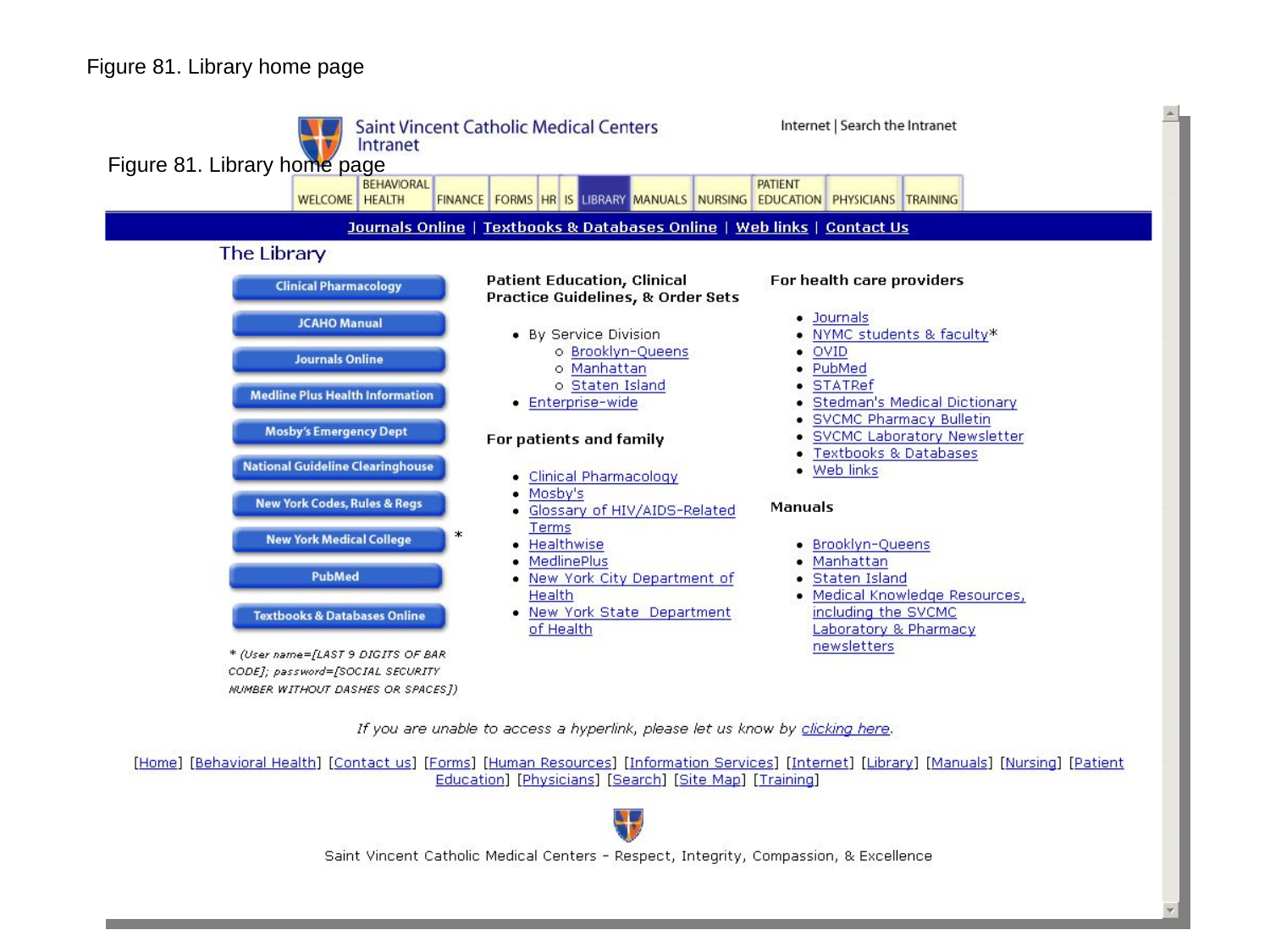

Figure 81. Library home page
# Figure 81. Library home page

## Slide 50
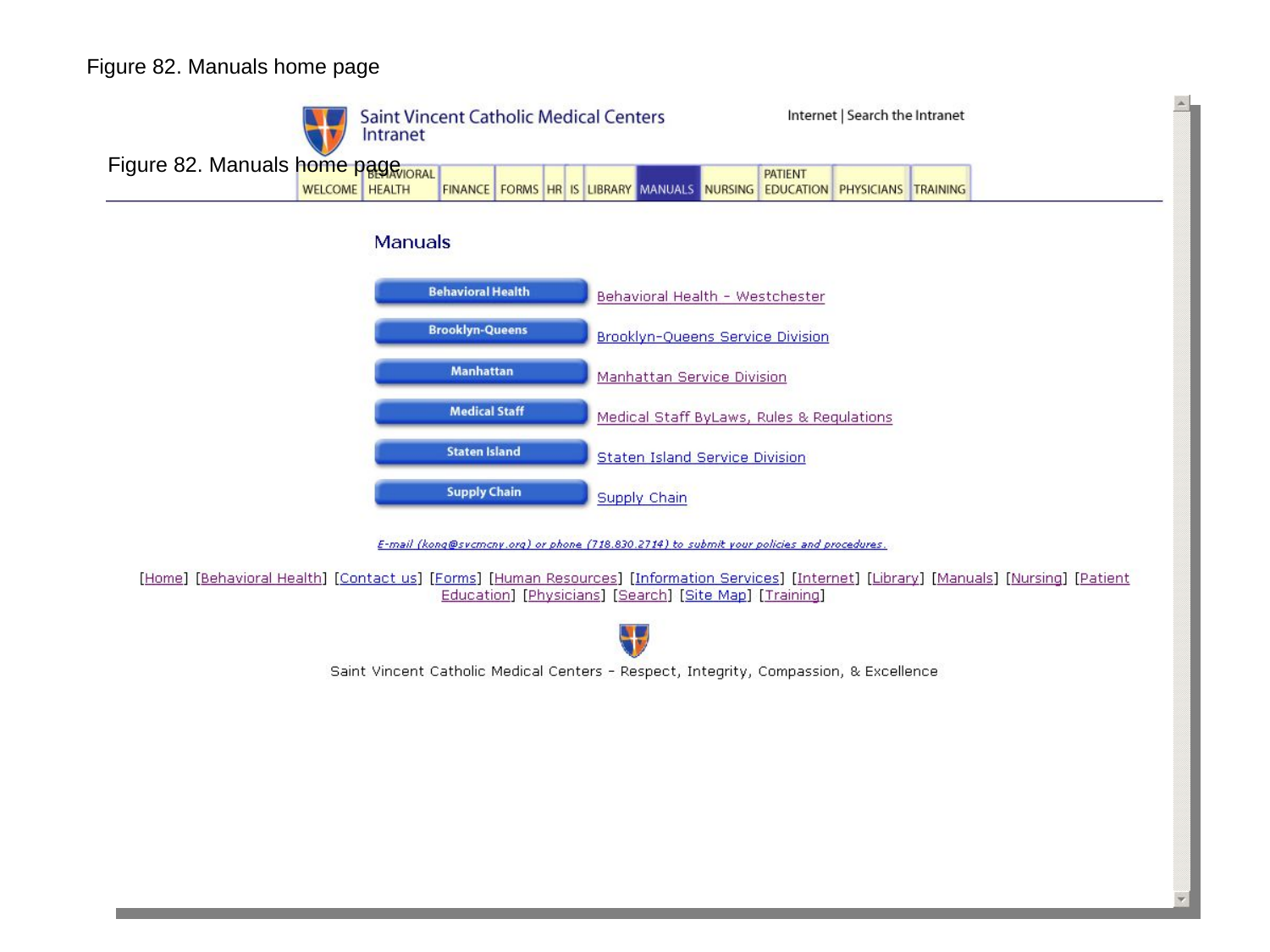

Figure 82. Manuals home page
# Figure 82. Manuals home page

## Slide 51
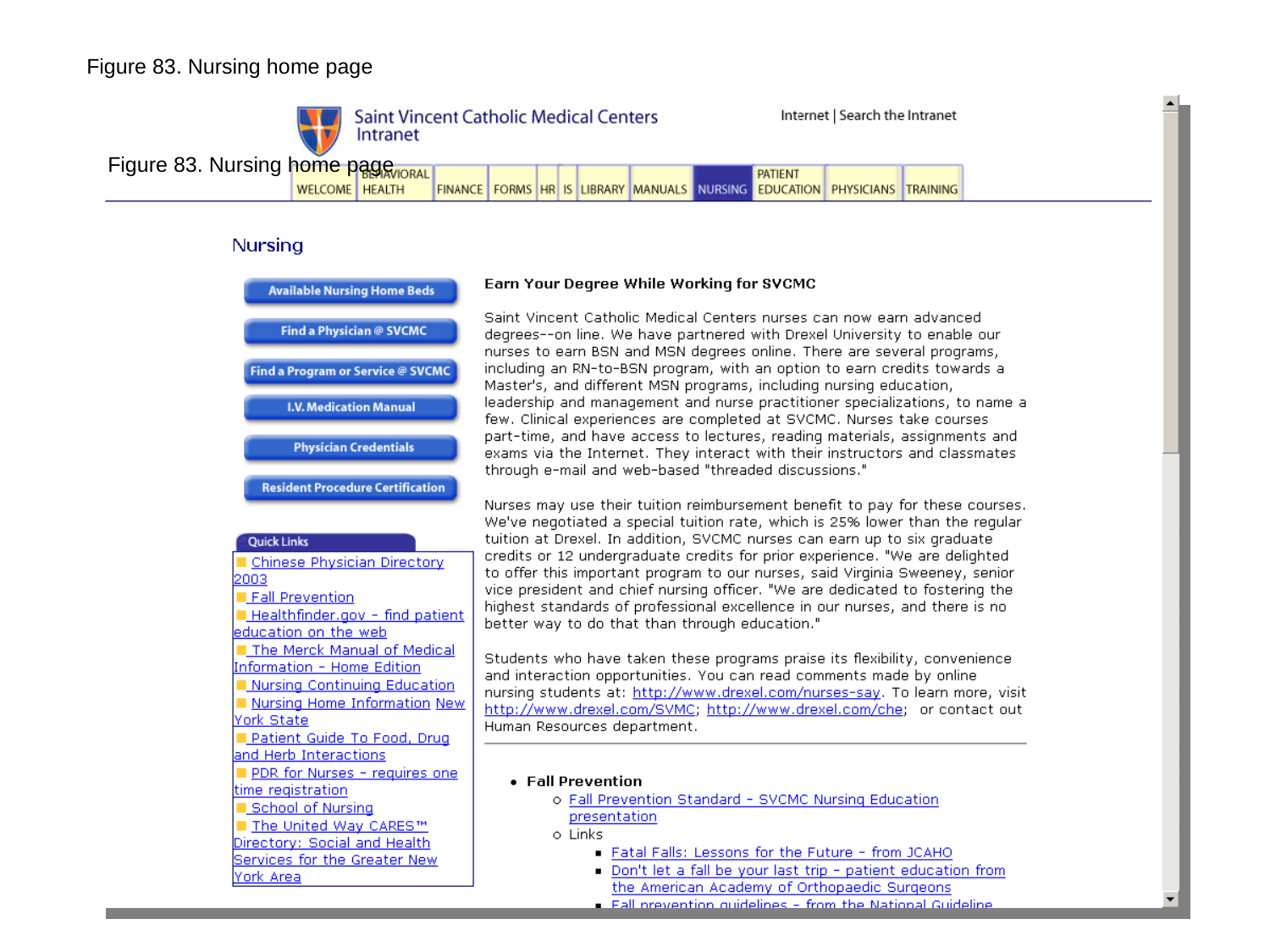

Figure 83. Nursing home page
# Figure 83. Nursing home page

## Slide 52
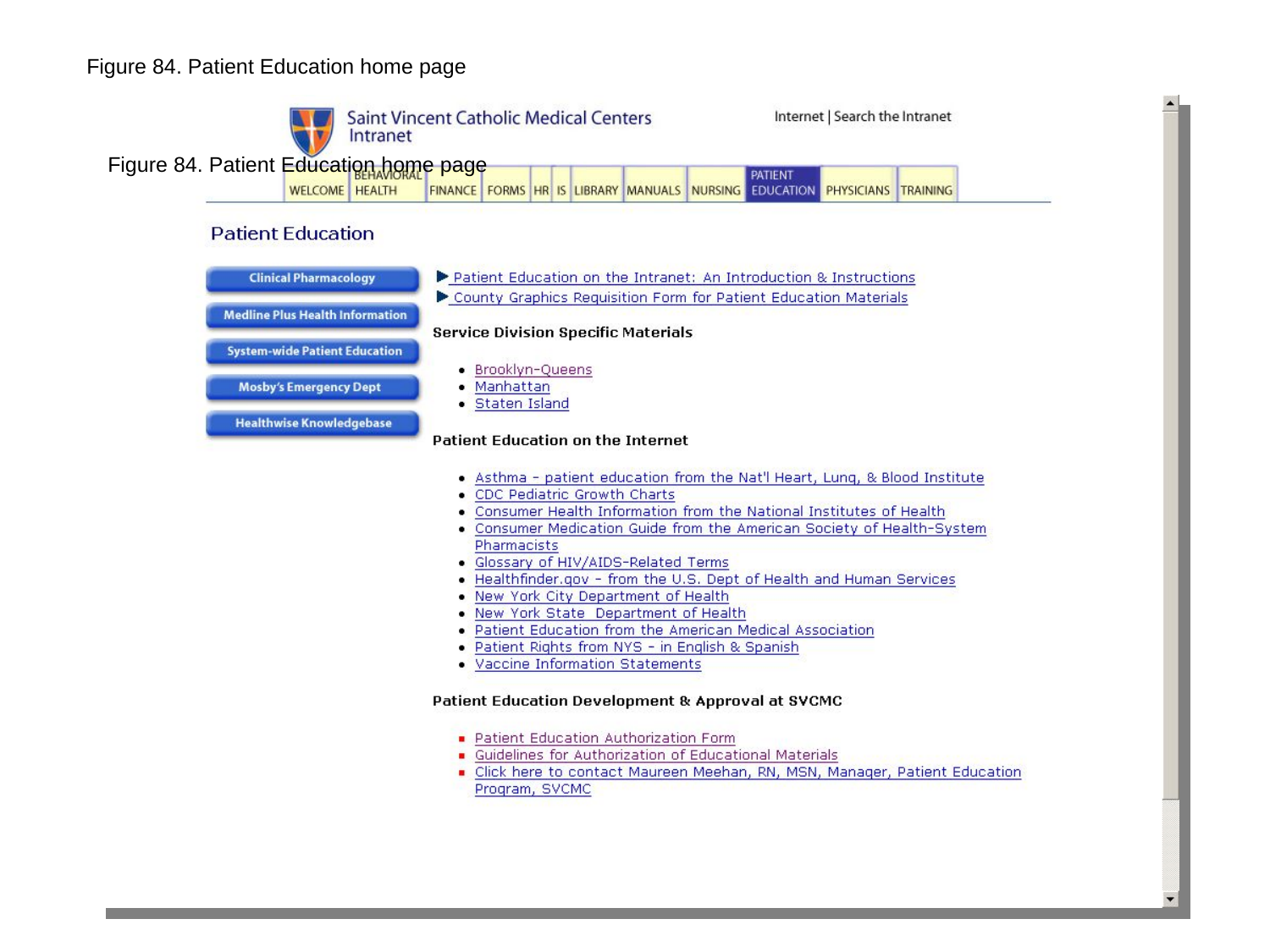

Figure 84. Patient Education home page
# Figure 84. Patient Education home page

## Slide 53
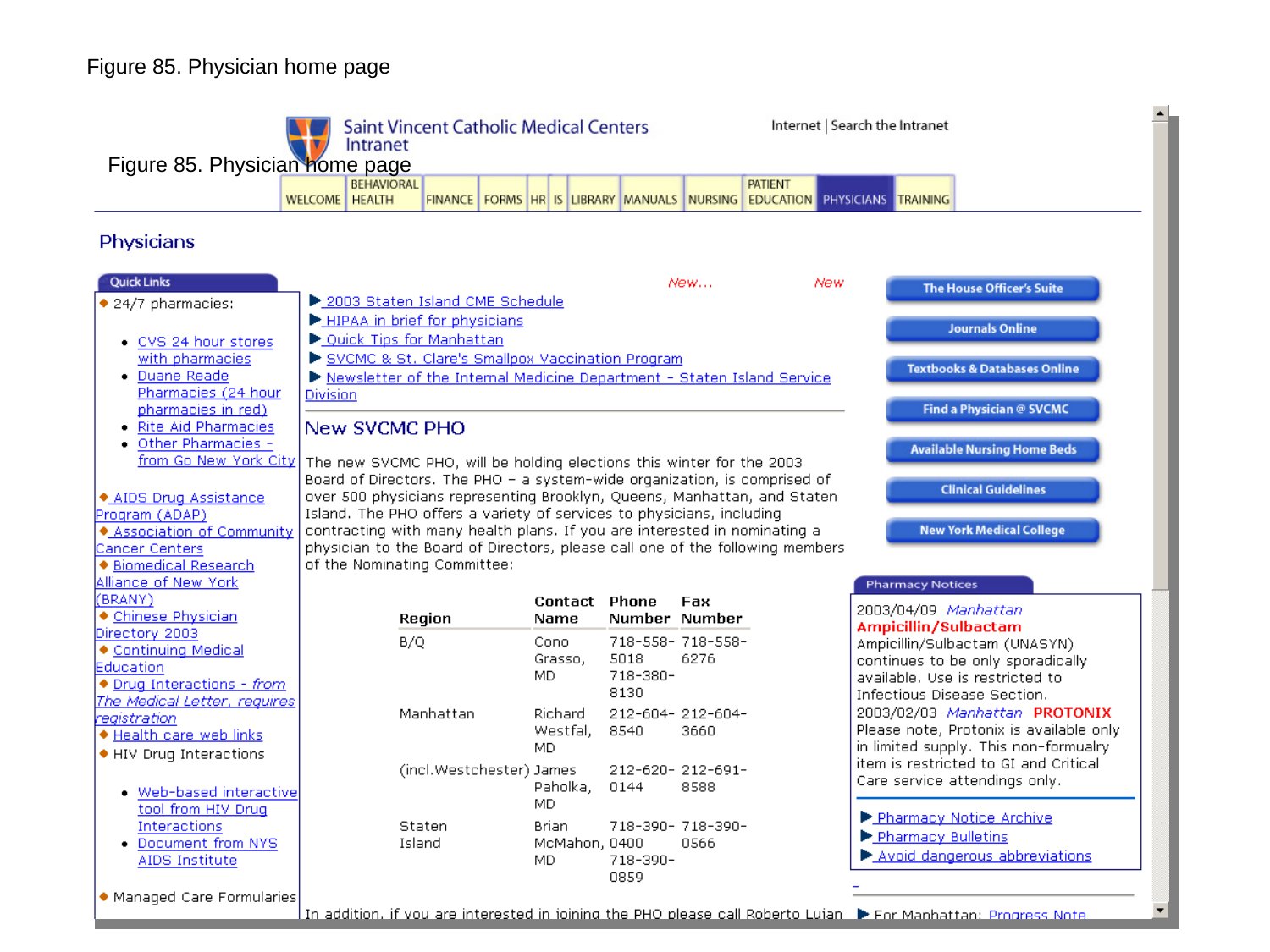

Figure 85. Physician home page
# Figure 85. Physician home page

## Slide 54
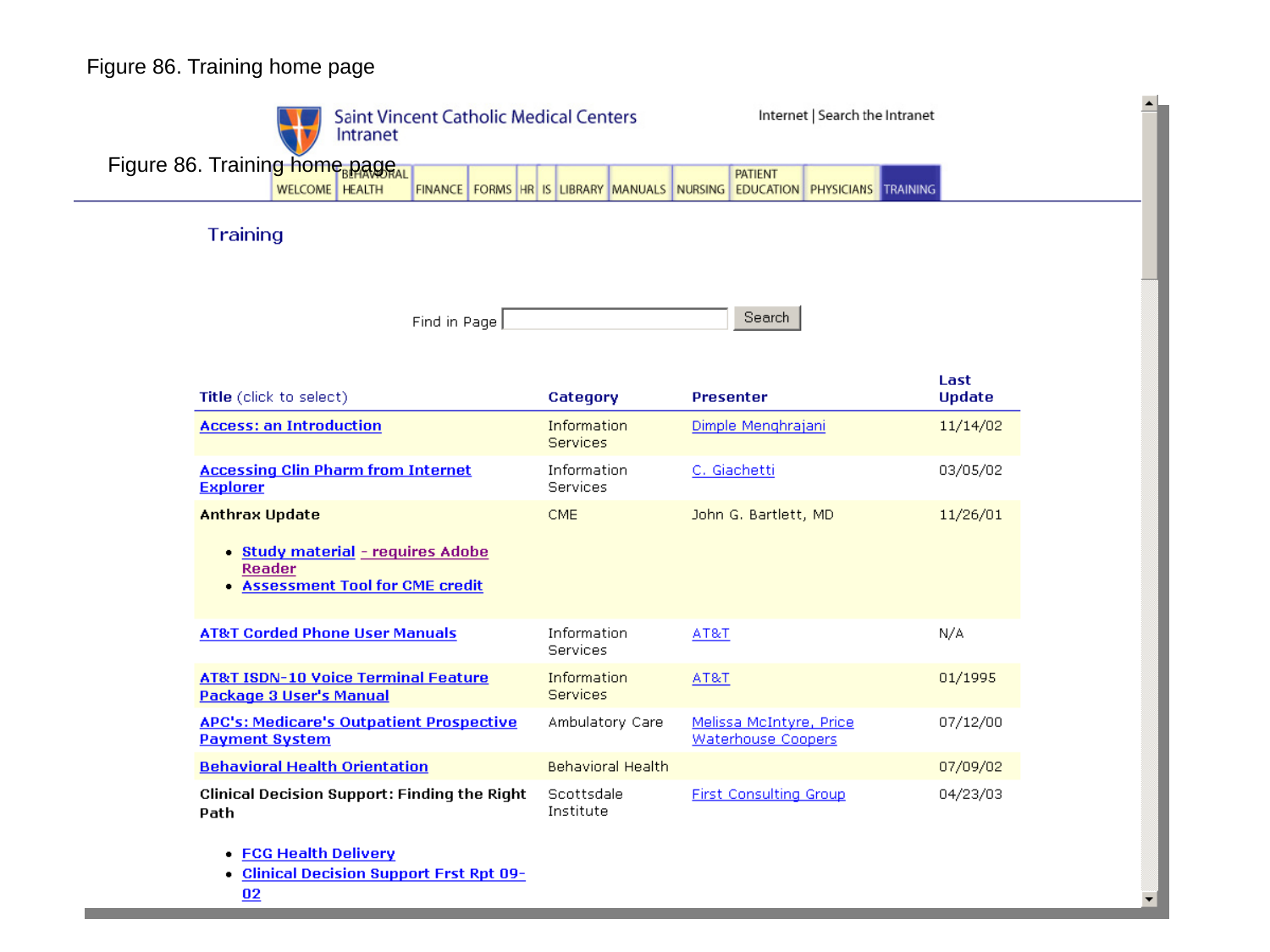

Figure 86. Training home page
# Figure 86. Training home page
